# Supplementary material for: A single-cell atlas of the human substantia nigra reveals cell-specific pathways associated with neurological disorders
Source: Nat Commun. 2020 Aug 21;11:4183. doi: 10.1038/s41467-020-17876-0 (PMC7442652; doi:10.1038/s41467-020-17876-0)
Supplement: Supplementary file 1 — Supplementary Information [file 41467_2020_17876_MOESM1_ESM.pdf]

A single-cell atlas of the human substantia nigra reveals cell-specific pathways associated with neurological disorders

Agarwal *et al.*

Supplementary Information

Supplementary Note 1

Single-cell atlas of the substantia nigra: all cell-types were mostly contributed to by all samples (**Supplementary Figure 1, Supplementary Figure 5**). Some sample-specific clustering and thus batch-specific effects can also be seen for reactive astrocyte-2 (N2B only, Day2) & ODC-3 (predominantly N2B) and this may be associated with heterogeneity between samples (**Supplementary Figure 2**, **Supplementary Table 3** and **Supplementary Table 1**). We found that neuronal populations have a higher UMI depth (median nUMI: DaNs = 8,419; GABA neurons =6,512) and gene coverage range (median nGene: DaNs= 3,579 ; GABA neurons= 3,529) than non-neuronal populations (median nUMI= 2,822;median nGene = 1,863)(**Supplementary Figure 1**).

Single-cell atlas of the cortex: the cortex cell-types were broadly classified into the major cell groups with the help of enriched marker genes and we resolved clusters as astrocytes (*AQP4*$^{+}$*, GFAP*$^{+}$), **Excitatory neuron**s (**Ex**)(*SATB2*$^{+}$*,SLC17A7*$^{+}$), Inhibitory neurons (In) (*GAD1*$^{+}$*,GAD2*$^{+}$) (see below), microglia(*CSF3R*$^{+}$*,PLXDC2*$^{+}$), oligodendrocytes cells (ODCs) (*MOG*$^{+}$*,MOBP*$^{+}$) and oligo precursor cells (OPCs) (*OLIG1*$^{+}$*,VCAN*$^{+}$) (**Supplementary Figure 4**, **Supplementary Data 3**). Similar to the SN, the major cell-types were contributed to by all samples, except for ODCs for which some sample-specific clustering (C1B & C3) can be seen (**Supplementary Figure 3**, **Supplementary Table 3**). Moreover, a uniform UMI depth, number of genes and cell-cycle phase trends were also observed across the cortex for most cell-types, except for the Ex neurons (median nGene= 3,559; nUMI=7,332) and microglia (median nGene= 3,759, nUMI=7,330) showing higher UMI depth and gene coverage than other cell-types (median nGene = 2,096; nUMI=3,599) (**Supplementary Figure 3**).

Supplementary Note 2

Joint clustering analysis of SN and cortex samples: Similarly using Seurat [2], a joint clustering of the CCA aligned cortex and SN nuclei was performed using the **transcripts per million** (**TPM**) expression matrix of only protein-coding genes which were log-transformed and linearly regressed for nUMI. As before we used the "FindVariableGenes" function, a minimum log(variance/mean) cutoff of 0.5 and maximum cut-off of 9 on the mean-variability plot to detect outliers and identify 4221 HVGs. A **principal component analysis** (**PCA**) was then performed across all nuclei only using the HVGs. The standard deviations of the PCs were plotted on to a scree plot to identify the first 25 PCs as significant that were used as input to identify cell clusters by the **shared nearest neighbor** (**SNN**) based graph approach at a resolution of 0.4 through the "FindClusters" function in Seurat. The identified clusters were then visualised as a **Uniform Manifold Approximation and Projection** (**UMAP**) plot with significant PCAs as input to understand the global relationship between similar cell-types across the cortex and SN (**Figure 1d**). We found that changing the number of significant PCs in the range of 15-30 and the clustering resolution in the range of 0.4-0.8 did not identify very different clusters.

Supplementary Note 3

Cell-type association methods: To reduce the chance of spurious conclusions, we performed cell-type association analyses with two polygenic approaches**, stratified LD score regression** (**LDSC**) method[15] and the **Multi-marker Analysis of GenoMic Annotation** (**MAGMA**) gene set analysis method[16]. The stratified LD score regression partitions the heritability from genome-wide association studies. (GWAS) summary statistics (**Supplementary Table 4**) according to different sets of genes specifically expressed in cell-types to identify disease-relevant cell-types. MAGMA performs gene-set enrichment analysis based on GWAS summary statistics while accounting for LD structure between SNPs. A competitive gene-set analysis linear regression model was performed using MAGMA to test the hypothesis that a cell-type-specific gene set has a greater mean association with the complex trait than the genes not present in the gene set. A previous study comparing these two methods has shown that MAGMA can identify more significant results or false positives than LDSC, which the authors believe could be due to uncorrected genomic confounding that can be corrected by including gene-level covariates[15]. While both methods are based on different assumptions and algorithms and could have different results for certain traits, in this study, both methods include gene-level covariates such as gene size, gene sample size and linkage disequilibrium in the regression models to correct for confounding biases. We showed here that globally, there is agreement between the results given by both methods. Based on the Spearman rank correlations of cell-type association strength ($-\log_{10}$ Pvalue) between LDSC & MAGMA for each complex trait, we observed a very similar overall ranking of the cell-types across the SN ($R=0.55$, $p<10^{-16}$) and the cortex ($R= 0.53$, $p<10^{-14}$) (**Supplementary Figure 13**) for most brain-related complex traits such as schizophrenia, major depression disorder and bipolar disease. Moreover, non-brain-related traits (e.g. Height) also, show high positive correlations across the two methods.

Cell-type-specific gene sets: To define cell-type-specific gene sets from the TPM expression matrix and the brain cell-types identified, we followed the approach of Finucane *et al.* 2018[15]. Here, for each gene a t-statistic is calculated between the expression of that gene in a given cell population as compared to its expression in all other cells, with cell-type-specific gene sets defined as the 10% of genes with the highest t-statistic in that cell-type (**Supplementary Data 4 and Supplementary Data 5**). Note that genes are not necessarily exclusive to a single cell-type. For the GTEx-brain tissue gene set, we used the GTEx brain gene set defined by Finucane et *al.* 2018[15] and downloaded the following file:

https://data.broadinstitute.org/alkesgroup/LDSCORE/LDSC_SEG_ldscores/GTEx_brain_1000Gv3_ldscores.tgz
.

Locus definition: On either side of the transcribed region of each gene in the set of cell-type specific genes, we extended the locus to define cell-(or tissue-) specific genomic region, aiming to capture regulatory elements that could affect the expression of that gene and mediate any GWAS variant effect on a trait. To define the size of this extension, we exploited the robust association between schizophrenia risk loci and genes specifically expressed in Ex neurons[17]. Thus, we ran LDSC [15] cell-type analysis with different window sizes (5kb,10 kb, 25 kb, 50kb,75 kb and 100 kb) to evaluate the association between schizophrenia risk loci and Ex neuron gene expression, and found that extending 25k around the transcribed region of each gene produced the most significant p-value for identifying Ex enrichment for schizophrenia (**Supplementary Figure 14**). We followed a similar approach for MAGMA[16] and ran the gene-level based trait analysis with multiple window sizes for schizophrenia GWAS[18], and evaluated the p-value for cell-type-specific association of cortical Ex and In neuron populations from our study with schizophrenia common genetic risk (**Supplementary Figure 15**). As with LDSC above, the most significant p-value for both Ex and In neurons was found to be 25 kb upstream and downstream of each gene and thus we used a 25kb window size for MAGMA analyses for all traits. Furthermore, we evaluated how the samples displaying amyloid angiopathy may affect the results of cell-type association analysis (individual 3, **Supplementary Table 1**) and found consistent results in the LDSC association for all different traits with and without nuclei of this individual: **Supplementary Figure 16** (SN: R=0.82 ($p < 10^{-16}$) Cortex: R=0.93(p< $p < 10^{-16}$)).

Conditional analysis for multiple cell-types within and across regions: Where LDSC identified multiple cell-types associated with the same trait, we used conditional analyses to evaluate whether it was the same genetic variants acting in different cell-types or distinct sets of genetic variants suggesting different cellular aetiologies. To test the association of cell-type A conditioned on cell-type B, we replaced the control gene set of all protein-coding genes expressed in both the cortex and nigral cell atlases by the cell-type B gene set and re-ran LDSC. When this conditional test produced a p-value associated with an LDSC Coefficient < 0.05, we considered that both cell-types could be involved independently in the same trait.

Correction for the cell-heterogeneity between cortex and SN: In the case of traits for which genetic variants were simultaneously associated with SN and cortical cell-type, potential bias was that the different proportion of glial versus neuronal cell-types between these two brain regions may lead to the identification of different gene markers for the same cell-type (e.g. microglia) and suggested erroneously two distinct cell-type signals. To discard this bias for the conditional analysis, we created SN and cortex cellular atlas including the same number of astrocytes, microglia/OPC/ODC/neurons (398 Astrocytes, 72 DaNs or Ex neurons, 123 GABA neurons or In neurons, 325 microglia cells, 160 oligodendrocyte precursor cells and 147 oligodendrocytes cells) by random sampling cells in the original cellular atlas and performed the same LDSC analyses as with the original cellular atlases. As the endothelial cells were only identified in SN and as we found no evidence of co-association for the endothelial cells with a cortical cell-type for no trait, we discarded the endothelial cells in the creation of these homogenous cellular atlases. Globally, we noted a good agreement with LDSC done with the original cell atlas (**Supplementary Figure 6**) (SN R=0.83 ($p < 10^{-16}$), cortex R=0.72 ($p < 10^{-16}$)), nevertheless we noted a p-value inflation in the microglia cells identified in the cortex for SCZ and bipolar disorder. We then repeated the conditional LDSC analysis for traits with SN and cortical cell-type enrichment (**Supplementary Table 8**, column P-value (LDSC) (Matched Cellular Atlas)).

An RMarkdown document, including a version with R code to generate these gene sets and perform cell-type association, can be found here: https://github.com/csandorfr/SN_Atlas.

Supplementary Note 4

***Functional Analysis - Protein Protein Interaction network**:* A combined protein-protein interaction network was created based on diverse resources: BioGRID 3.4 (accessed on September 2017) [19], HitPredict (accessed on September 2017) [20], IntAct (accessed on September 2017) [21], STRING (accessed on September 2017, restricted to Homo sapiens and experimental scores higher than zero) [22], CORUM (accessed on September 2017) [23] and Reactome (accessed on September 2017) [24]. The combined network consisted of a total of 20,591 genes and 1,973,967 interactions.

Supplementary Note 5

Nuclei extraction: nuclei extraction was based on the protocol described by Krishnaswami *et al.* [1] with some modifications. Briefly, 3mm^2 pieces of fresh-frozen brain tissue were placed in homogenization buffer (250 mM sucrose, 25 mM KCl, 5 mM $MgCl_2$, 10 mM Tris Buffer pH 8.0, 1 mM DTT, 1X protease inhibitor, 0.4 U ul^{-1} RNaseIN, 0.2 U ul^{-1} Superasin, 0.1% v/v Triton X-100) for 10 minutes prior to mechanical disruption with a dounce homogenizer. Homogenate was filtered through a 35 mM cell strainer before concentrating nuclei by centrifugation 400 g 5 minutes. Nuclei were resuspended in FACS buffer (1 XPBS, 1 RNase-Free BSA, 0.2 U µl^{-1} of RNasin Plus RNase inhibitor, 10 ng ml^{-1} Hoechst 33342) prior to FACS to obtain a population of nuclei free cellular debris.

Single nuclei processing and 10x genomics library preparation: nuclei were washed once in PBS and resuspended in 0.04% BSA in PBS for loading on the 10x Chromium 3' chip. Cell capturing, and library preparation was carried as per kit instructions (Chromium Single Cell Kit [v2 chemistry]). 3000 nuclei were targeted for capture per sample, after cDNA synthesis, 12-14 cycles were used for library amplification. The resultant libraries were size selected, pooled and sequenced using 75bp paired-end sequencing protocol on an Illumina HiSeq 4000 instrument. We used 12 samples for the 10X emulsions, with matched cortex and SN samples from 5 individuals with biological replicates for **substantia nigra** (**SN**) sample 4 (N4, N4B) and 5 (N5, N5B) run on two separate days (**Supplementary Table1**).

10x sequencing data processing: reads were processed and mapped to the Human Genome (GRCh38.84-premrna) with Cell Ranger 2.1.1. We used default mapping arguments for cellranger "count" function to merge multiple sequencing runs for the same sample libraries. In order to have counts from both unspliced/pre-mRNAs and mature mRNAs, a custom human (GRCh38.84) "pre-mRNA" reference was created, whereby each gene transcript was annotated as an exon, so that pre-mRNA intronic reads can also be included in the UMI counts for each gene and barcode. The filtered gene/barcode matrices containing only the detected cell-associated barcodes and UMIs produced as a result were then further analysed.

Supplementary Note 6

Dimensionality reduction and cell clustering analysis: Seurat R package (v2.3.4) was used to integrate different samples for the cortex and SN and then to perform a dimensionality reduction analysis. **Highly variable genes** (**HVGs**) were identified from a mean variability plot (average expression versus dispersion (variance/mean) assigned to 20 bins based on average expression) using a log(variance/mean) cutoff of 0.5 and maximum cut-off of 3.5 to identify outliers for each sample. Within each bin, a z-score of log-transformed dispersion measure (variance/mean) was calculated. A z-score cutoff of 0.5 was applied to identify the highly variable genes for each sample and the union of top 3000 of these from each sample resulted in 1031 and 2243 genes in the SN and cortex, respectively. A **canonical correlation analysis** (**CCA**) [2] was performed and after discarding rare – non-overlapping cells, the top numbers of CCA dimensions to align were identified by examining the correlation strength of all vectors through the biweight midcorrelation, a median based similarity metric saturation plot. This enabled to identify 25 and 42 canonical correlation vector dimensions to align for the SN and cortex.

The shared-nearest neighbour graph was constructed on a cell-to-cell distance matrix firstly by calculating the k-nearest neighbours with k=30 from the aligned CCA vectors. These were then used as input to the Louvain algorithm [3] with different clustering resolutions for the SN and cortex to identify cell-type clusters. We performed the analysis with different resolutions in Seurat by sequentially increasing the resolution from 0.4 to 2.4 and

assessing the clusters by constructing a phylogenetic tree for the averaged cluster populations at each resolution based on the HVG genes. For each branch of the subsequent tree a random forest classification error was calculated and an accuracy threshold of 80% was used to assess tree splits and cluster validity. This resulted in an optimal resolution parameter for the SN and cortex to be 0.4 and 0.8, respectively.

Supplementary Note 7

Cortex neuronal subtypes: Lake e*t al* [4, 5] have previously identified subtypes of Ex and In neurons based on specific markers that we used here to further, identify nine subtypes of Ex neurons and ten subtypes of In neurons drawn from across different cortical layers.
Neuronal subtypes were resolved into Ex and In neurons based on known marker genes *SATB2, SLC17A7, GAD1 and GAD2* (**Supplementary Figure 4**, **Supplementary Data 3**). The Ex neurons could be further classified based on their layer positions and known marker genes. A population of Ex2 subtype expressing the L2/3/ *CUX2* & L4 specific (*RORB*$^{+}$) markers and *COL5A2*$^{+}$ was the most prevalent in our dataset (19%, **Supplementary Table 3**), followed by the second most prevalent, L2-3 subpopulation (*LAMP5*$^{+}$), Ex1 (14%, **Supplementary Table 3**) showing *CBLN2*$^{+}$, *CUX2*$^{+}$ expression. While we did identify layer 4 subpopulations, Ex3 (*RORB*$^{+}$, *COL5A2*$^{-}$ *,IL1RAPL2*$^{+}$*)* and Ex4 (*RORB*$^{+}$*,TPBG*$^{+}$) like Lake *et al* [5], (**Supplementary Figure 4**), we were not able to further distinctly resolve this sub-population into Ex3 (a-d) subtypes. We also, identified distinct subpopulations located in cortical L5*(PCP4*$^{+}$*, ETV1*$^{+}$), Ex5a (*PCP4*$^{+}$*,HS3ST5*$^{-}$), Ex5b (*PCP4*$^{-}$*,HS3ST5*$^{+}$) and Ex6a (*HTR2C*$^{+}$*,PCP4*$^{+,}$*TLE4*$^{+}$), with the last Ex6b (*HTR2C*$^{-}$, *TLE4*$^{+}$*,SEMA3D*$^{+}$) bordering on cortical L6 /6b or white matter(*NR4A2*$^{+}$) (**Supplementary Figure 5**). Lastly, the least prevalent (0.59%) cortical neuronal subpopulation we identified was Ex8 (*TLE4*$^{+}$*,NTNG2*$^{+}$*)* localised to L6.

In neuron, the subtypes were discriminated from the other cell-types by expression of *GAD1* and *GAD2* marker genes (**Supplementary Figure 4**) and further resolved into 10 subpopulations based on enriched marker gene expression that showed distinct profiles of established In neuron marker genes (**Supplementary Data 3**). We resolved spatially distinct *CNR1* expressing L1-2 subpopulations as In1a (*RELN*$^{+}$), In1b (*THSD7B*$^{+}$), In1c (*VIP*$^{+}$*,TAC3*$^{+}$*)* and In3 (*VIP*$^{+}$*,TSHZ2*$^{+}$), but not the upper layer In1d and In2 subtypes (**Supplementary Figure 4**). We further identified distinct *SV2C* expressing L2-3 (*LAMP5*$^{+}$) subtypes as In4a (*COL5A2*$^{+}$), In4b (*COL5A2*$^{-}$ *,EYA4*$^{+}$) and In5 (*EYA4*$^{+}$ *NOS1*$^{+}$) (**Supplementary Figure 4**); layer 4/5 (*SULF1*$^{+}$) located subtypes expressing *PVALB*, In6a (*RYR1*$^{+}$) [5] and the more peripheral and the most prevalent (8.65%) In neuron subtype in our cortical cell atlas In6b (*TAC1*$^{+}$) ; and finally *SST* expressing L6 (*SYNPR*$^{+}$) subtype In8 (*STXBP6*$^{+}$) but not the other *SST* -positive In7 sub-population [5] (**Supplementary Figure 4**).

Supplementary Note 8

Laser- capture microscopy cell-types in the SN: Microarray profiles of laser-captured human dopaminergic neuron from control and PD patients were obtained from GEO (GSE24378) [6]. We downloaded the raw intensity values and performed a **Robust Multi-array Average** (**RMA**) transformation on probe-level with function RMA of R library oligo[7], and computed the expression by gene by using the mean of expression probe measures, which were then log-transformed. Only the 9 control samples were used for the comparison.

Fastq files for RNA-seq data of **laser- capture microscopy** (**LCM**) astrocytes and microglia for a total of 18 samples for each cell-type in the SN[8] were downloaded from **National Institute on Aging Genetics of Alzheimer's Disease Data Storage Site** (**NIAGADS**) (NG00057). The paired-end sequencing data for each sample were mapped to the human reference transcriptome (GRCh38; Ensembl release 91) and quantified transcript abundance counts were obtained with the tool Salmon (v 0.11.0) [9], using mapping based mode with default parameters, automatic library type inferring and additional options --seqBias,--posBias & --gcBias to account for sequence-specific biases, fragment -level GC biases and to account for 5' or 3' positional biases in the data. The transcript abundances were imported and summarized to gene-level using the R library tximport[10]. Only the protein-coding genes were used and data were filtered to include genes with >20 counts across all samples. The data were then normalised using the **variance stabilizing transformation** (**VST**) with vst function in the R library DESeq2[11]. Sample outliers were removed after visualising VST transformed data as PCA plots. Technical effects associated with the total number of genes were regressed out by using the removeBatchEffect function in the R library limma[12]. Finally, vst-transformed data for the control samples (n=9) for the LCM astrocytes and microglia were taken forward for comparison with the averaged log-transformed TPM 10x SN cell-type expression data. We discarded protein-coding genes that were not expressed in all three datasets(10x SN,

LCM DaN & LCM astrocytes and microglia) used to perform these comparisons: 11,703 protein-coding genes were used in the comparisons for 28 samples/cells.

Observing that the distributions of gene expression levels were different between the datasets even after the ComBat batch correction of the R library sva[13], we applied a non-parametric approach by ranking the genes according to their expression level in each tissue/cell. From this rank matrix, we computed pairwise spearman correlations between LCM cell-types/cell and performed clustering analyses by using Ward's hierarchical approach and visualised the resulting matrix as a heatmap (**Supplementary Figure 12**).

Single cell -RNA sequencing Temporal cortex: For comparison with single-cell RNA-seq data from human **temporal cortex** (**TC**) [14], we obtained gene count data from GEO (GSE67835) and converted these to TPM counts and restricted the analysis to protein-coding genes (n= 15,081) and cells (n= 465), maintaining the cell-type annotations from the original publication[14]. The expression matrix was then normalized and linearly regressed for batch identity with Seurat as described above, using a minimum cutoff of 1,000 protein-coding genes detected in at least three cells. HVG were identified from a mean variability plot (average expression versus dispersion (variance/mean) assigned to 20 bins based on average expression) using a log(variance/mean) cutoff of 0.5 to identify 3,662 genes. Moreover, differentially expressed genes associated with each cell-type (excluding the hybrid cell-type) were identified by Seurat, using parameters and thresholds as above. Transcriptional Pearson correlations were calculated in a pairwise manner across the averaged cell-type populations from the external and 10x datasets, grouped via Ward's hierarchical clustering and visualised as a heatmap (**Supplementary Figure 12)**. The final comparison was restricted to log-transformed averaged cell-type TPM expression values of 6,446 genes, composed of HVG (protein-coding only) from both datasets along with the top 5 enriched genes (logFC > 2) in each cell-type.

Single nuclei -DropSeq visual and frontal cortex: We obtained the UMI counts for the **visual cortex** (**VC**) and **frontal cortex** (**FC**) single nuclei drop-seq data from GEO (GSE97930) and restricted the analysis to protein-coding genes (n= 10,919) and annotated nuclei (n= 29,441) maintaining cell-type annotations from the original publication[5]. As above, the expression matrix was then loaded into Seurat and nuclei with fewer than 300 UMI counts or more than 5000 UMI counts were omitted and further filtering to only retain nuclei with a minimum of 200 genes detected in at least 1% of cells was also, performed. We normalized and linearly regressed for technical effects associated UMI coverage and batch identity like the original publication with Seurat as above. HVGs were identified from a mean variability plot using a log(variance/mean) cutoff of 0.5 to identify 2,181 genes. Moreover, differentially expressed genes associated with each cell-type were identified by Seurat, using parameters and thresholds as above. Transcriptional Pearson correlations were calculated in a pairwise manner across the averaged log-transformed cell-type and subtype populations expression values from the external and 10x cortex dataset, grouped via Ward's hierarchical clustering and visualised as a heatmap (**Supplementary Figure 12**). The analysis was restricted to 3,614 genes composed of HVG (protein-coding only) from both datasets and top 5 differentially expressed genes (logFC > 2) defining each cell-type.

Supplementary Figures

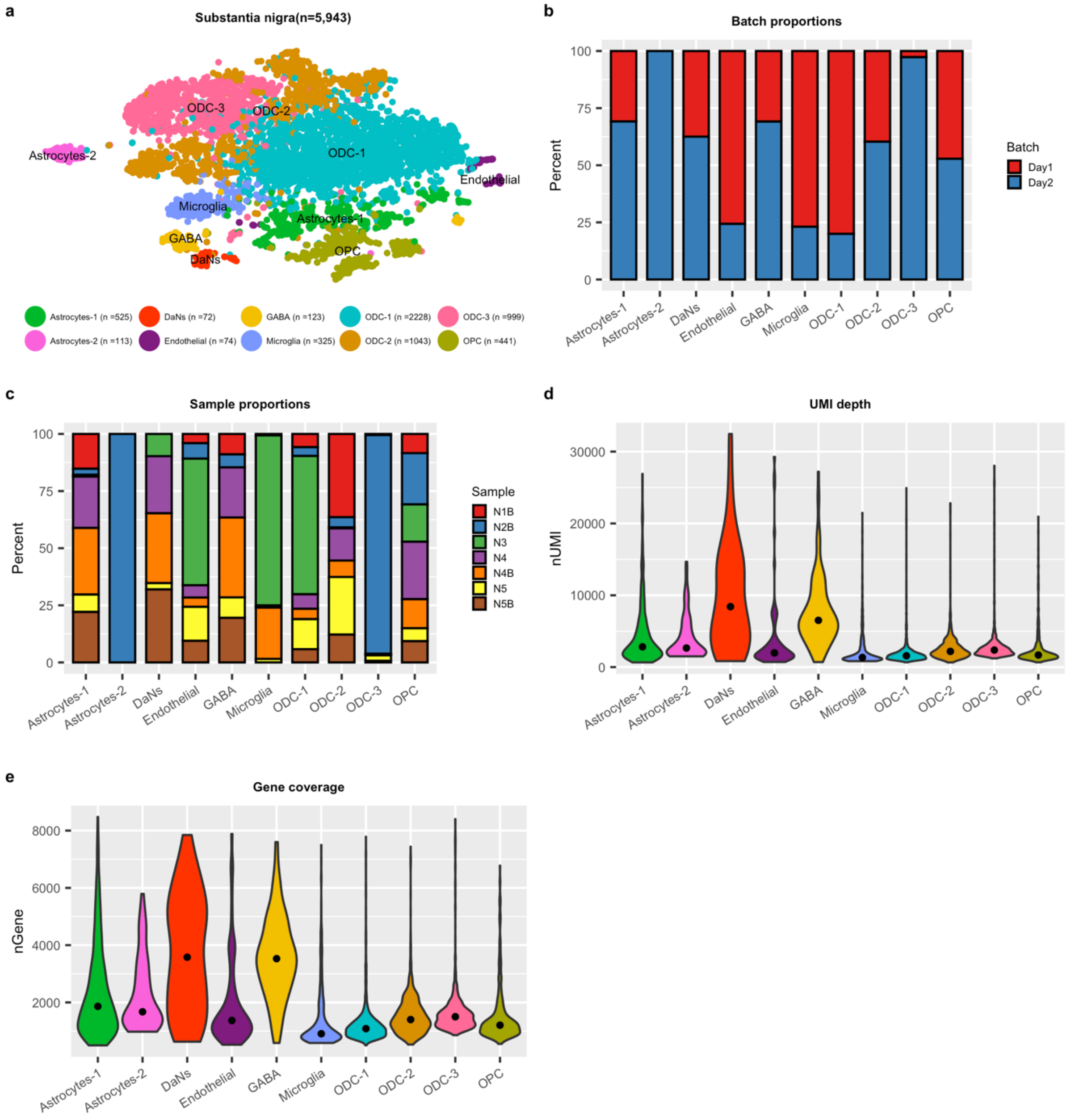

Supplementary Figure 1: Covariate distributions in the substantia nigra (SN) cell atlas.

a) T-distributed stochastic neighbor embedding (tSNE) plot for all non-neuronal (astrocytes, endothelial, microglia, ODC and OPC) and neuronal (DaNs and GABA) cell-types or subtypes across the SN. **b)** barplot plot showing batch proportions per level-2 cell-type**. c)** barplot as in (b) showing sample proportions per level-2 cell-type. **d)** Violin plot showing unique molecular identifier (UMI) depth across all level-2 cell-types. **e)** Violin plot as in (d) showing gene coverage across all level-2 cell-types. In (d) and (e) the black dot denotes the median UMI and genes for the cell-type. DaNs: Dopaminergic neurons; GABA: GABAergic neurons; ODC: Oligodendrocytes; OPC: Oligo-precursor-cells.

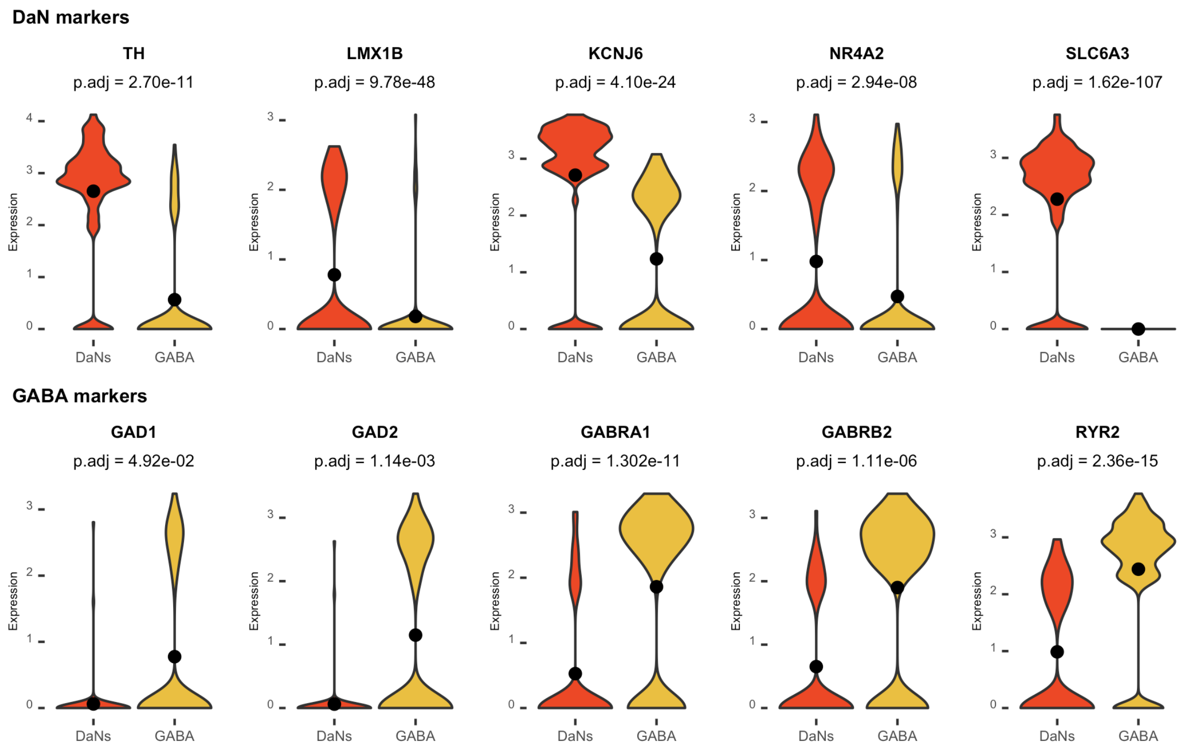

Supplementary Figure 2: Violin plots of DaN and GABA neuron markers in the SN neurons.

Violin plots of expression values (log10 TPM values) for known DaN and GABA markers. Differential expression p-values after Bonferroni correction(p.adj) using the two-sided negative binomial test in Seurat were calculated between the two neuronal populations (Methods & data S3). In each of the violin plots the black dot denotes the mean expression for the marker genes for the cell-type. DaNs: Dopaminergic neurons; GABA: GABAergic neurons.

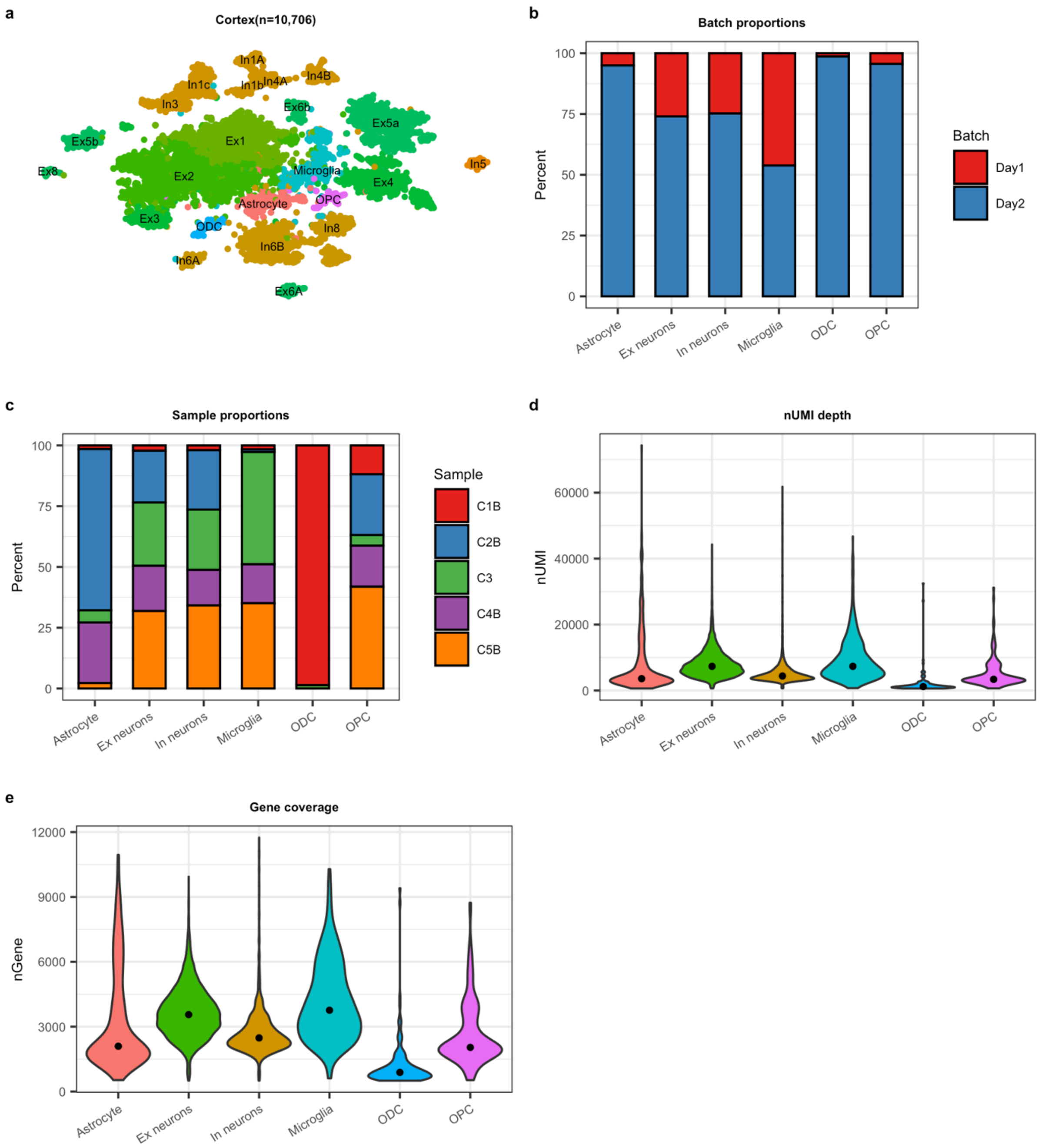

Supplementary Figure 3: Covariate distributions in the cortex cell atlas.

a) T-distributed stochastic neighbor embedding (**tSNE**) plot for all non-neuronal (astrocytes, microglia, ODC and OPC), Ex and In neuron cell-types or subtypes across the cortex. **b)** barplot plot showing batch proportions per level-1 cell-type. **c)** barplot as in **(b)** showing sample proportions per level-1 cell-type. **d)** Violin plot showing unique molecular identifier (**UMI**) depth across all level-1 cell-types**. e)** Violin plot as in **(d)** showing gene coverage across all level-1 cell-types. In **(d)** and **(e)** the black dot denotes the median UMI and genes for the cell-type. Ex:Excitatory;In:Inhibitory ;ODC: Oligodendrocytes;OPC: Oligo-precursor-cells.

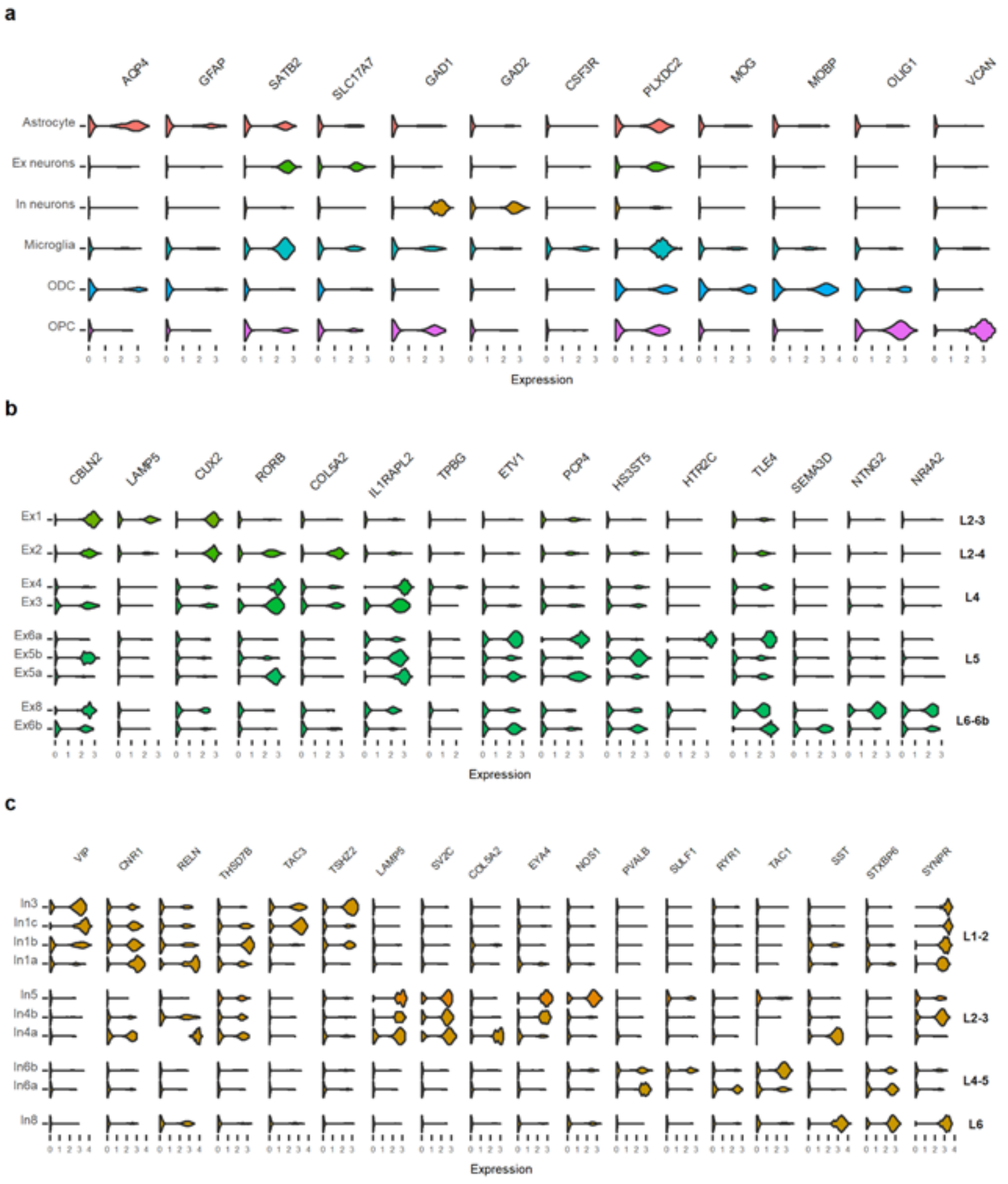

Supplementary Figure 4: Cortex cell-types:

(a) Violin plots of expression values (log10 TPM values) of cell-type specific markers for the cell-types in the cortex. (b) Violin plots of expression values (log10 TPM values) of cell-type and cortical layer-specific enriched marker gene profiles[6] for the Excitatory neurons subtypes. (c) Violin plots of expression values (log10 TPM values) of cell-type and cortical layer-specific enriched marker gene profiles for the Inhibitory neuron subtypes. Ex: Excitatory; In: Inhibitory, ODC: Oligodendrocytes; OPC: Oligo-precursor cells, L1-2; Layer 1/2, L2-3; Layer 2/3,Layer 2-4; Layer 2/3/4, L2 ; Layer2, L3; Layer 3, L4; Layer 4, L4-5; Layer 4/5, L6; Layer 6; Layer 6-6b; Layer 6/6b

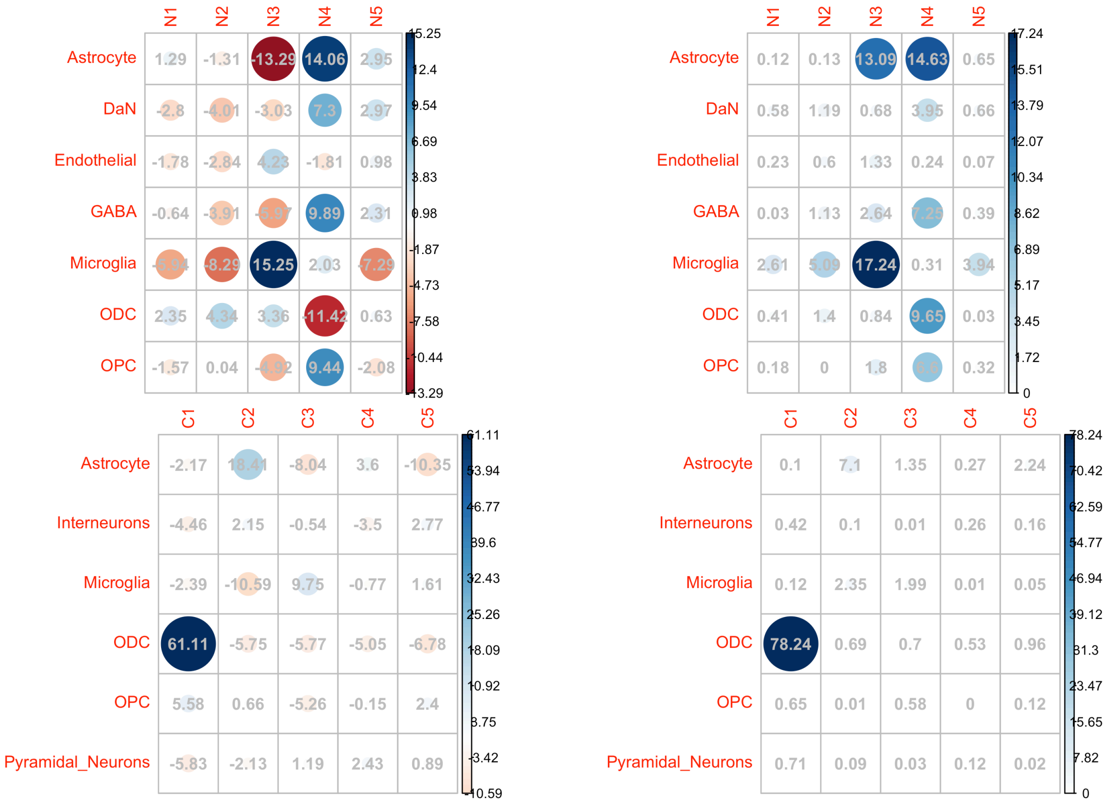

Supplementary Figure 5: Only the minor cell populations show difference in the frequency between the individuals.

Cell composition (left) & contribution (right) of each individual within SN (top) and cortex (bottom) single nuclei atlas. (Left graph) within each atlas, we calculated the Pearson residual reflecting an under (red) or over-representation (blue) of a specific cell-type for an individual. (Right graph) we calculated the % represented by chi^2 residual reflecting whether an individual contributes more to a cell-family category than another individual. The size of the circle is proportional to the absolute value of the Pearson residual or the % represented by chi^2 residual.

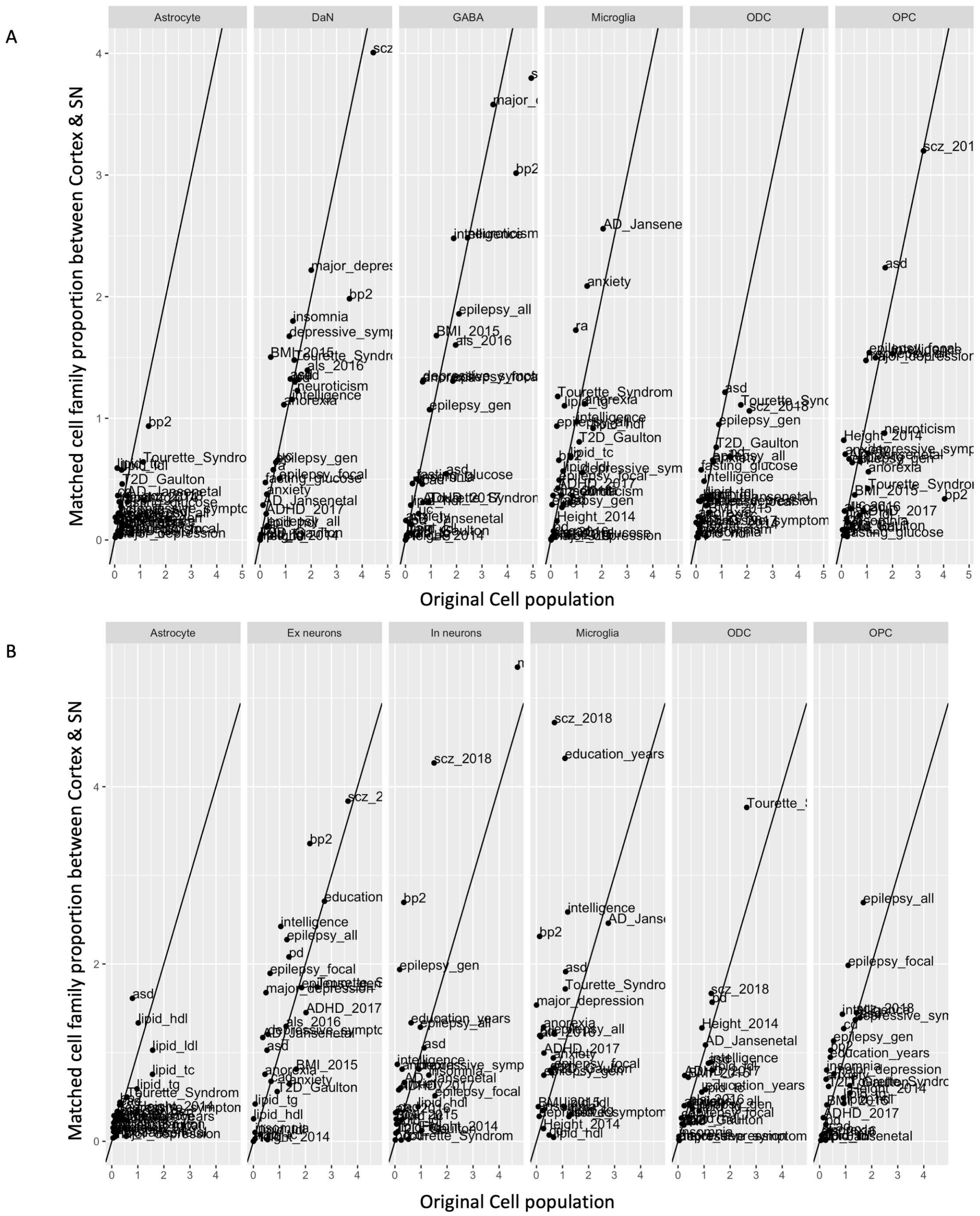

Supplementary Figure 6: Comparison of cell-type enrichment analyses performed with LDSC for different traits between the cellular atlases with matched proportions of cell types between cortex and SN and the original cellular atlases

From the original cellular atlas, we created SN (A) and cortex (B) cellular atlas including the same number of astrocytes microglia/OPC/ODC/Neurons by random sampling cells in the original cellular atlas and performed the same LDSC analyses as with original cellular atlases (Methods). For a specific cell-type, the x-axis and y-axis of each plot represent the -log 10 p-value for enrichment of genetic variants associated with human trait by using the original cellular atlases and the matched cell family proportion cellular atlases respectively.

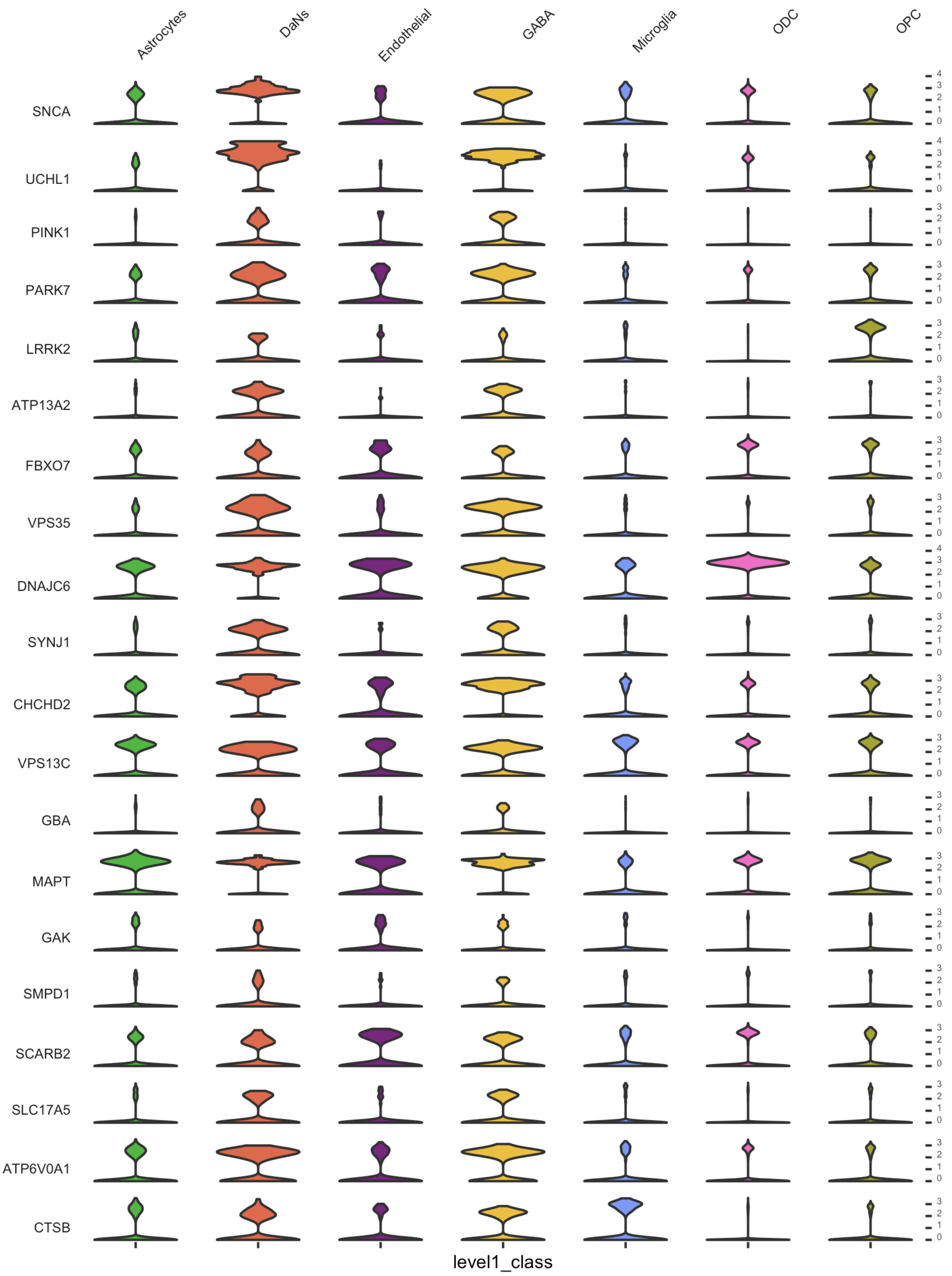

Supplementary Figure 7 : Expression of known Parkinson's disease genes in SN level-1 cell-types.

Violin plots of expression values (log10 TPM values) of Parkinson's Disease (PD) risk genes[26] for the cell-types in the SN.

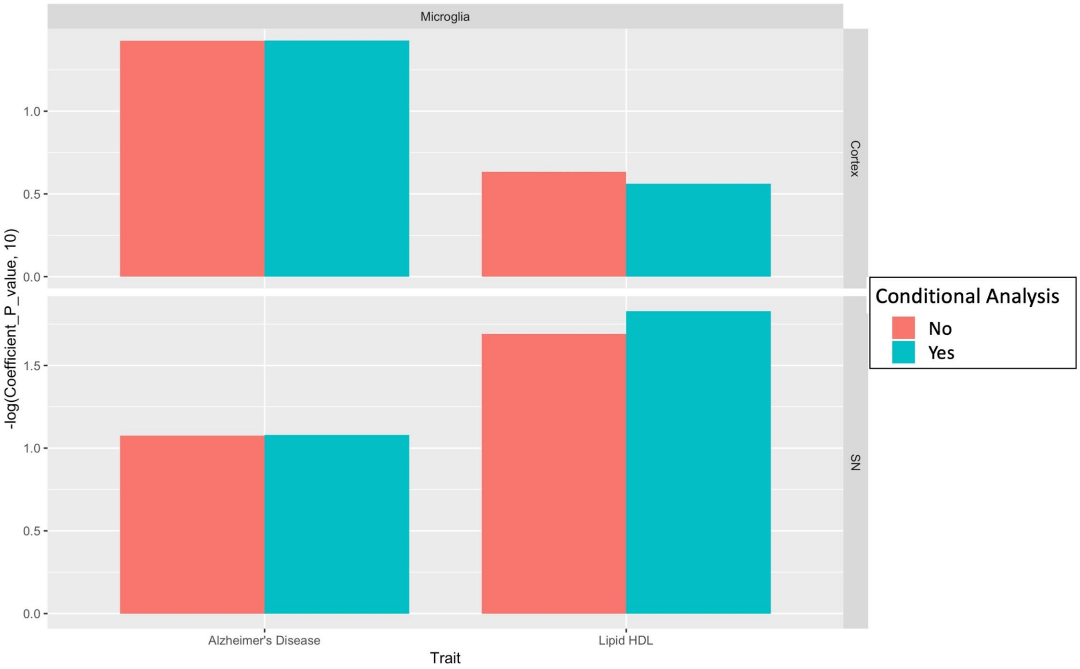

Supplementary Figure 8: The microglia-specific signals for the genetic variants associated with HDL cholesterol level and AD correspond to distinct genetic aetiologies.

LDSC analysis by using the AD and HDL GWA summary statistic adjusted (green) or not (red) for HLD and AD risk effect respectively to evaluate the cell-type association within microglia in the cortex (top) and SN (bottom). Each bar represents the -log 10 p-value of enrichment genetic variants associated (p-value associated with an LDSC Coefficient) with AD or HDL (adjusted or not for the other trait) for microglia cell-type identified in the cortex or SN.

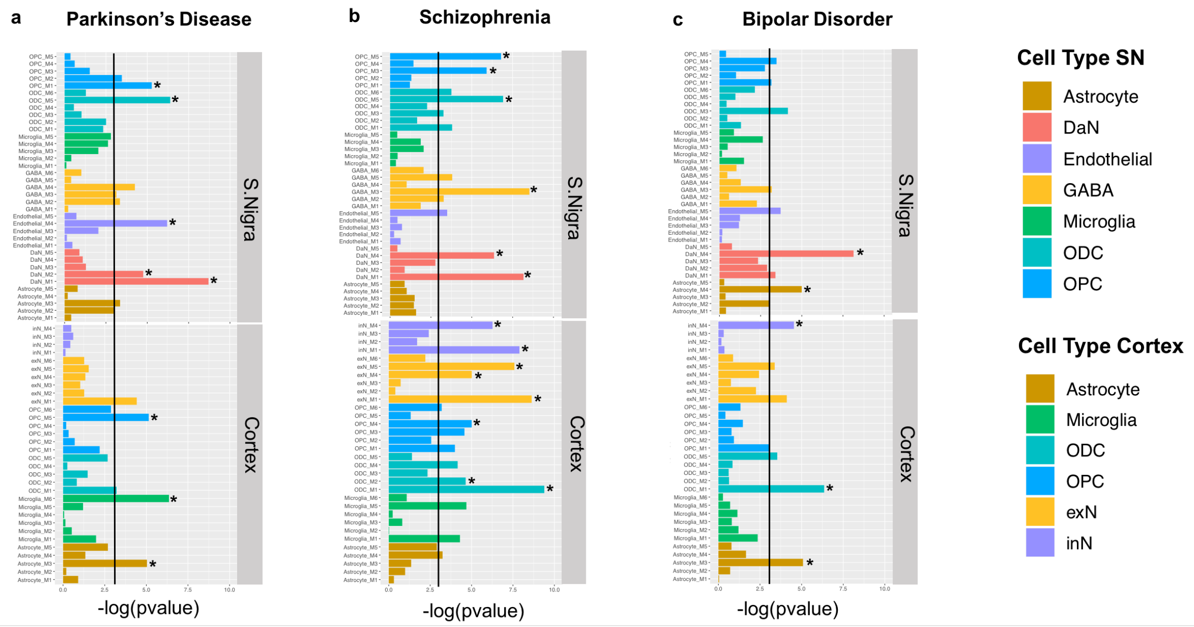

Supplementary Figure 9: Convergence of PD-risk (a), SCZ-risk (b) and BP-risk (c) across SN and cortex cell-type specific gene modules.

We assessed SCZ-, PD- and BP- risk enrichment in cell-type specific gene modules from cortex and SN through MAGMA gene set analysis (one -sided positive two -sample t-test). Barplots represent -$\log_{10}$ p-values of enrichment, vertical lines correspond to p-value=0.05 and star symbols (*) indicate significant q value (Bonferroni correction for the number of cell type PPI modules tested). Only the associations to modules within cell-types that showed significance from the more general analysis (Figure 2) were discussed further.

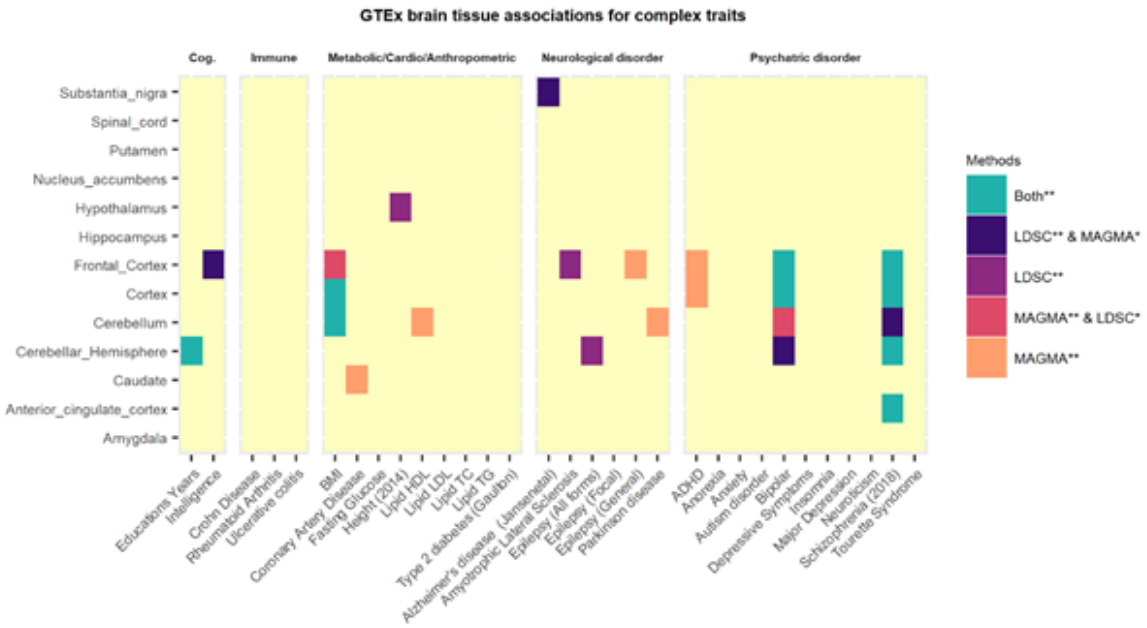

Supplementary Figure 10: Tissue-trait associations across diverse human complex traits using GTEx bulk brain tissue transcriptomic data.

Two classical approaches: stratified LD score regression (LDSC) (p-value associated with an LDSC Coefficient) and the MAGMA gene set analysis (one -sided positive two -sample t-test) have been used to identify the association between multiple complex traits genome wide association signals within 13 brain regions from bulk GTEx transcriptomic data. The heatmap colour indicates if the p-value of cell-type enrichment for a trait is significantly associated with both methods or either methods after Bonferroni multiple test correction(**) and also, if nominally significant (p<=0.05) in the other method (*). Bonferroni threshold was P-value/No.of.cell-types. The different traits have been clustered by category: Cognitive phenotype (Cog.), Autoimmune disease (Immune), Metabolic, Cardiovascular and Anthropometric Traits (Metabolic/Cardio/Anthropometric) Neurological disorder, Psychiatric disorder.

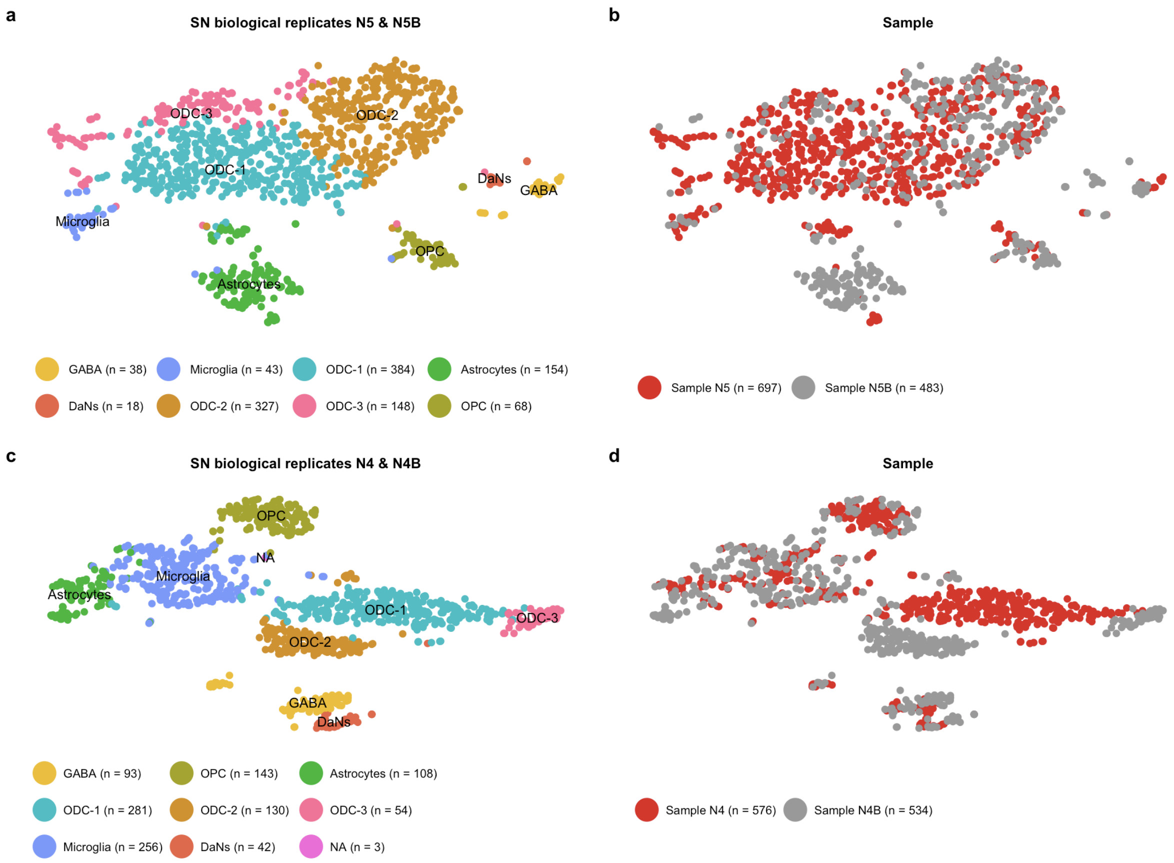

Supplementary Figure 11: Evaluation of the similarity of Substantia nigra (SN) biological replicates from the same individuals.

a) T-distributed stochastic neighbor embedding (tSNE) plot for all non-neuronal (astrocytes, microglia, ODC and OPC) and neuronal (DaNs and GABA) cell-types or subtypes across biological replicates N5 and N5B in the SN. b) t-SNE plot as in (a) showing sample identity N5 and N5B (**Supplementary Table 1**). c) t-SNE as in (a) showing cell-types for N4 and N4B SN biological replicates (**Supplementary Table 1**). d) t-SNE as in (c) showing sample identity N4 and N4B (**Supplementary Table 1**). All major cell-types were identified in both sets of replicates from each individual. In both sets of replicates a distinct cluster of endothelial cell-type was not identified and in N5&N5B a small cluster of N=3 was not attributed to any particular cell-type. DaNs: Dopaminergic neurons; GABA: GABAergic neurons; ODC: Oligodendrocytes; OPC: Oligo-precursor-cells ; N4: Nigra 4;N4B;Nigra 4B;N5:Nigra 5; N5B: Nigra 5B.

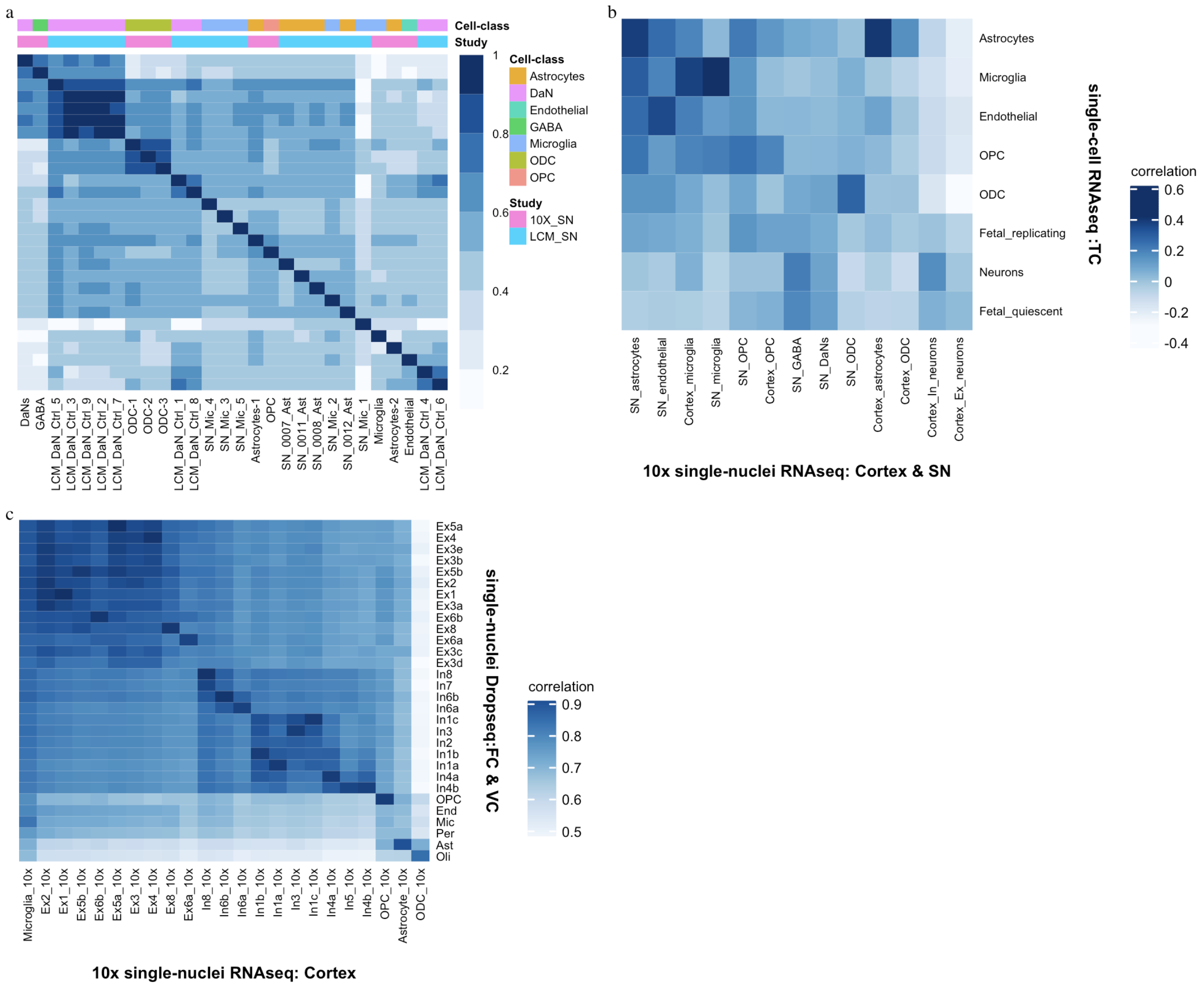

Supplementary Figure 12: Comparison of Substantia nigra and cortex (10x) cell-types with public transcriptomic datasets

a) SN laser- capture microscopy (LCM) Cell-types. A Spearman correlation heatmap comparing averaged SN level-2 cell-type populations with LCM SN Dopaminergic neurons (LCM_DaN_Ctrl) [6], microglia (Ctrl_Mic) and astrocytes (Ctrl_Ast) [8] from control samples. b) Cortex single cell RNA-seq data. Pairwise Pearson correlation comparison of the averaged 10x cortex and SN neuronal and non-neuronal subtype populations with the averaged temporal cortex (TC) single cell RNA-seq type populations c) Cortex single nuclei Drop-seq data. Pairwise Pearson correlation comparison of the averaged 10x cortex neuronal and non -neuronal subtype populations with the averaged cell-type populations in the frontal cortex (FC) and visual cortex (VC) of this single nuclei dataset.

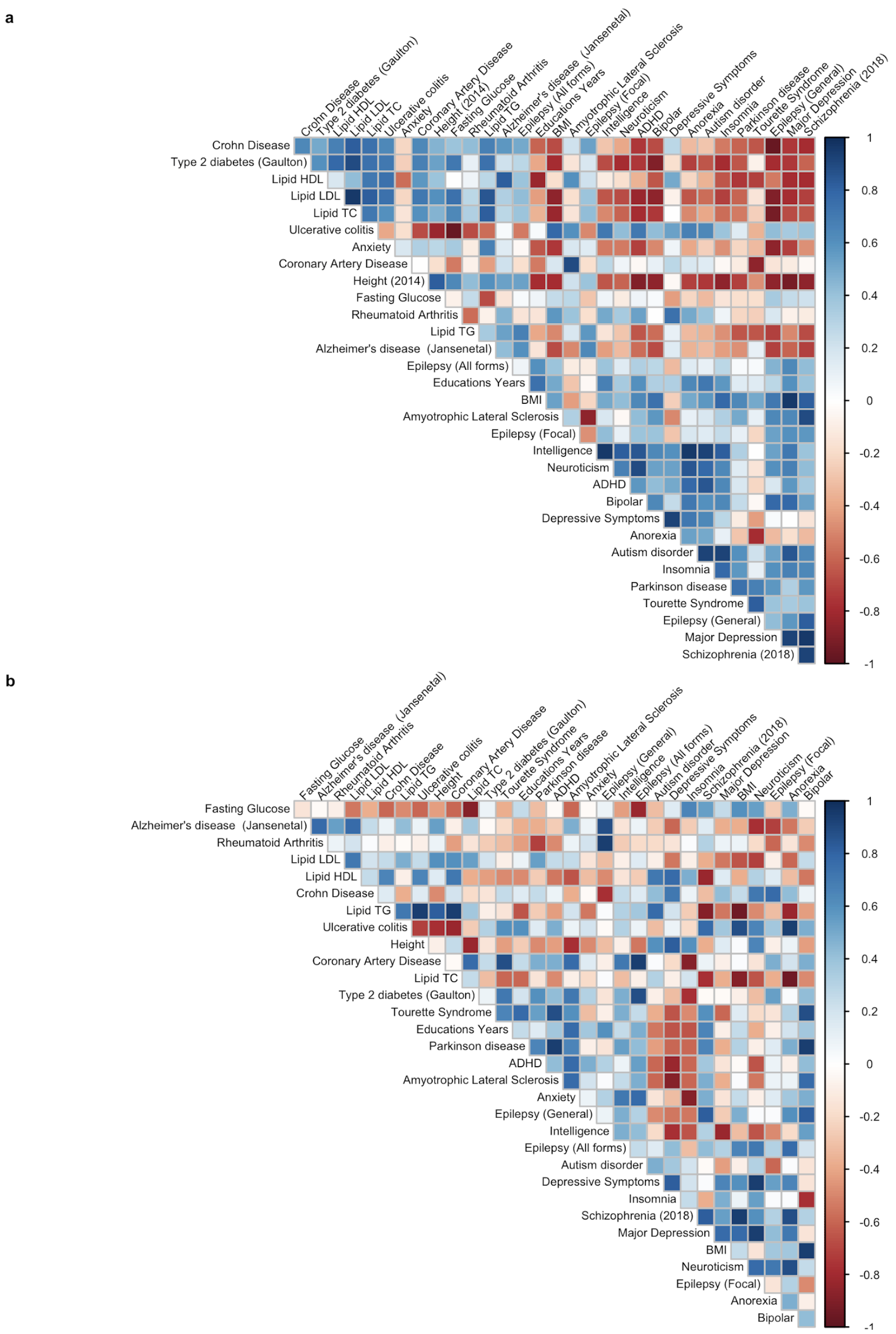

Supplementary Figure 13: LDSC vs MAGMA spearman rank correlation plot for cell-type associations (-log10 pvalues) in the SN and cortex cell atlas.

The clustered Spearman rank correlations between the rank orders of cell-type associations(-log10 Pvalue) for LDSC and MAGMA across traits for the a) SN and b) cortex cell-types are shown. A moderate to high positive correlation was observed for neurological disorders such as PD and ALS, for psychiatric disorders such as Schizophrenia, ADHD, Autism Disorder, Major Depression and Insomnia. Moreover non-brain metabolic traits such as Height, BMI and Cholesterol (HDL & LDL) and immune traits (Crohn's disease) were also, highly concordant between the two methods.

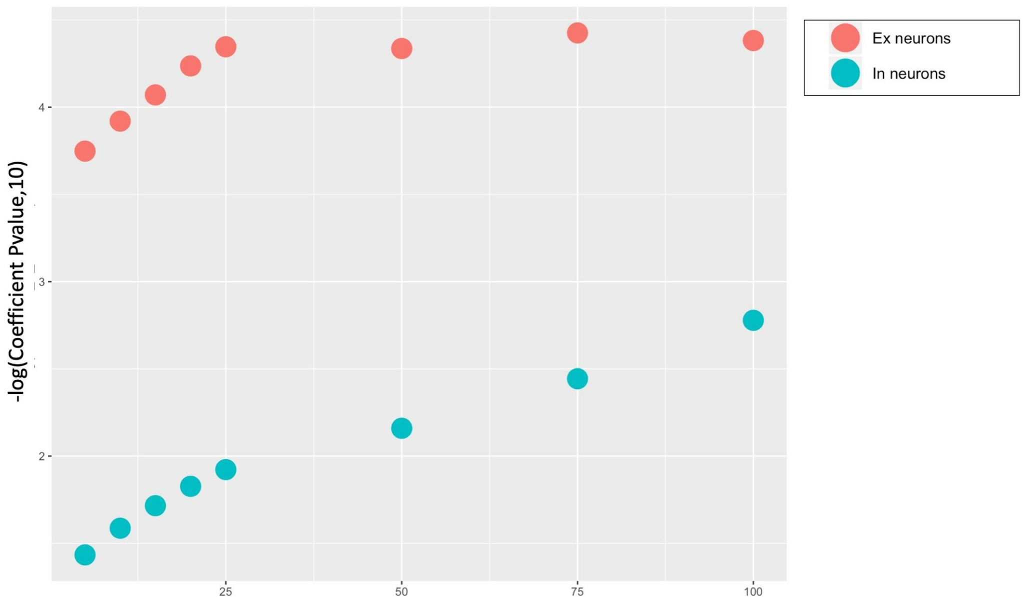

Results of the cortex neuron type LDSCORE analysis for schizophrenia with the window size changed from 5kb to 100kb

Supplementary Figure 14: Selection of window size parameters around each transcribed region in the LDSC cell-type analysis.

Results of the LDSC cell-type analysis with different windows size (5kb, 10 kb, 25 kb, 50kb,75 kb and 100 kb) to evaluate the association between schizophrenia and Excitatory neurons and Inhibitory neurons in the cortex. The x-axis represents the windows size added on either side of each gene, while the y-axis represents -log10 p-value associated with LDSC Coefficient.

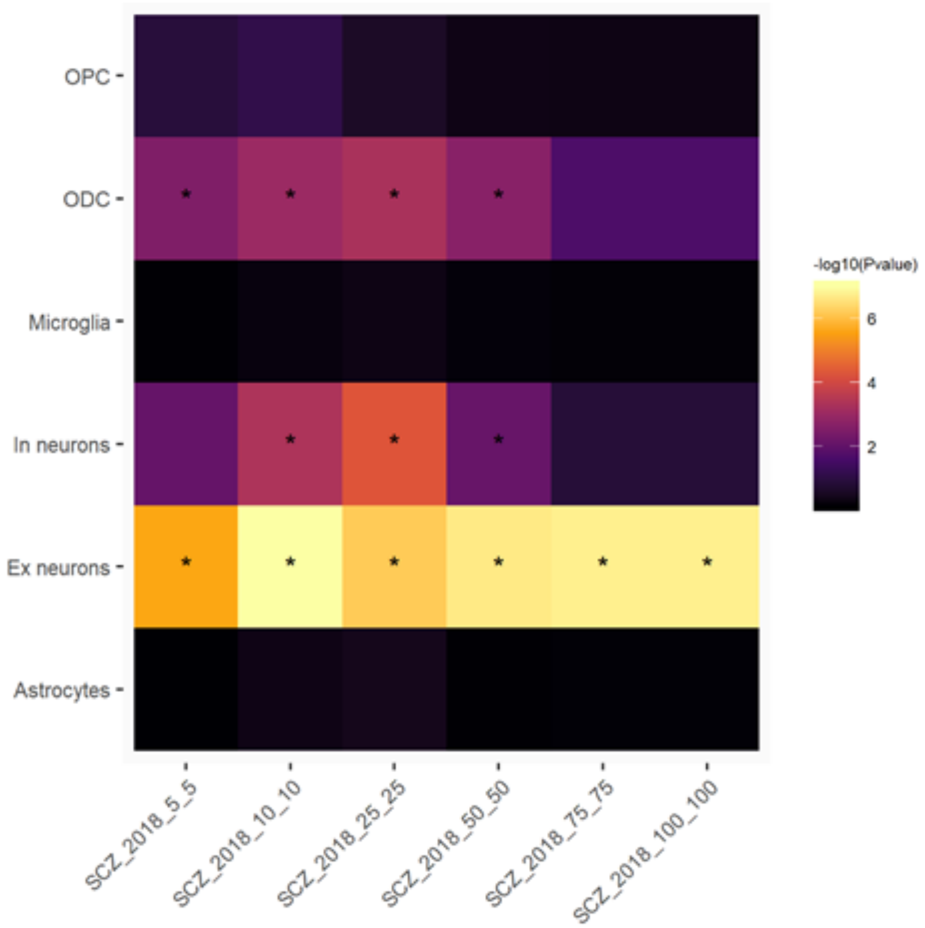

Supplementary Figure 15: Selection of window size parameters around each gene region for the MAGMA gene set based cell-type analysis.

Heatmap showing the $-\log_{10}$ p value of the one-sided positive; two -sample t-test based cell-type association analysis for Schizophrenia for the adult cortex single nuclei cell-types identified in this study at different incremental window sizes (5kb,10kb,25kb,50kb,75kb & 100kb). The window size was benchmarked on the schizophrenia p-value for Excitatory neurons (Ex) and Inhibitory neurons (In). Each cell has been labelled with an * if the -log10 p-value is >= to Bonferroni significance threshold ($-\log_{10}$(0.05/No of Cell-types)). SCZ: Schizophrenia.

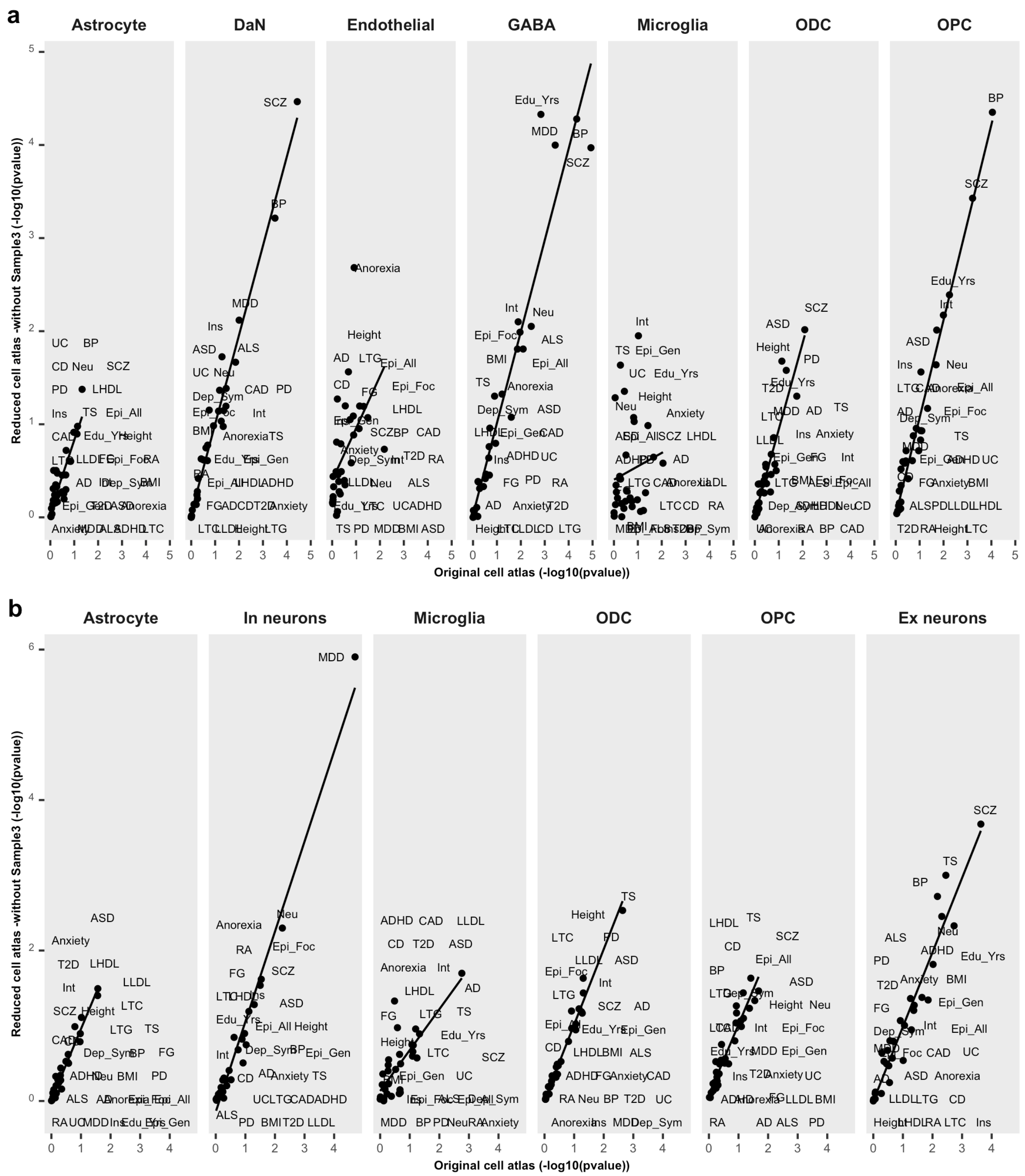

Supplementary Figure 16: Comparison of cell-type enrichment (-log10 pvalues) for different traits between the original cell atlas and the reduced cell atlas (no Sample 3).

The plot compares the cell-type association enrichments (-log10(pvalue)) between the cell atlases with and without the individual with amyloidosis (Sample 3, Supplementary Table1) in the a) SN and b) cortex with the LDSC method in each cell-type. For a specific cell-type, the x-axis and y-axis of each plot represent the -log 10 p-value of enrichment genetic variants associated with human trait by using the original cellular atlases and the reduce cell cellular atlases respectively.

Supplementary Tables

Supplementary Table 1: Single- nuclei RNA sequencing experimental design and sample information

Sample_ID	Source	Brain_Region	Gender	Age	PMI (hours)	Clinical diagnosis	Cause of death	Pathological diagnosis	Consent	Batch	No of lanes
Sample_5_C3	Oxford Brain Bank	Cortex	Male	59	28	Control (No neurological disease)	Myocardial infarction	Amyloid angiopathy, severe.	Yes	Day 1	3
Sample_6_N3	Oxford Brain Bank	Substania nigra	Male	59	28	Control (No neurological disease)	Myocardial infarction	Amyloid angiopathy, severe.	Yes	Day 1	3
Sample_7_N4	Oxford Brain Bank	Substania nigra	Male	70	42	Control (No neurological disease)	out-of-hospital cardiac arrest	Normal aged brain	Yes	Day 1	1
Sample_8_N5	Oxford Brain Bank	Substania nigra	Fema le	56	35	Control (No neurological disease)	aortic dissection due to hypertension	Normal aged brain	Yes	Day 1	1
Sample_9_C1B	Oxford Brain Bank	Cortex	Male	70	43	Control (No neurological disease)	out-of-hospital cardiac arrest	Normal brain for age	Yes	Day 2	3
Sample_10_N1B	Oxford Brain Bank	Substania nigra	Male	70	43	Control (No neurological disease)	out-of-hospital cardiac arrest	Normal brain for age	Yes	Day 2	3
Sample_11_C2B	Oxford Brain Bank	Cortex	Male	55	18	Control (No neurological disease)	Myocardial infarction	Normal brain for age	Yes	Day 2	3
Sample_12_N2B	Oxford Brain Bank	Substantia nigra	Male	55	18	Control (No neurological disease)	Myocardial infarction	Normal brain for age	Yes	Day 2	3
Sample_13_C4B	Oxford Brain Bank	Cortex	Male	70	42	Control (No neurological disease)	out-of-hospital cardiac arrest	Normal aged brain	Yes	Day 2	3
Sample_14_N4B	Oxford Brain Bank	Substantia nigra	Male	70	42	Control (No neurological disease)	out-of-hospital cardiac arrest	Normal aged brain	Yes	Day 2	1
Sample_15_C5B	Oxford Brain Bank	Cortex	Fema le	56	35	Control (No neurological disease)	aortic dissection due to hypertension	Normal aged brain	Yes	Day 2	3
Sample_16_N5B	Oxford Brain Bank	Substantia nigra	Fema le	56	35	Control (No neurological disease)	aortic dissection due to hypertension	Normal aged brain	Yes	Day 2	1

***PMI = Post-mortem interval**

Supplementary Table 2: Gene Ontology pathway analysis of two distinct sub-populations of astrocytes in the Substantia nigra

A: Astrocyte 1 -Top GO Terms: Biological process for Up regulated genes in Astrocytes -1 vs all other cell types

term_ID	description	log10 p-value
GO:0010033	response to organic substance	-17.2815
GO:0032502	developmental process	-16.8729
GO:0007165	signal transduction	-16.5086
GO:0070887	cellular response to chemical stimulus	-16.399
GO:0071310	cellular response to organic substance	-16.2013
GO:0042221	response to chemical	-15.7352
GO:0048856	anatomical structure development	-15.3487
GO:0050896	response to stimulus	-14.3872
GO:0048519	negative regulation of biological process	-13.7595
GO:0050793	regulation of developmental process	-13.5129
GO:0048869	cellular developmental process	-13.3958
GO:0045595	regulation of cell differentiation	-12.2097
GO:0051716	cellular response to stimulus	-12.0696
GO:0051239	regulation of multicellular organismal process	-11.9547
GO:0048523	negative regulation of cellular process	-11.7011
GO:0048522	positive regulation of cellular process	-10.9469
GO:0023051	regulation of signaling	-10.6073
GO:0045597	positive regulation of cell differentiation	-10.475
GO:0048583	regulation of response to stimulus	-10.1331
GO:0048518	positive regulation of biological process	-10.0773
GO:0051094	positive regulation of developmental process	-9.9245
GO:0009719	response to endogenous stimulus	-9.8601

B: Astrocyte -2 :Top GO Terms: Biological process for Up regulated genes in Astrocytes -2 vs all other cell types

term_ID	description	log10 p-value
GO:0050793	regulation of developmental process	-10.2097
GO:0032502	developmental process	-10.0783
GO:0003008	system process	-9.6021
GO:0051239	regulation of multicellular organismal process	-8.6819
GO:0007155	cell adhesion	-8.4895
GO:0022610	biological adhesion	-8.3788
GO:0048856	anatomical structure development	-8.327
GO:0032879	regulation of localization	-7.3915
GO:0065008	regulation of biological quality	-7.2984
GO:0042221	response to chemical	-7.1355
GO:0007165	signal transduction	-6.8539
GO:0032501	multicellular organismal process	-6.5171
GO:0022603	regulation of anatomical structure morphogenesis	-6.433
GO:0033138	positive regulation of peptidyl-serine phosphorylati	-6.3726
GO:0009653	anatomical structure morphogenesis	-6.2857
GO:0051094	positive regulation of developmental process	-6.2366
GO:0051960	regulation of nervous system development	-6.1267
GO:0070887	cellular response to chemical stimulus	-6.0605
GO:0010033	response to organic substance	-5.8861
GO:0048522	positive regulation of cellular process	-5.8761
GO:0071310	cellular response to organic substance	-5.6882
GO:0045595	regulation of cell differentiation	-5.6073
GO:0010562	positive regulation of phosphorus metabolic proces	-5.5258
GO:0002028	regulation of sodium ion transport	-5.5045

Supplementary Table 3: Substantia nigra and cortex cell population proportions

A: Substantia nigra

		Nigra Samples								
		**N1B**	**N2B**	**N3**	**N4**	**N4B**	**N5**	**N5B**	**Total**	**Proportion**
Cell Types	**Astrocytes-2**	0	113	0	0	0	0	0	113	1.9
	**Astrocytes-1**	80	14	4	118	153	40	116	525	8.83
	**DaNeurons**	0	0	7	18	22	2	23	72	1.21
	**GABA neurons**	11	7	0	27	43	11	24	123	2.07
	**Endothelial**	3	5	41	4	3	11	7	74	1.25
	**Microglia**	0	2	242	3	73	5	0	325	5.47
	**ODC-3**	5	956	2	1	4	24	7	999	16.81
	**ODC-2**	380	47	4	147	75	263	127	1043	17.55
	**ODC-1**	129	86	1348	142	101	293	129	2228	37.49
	**OPC**	37	99	72	111	56	25	41	441	7.42
										**5943**

B: Cortex

		Cortex Sample						
		**C1B**	**C2B**	**C3**	**C4B**	**C5B**	**Total**	**Proportion**
Excitatory Neurons	**Ex1**	85	468	197	276	488	**1514**	**14.14**
	**Ex2**	31	377	615	472	639	**2134**	**19.93**
	**Ex3**	6	58	76	95	81	**316**	**2.95**
	**Ex4**	4	207	239	119	332	**901**	**8.42**
	**Ex5a**	2	192	345	148	405	**1092**	**10.2**
	**Ex5b**	11	32	107	42	55	**247**	**2.31**
	**Ex6a**	0	22	48	32	34	**136**	**1.27**
	**Ex6b**	6	26	75	30	42	**179**	**1.67**
	**Ex8**	0	19	11	11	22	**63**	**0.59**
Inhibitory Neurons	**In1a**	7	37	27	24	32	**127**	**1.19**
	**In1b**	8	56	33	38	71	**206**	**1.92**
	**In1c**	17	110	52	36	168	**383**	**3.58**
	**In3**	7	81	35	50	97	**270**	**2.52**
	**In4a**	7	26	12	14	26	**85**	**0.79**
	**In4b**	4	53	53	44	88	**242**	**2.26**
	**In5**	1	17	46	16	44	**124**	**1.16**
	**In6a**	2	43	13	36	50	**144**	**1.35**
	**In6b**	2	180	328	139	277	**926**	**8.65**
	**In8**	2	98	112	23	127	**362**	**3.38**
Non-Neuronal	**Astrocyte**	6	264	20	99	9	**398**	**3.72**
	**Microglia**	9	6	254	88	193	**550**	**5.14**
	**ODC**	145	0	2	0	0	**147**	**1.37**
	**OPC**	19	40	7	27	67	**160**	**1.49**
							10706	

Supplementary Table 4: Source of genome-wide association studies meta-analysis summary statistics used in this study

Trait	Trait Category	Study	Website	Note	
Intelligence	Cognitive Trait	PMID: 28530673	https://ctg.cncr.nl/software/summary_statistics	Summary statistics for intelligence, wave 1 from Suzanne Sniekers et al. 2017	
Education Years	Cognitive Trait	PMID: 27225129	https://www.thessgac.org/data	Summary Statistics for Okbay et al. (2016)	
Ulcerative colitis	Auto-immune disease	PMID: 26192919	https://www.ibdgenetics.org/downloads.html		
Rheumatoid arthritis	Auto-immune disease	PMID: 20453842	ftp://ftp.broadinstitute.org/pub/rheumatoid_arthritis/Stahl_etal_2010NG/		
Crohn's disease	Auto-immune disease	PMID: 26192919	https://www.ibdgenetics.org/downloads.html		
Type 2 Diabetes (Gaulton, 2015)	Metabolic/CardioVascular/Antropor	PMID: 26551672	http://www.diagram-consortium.org/downloads.html	http://www.type2diabetesgenetics.org/home/portalHome	
Tryglycerides concentration	Metabolic/CardioVascular/Antropor	PMID: 20686565,PMID: 24097	http://csg.sph.umich.edu/abecasis/public/lipids2013/jointGwasMc_TG.txt.gz	JOINT ANALYSIS OF METABOCHIP AND GWAS DATA	
Total Cholesterol	Metabolic/CardioVascular/Antropor	PMID: 20686565,PMID: 24097	http://csg.sph.umich.edu/abecasis/public/lipids2013/jointGwasMc_TC.txt.gz	JOINT ANALYSIS OF METABOCHIP AND GWAS DATA	81
LDL concentration	Metabolic/CardioVascular/Antropor	PMID: 20686565,PMID: 24097	http://csg.sph.umich.edu/abecasis/public/lipids2013/jointGwasMc_LDL.txt.gz	JOINT ANALYSIS OF METABOCHIP AND GWAS DATA	
HDL concentration	Metabolic/CardioVascular/Antropor	PMID: 20686565,PMID: 24097	http://csg.sph.umich.edu/abecasis/public/lipids2013/jointGwasMc_HDL.txt.gz	JOINT ANALYSIS OF METABOCHIP AND GWAS DATA	
Height	Metabolic/CardioVascular/Antropor	PMID: 25282103	https://portals.broadinstitute.org/collaboration/giant/images/0/01/GIANT_HEIGHT_Wood_et_al_20	https://portals.broadinstitute.org/collaboration/giant/index.php/GIANT_consortium_data_files	
Fasting glucose	Metabolic/CardioVascular/Antropor	PMID: 20081858	ftp://ftp.sanger.ac.uk/pub/magic/MAGIC_FastingGlucose.txt	https://www.magicinvestigators.org/downloads/	
Coronary artery disease	Metabolic/CardioVascular/Antropor	PMID: 26343387	http://www.cardiogramplusc4d.org/data-downloads/		
Body Mass Index	Metabolic/CardioVascular/Antropor	PMID: 25673413	https://portals.broadinstitute.org/collaboration/giant/images/1/15/SNP_gwas_mc_merge_nogc.tbl.	https://portals.broadinstitute.org/collaboration/giant/index.php/GIANT_consortium_data_files	
Parkinson's disease	Neurological disorder	PMID: 25064009	http://www.pdgene.org/	upon request to International Parkinson Disease Genomics Consortium	
Epilepsy (General)	Neurological disorder	PMID: 25087078	http://www.epigad.org/page/show/gwas_index	http://www.epigad.org/gwas_ilae2014/ILAE_GGE_5.8.14.txt.gz	
Epilepsy (Focal)	Neurological disorder	PMID: 25087078	http://www.epigad.org/page/show/gwas_index	http://www.epigad.org/gwas_ilae2014/ILAE_Focal_5.8.14.txt.gz	
Epilepsy (All forms)	Neurological disorder	PMID: 25087078	http://www.epigad.org/page/show/gwas_index	http://www.epigad.org/gwas_ilae2014/ILAE_All_Epi_11.8.14.txt.gz	
Amyotrophic Lateral Sclerosis	Neurological disorder	PMID: 27455348	http://databrowser.projectmine.com/		
Alzheimer's disease (Jansenetal)	Neurological disorder	PMID: 30617256	https://ctg.cncr.nl/software/summary_statistics	https://ctg.cncr.nl/documents/p1651/AD_sumstats_Jansenetal.txt.gz	
Tourette Syndrome	Neuro-psychatric disorder	PMID: 30818990	https://www.med.unc.edu/pgc/results-and-downloads/ts/		
Schizophrenia (2018)	Neuro-psychatric disorder	PMID: 29483656	https://walters.psycm.cf.ac.uk/		
Neuroticism	Neuro-psychatric disorder	PMID: 27089181	http://ssgac.org/documents/Neuroticism_Full.txt.gz		
Major Depression	Neuro-psychatric disorder	PMID: 29700475	https://www.med.unc.edu/pgc/results-and-downloads/mdd/		
Insomnia	Neuro-psychatric disorder	PMID: 28604731	https://ctg.cncr.nl/documents/p1651/Hammerschlag_NatGenet2017_insomnia_sumstats-full_090617.txt.gz		
Depressive Symtoms	Neuro-psychatric disorder	PMID: 27089181	http://ssgac.org/documents/DS_Full.txt.gz		
Bipolar	Neuro-psychatric disorder	PMID: 21926972	https://www.med.unc.edu/pgc/results-and-downloads/bip/		
Autism disorder	Neuro-psychatric disorder	PMID: 30804558	https://www.med.unc.edu/pgc/results-and-downloads/asd/		
Anxiety	Neuro-psychatric disorder	PMID: 26754954	https://www.med.unc.edu/pgc/results-and-downloads/angst/		
Anorexia	Neuro-psychatric disorder	PMID: 28494655	https://www.med.unc.edu/pgc/results-and-downloads/ed/		

Supplementary Table 5 : Risk association p-values for 30 brain and non-brain-related traits with cell-types from the Substantia nigra (SN) using LDSC & MAGMA

Trait	Category	Cell	Pvalue_LDSC	Qvalue_LDSC	Pvalue_MAGMA	Qvalue_MAGMA	Methods	Region
ADHD	Psychatric disorder	Astrocyte	8.92E-01	1.00E+00	4.26E-01	1.00E+00	NONE	Substantia nigra
ADHD	Psychatric disorder	DaN	5.76E-01	1.00E+00	1.92E-02	1.34E-01	NONE	Substantia nigra
ADHD	Psychatric disorder	Endothelial	8.61E-01	1.00E+00	9.68E-01	1.00E+00	NONE	Substantia nigra
ADHD	Psychatric disorder	GABA	2.07E-01	1.00E+00	3.57E-03	2.50E-02	MAGMA**	Substantia nigra
ADHD	Psychatric disorder	Microglia	5.26E-01	1.00E+00	6.13E-01	1.00E+00	NONE	Substantia nigra
ADHD	Psychatric disorder	ODC	7.29E-01	1.00E+00	9.14E-01	1.00E+00	NONE	Substantia nigra
ADHD	Psychatric disorder	OPC	2.01E-01	1.00E+00	3.01E-02	2.11E-01	NONE	Substantia nigra
Alzheimer's disease(Jansenetal)	Neurological disorder	Astrocyte	3.21E-01	1.00E+00	3.06E-02	2.14E-01	NONE	Substantia nigra
Alzheimer's disease(Jansenetal)	Neurological disorder	DaN	7.66E-01	1.00E+00	3.56E-01	1.00E+00	NONE	Substantia nigra
Alzheimer's disease(Jansenetal)	Neurological disorder	Endothelial	6.02E-01	1.00E+00	2.36E-01	1.00E+00	NONE	Substantia nigra
Alzheimer's disease(Jansenetal)	Neurological disorder	GABA	7.00E-01	1.00E+00	3.84E-01	1.00E+00	NONE	Substantia nigra
Alzheimer's disease(Jansenetal)	Neurological disorder	Microglia	8.88E-03	6.21E-02	1.40E-04	9.81E-04	MAGMA** & LDSC*	Substantia nigra
Alzheimer's disease(Jansenetal)	Neurological disorder	ODC	1.45E-01	1.00E+00	4.85E-01	1.00E+00	NONE	Substantia nigra
Alzheimer's disease(Jansenetal)	Neurological disorder	OPC	5.02E-01	1.00E+00	1.22E-01	8.56E-01	NONE	Substantia nigra
Amyotrophic Lateral Sclerosis	Neurological disorder	Astrocyte	8.54E-01	1.00E+00	6.34E-01	1.00E+00	NONE	Substantia nigra
Amyotrophic Lateral Sclerosis	Neurological disorder	DaN	1.40E-02	9.78E-02	7.30E-02	5.11E-01	NONE	Substantia nigra
Amyotrophic Lateral Sclerosis	Neurological disorder	Endothelial	3.06E-01	1.00E+00	4.09E-02	2.86E-01	NONE	Substantia nigra
Amyotrophic Lateral Sclerosis	Neurological disorder	GABA	1.08E-02	7.57E-02	5.18E-01	1.00E+00	NONE	Substantia nigra
Amyotrophic Lateral Sclerosis	Neurological disorder	Microglia	8.58E-01	1.00E+00	4.26E-01	1.00E+00	NONE	Substantia nigra
Amyotrophic Lateral Sclerosis	Neurological disorder	ODC	6.12E-01	1.00E+00	7.67E-01	1.00E+00	NONE	Substantia nigra
Amyotrophic Lateral Sclerosis	Neurological disorder	OPC	7.59E-01	1.00E+00	5.29E-01	1.00E+00	NONE	Substantia nigra
Anorexia	Psychatric disorder	Astrocyte	2.67E-01	1.00E+00	6.40E-01	1.00E+00	NONE	Substantia nigra
Anorexia	Psychatric disorder	DaN	1.17E-01	8.18E-01	8.31E-02	5.82E-01	NONE	Substantia nigra
Anorexia	Psychatric disorder	Endothelial	1.20E-01	8.41E-01	7.86E-01	1.00E+00	NONE	Substantia nigra
Anorexia	Psychatric disorder	GABA	2.11E-01	1.00E+00	2.19E-01	1.00E+00	NONE	Substantia nigra
Anorexia	Psychatric disorder	Microglia	4.78E-02	3.34E-01	5.53E-01	1.00E+00	NONE	Substantia nigra
Anorexia	Psychatric disorder	ODC	8.23E-01	1.00E+00	7.81E-01	1.00E+00	NONE	Substantia nigra
Anorexia	Psychatric disorder	OPC	8.83E-02	6.18E-01	4.79E-02	3.35E-01	NONE	Substantia nigra
Anxiety	Psychatric disorder	Astrocyte	9.40E-01	1.00E+00	6.61E-01	1.00E+00	NONE	Substantia nigra
Anxiety	Psychatric disorder	DaN	5.45E-01	1.00E+00	1.59E-01	1.00E+00	NONE	Substantia nigra
Anxiety	Psychatric disorder	Endothelial	4.33E-01	1.00E+00	2.44E-01	1.00E+00	NONE	Substantia nigra
Anxiety	Psychatric disorder	GABA	9.61E-01	1.00E+00	4.49E-01	1.00E+00	NONE	Substantia nigra
Anxiety	Psychatric disorder	Microglia	3.77E-02	2.64E-01	7.28E-01	1.00E+00	NONE	Substantia nigra
Anxiety	Psychatric disorder	ODC	2.10E-01	1.00E+00	5.97E-02	4.18E-01	NONE	Substantia nigra
Anxiety	Psychatric disorder	OPC	7.08E-01	1.00E+00	3.69E-01	1.00E+00	NONE	Substantia nigra
Autism disorder	Psychatric disorder	Astrocyte	7.47E-01	1.00E+00	2.32E-01	1.00E+00	NONE	Substantia nigra
Autism disorder	Psychatric disorder	DaN	6.51E-02	4.56E-01	5.80E-03	4.06E-02	MAGMA**	Substantia nigra
Autism disorder	Psychatric disorder	Endothelial	9.19E-01	1.00E+00	8.35E-01	1.00E+00	NONE	Substantia nigra
Autism disorder	Psychatric disorder	GABA	2.52E-02	1.76E-01	8.66E-03	6.06E-02	NONE	Substantia nigra
Autism disorder	Psychatric disorder	Microglia	3.13E-01	1.00E+00	1.62E-02	1.14E-01	NONE	Substantia nigra
Autism disorder	Psychatric disorder	ODC	7.39E-02	5.17E-01	1.27E-01	8.91E-01	NONE	Substantia nigra
Autism disorder	Psychatric disorder	OPC	1.93E-02	1.35E-01	3.96E-03	2.77E-02	MAGMA** & LDSC*	Substantia nigra
Bipolar	Psychatric disorder	Astrocyte	4.68E-02	3.28E-01	1.21E-01	8.46E-01	NONE	Substantia nigra
Bipolar	Psychatric disorder	DaN	3.14E-04	2.20E-03	2.59E-06	1.81E-05	Both**	Substantia nigra
Bipolar	Psychatric disorder	Endothelial	1.58E-01	1.00E+00	2.54E-01	1.00E+00	NONE	Substantia nigra

Bipolar	Psychatric disorder	GABA	4.60E-05	3.22E-04	4.63E-03	3.24E-02	Both**	Substantia nigra
Bipolar	Psychatric disorder	Microglia	4.82E-01	1.00E+00	3.63E-01	1.00E+00	NONE	Substantia nigra
Bipolar	Psychatric disorder	ODC	8.23E-01	1.00E+00	4.93E-02	3.45E-01	NONE	Substantia nigra
Bipolar	Psychatric disorder	OPC	9.05E-05	6.34E-04	4.08E-03	2.86E-02	Both**	Substantia nigra
BMI	Metabolic/Cardio/Anthropometric	Astrocyte	5.39E-01	1.00E+00	3.20E-01	1.00E+00	NONE	Substantia nigra
BMI	Metabolic/Cardio/Anthropometric	DaN	3.83E-01	1.00E+00	2.06E-03	1.44E-02	MAGMA**	Substantia nigra
BMI	Metabolic/Cardio/Anthropometric	Endothelial	9.60E-01	1.00E+00	5.97E-01	1.00E+00	NONE	Substantia nigra
BMI	Metabolic/Cardio/Anthropometric	GABA	6.11E-02	4.27E-01	4.80E-04	3.36E-03	MAGMA**	Substantia nigra
BMI	Metabolic/Cardio/Anthropometric	Microglia	9.87E-01	1.00E+00	5.51E-01	1.00E+00	NONE	Substantia nigra
BMI	Metabolic/Cardio/Anthropometric	ODC	3.23E-01	1.00E+00	6.26E-01	1.00E+00	NONE	Substantia nigra
BMI	Metabolic/Cardio/Anthropometric	OPC	3.01E-01	1.00E+00	4.49E-02	3.14E-01	NONE	Substantia nigra
Coronary Artery Disease	Metabolic/Cardio/Anthropometric	Astrocyte	5.99E-01	1.00E+00	1.05E-02	7.36E-02	NONE	Substantia nigra
Coronary Artery Disease	Metabolic/Cardio/Anthropometric	DaN	3.53E-02	2.47E-01	8.47E-01	1.00E+00	NONE	Substantia nigra
Coronary Artery Disease	Metabolic/Cardio/Anthropometric	Endothelial	6.56E-03	4.59E-02	3.35E-07	2.35E-06	Both**	Substantia nigra
Coronary Artery Disease	Metabolic/Cardio/Anthropometric	GABA	2.20E-01	1.00E+00	4.50E-01	1.00E+00	NONE	Substantia nigra
Coronary Artery Disease	Metabolic/Cardio/Anthropometric	Microglia	2.11E-01	1.00E+00	2.46E-02	1.72E-01	NONE	Substantia nigra
Coronary Artery Disease	Metabolic/Cardio/Anthropometric	ODC	7.71E-01	1.00E+00	3.60E-02	2.52E-01	NONE	Substantia nigra
Coronary Artery Disease	Metabolic/Cardio/Anthropometric	OPC	1.39E-01	9.70E-01	3.39E-01	1.00E+00	NONE	Substantia nigra
Crohn Disease	Immune	Astrocyte	7.57E-01	1.00E+00	3.01E-02	2.11E-01	NONE	Substantia nigra
Crohn Disease	Immune	DaN	9.02E-01	1.00E+00	6.56E-01	1.00E+00	NONE	Substantia nigra
Crohn Disease	Immune	Endothelial	2.81E-01	1.00E+00	5.31E-02	3.72E-01	NONE	Substantia nigra
Crohn Disease	Immune	GABA	9.10E-01	1.00E+00	9.34E-01	1.00E+00	NONE	Substantia nigra
Crohn Disease	Immune	Microglia	1.78E-01	1.00E+00	1.19E-05	8.30E-05	MAGMA**	Substantia nigra
Crohn Disease	Immune	ODC	6.79E-01	1.00E+00	4.03E-01	1.00E+00	NONE	Substantia nigra
Crohn Disease	Immune	OPC	6.02E-01	1.00E+00	7.57E-01	1.00E+00	NONE	Substantia nigra
Depressive Symptoms	Psychatric disorder	Astrocyte	2.72E-01	1.00E+00	9.35E-02	6.54E-01	NONE	Substantia nigra
Depressive Symptoms	Psychatric disorder	DaN	7.18E-02	5.02E-01	3.58E-02	2.51E-01	NONE	Substantia nigra
Depressive Symptoms	Psychatric disorder	Endothelial	6.84E-01	1.00E+00	2.62E-01	1.00E+00	NONE	Substantia nigra
Depressive Symptoms	Psychatric disorder	GABA	1.99E-01	1.00E+00	2.06E-01	1.00E+00	NONE	Substantia nigra
Depressive Symptoms	Psychatric disorder	Microglia	5.82E-02	4.07E-01	7.55E-03	5.28E-02	NONE	Substantia nigra
Depressive Symptoms	Psychatric disorder	ODC	8.01E-01	1.00E+00	7.58E-01	1.00E+00	NONE	Substantia nigra
Depressive Symptoms	Psychatric disorder	OPC	9.08E-02	6.36E-01	1.84E-02	1.29E-01	NONE	Substantia nigra
Educations Years	Cog.	Astrocyte	2.26E-01	1.00E+00	8.73E-02	6.11E-01	NONE	Substantia nigra
Educations Years	Cog.	DaN	2.10E-01	1.00E+00	1.80E-01	1.00E+00	NONE	Substantia nigra
Educations Years	Cog.	Endothelial	9.09E-01	1.00E+00	6.39E-01	1.00E+00	NONE	Substantia nigra
Educations Years	Cog.	GABA	1.45E-03	1.02E-02	3.15E-03	2.20E-02	Both**	Substantia nigra
Educations Years	Cog.	Microglia	1.50E-01	1.00E+00	3.14E-01	1.00E+00	NONE	Substantia nigra
Educations Years	Cog.	ODC	2.06E-01	1.00E+00	1.36E-02	9.54E-02	NONE	Substantia nigra
Educations Years	Cog.	OPC	5.65E-03	3.96E-02	1.14E-02	7.96E-02	LDSC** & MAGMA*	Substantia nigra
Epilepsy (All forms)	Neurological disorder	Astrocyte	1.41E-01	9.86E-01	1.10E-02	7.71E-02	NONE	Substantia nigra
Epilepsy (All forms)	Neurological disorder	DaN	5.06E-01	1.00E+00	6.94E-01	1.00E+00	NONE	Substantia nigra
Epilepsy (All forms)	Neurological disorder	Endothelial	4.82E-02	3.37E-01	2.23E-01	1.00E+00	NONE	Substantia nigra
Epilepsy (All forms)	Neurological disorder	GABA	8.01E-03	5.61E-02	4.72E-01	1.00E+00	NONE	Substantia nigra
Epilepsy (All forms)	Neurological disorder	Microglia	5.78E-01	1.00E+00	1.63E-01	1.00E+00	NONE	Substantia nigra
Epilepsy (All forms)	Neurological disorder	ODC	2.21E-01	1.00E+00	4.04E-01	1.00E+00	NONE	Substantia nigra
Epilepsy (All forms)	Neurological disorder	OPC	4.67E-02	3.27E-01	6.85E-02	4.80E-01	NONE	Substantia nigra
Epilepsy (Focal)	Neurological disorder	Astrocyte	2.24E-01	1.00E+00	1.88E-01	1.00E+00	NONE	Substantia nigra
Epilepsy (Focal)	Neurological disorder	DaN	1.77E-01	1.00E+00	6.38E-01	1.00E+00	NONE	Substantia nigra
Epilepsy (Focal)	Neurological disorder	Endothelial	3.21E-02	2.25E-01	6.03E-01	1.00E+00	NONE	Substantia nigra

Epilepsy (Focal)	Neurological disorder	GABA	1.39E-02	9.70E-02	7.56E-01	1.00E+00	NONE	Substantia nigra
Epilepsy (Focal)	Neurological disorder	Microglia	4.70E-01	1.00E+00	2.93E-01	1.00E+00	NONE	Substantia nigra
Epilepsy (Focal)	Neurological disorder	ODC	4.01E-01	1.00E+00	5.46E-01	1.00E+00	NONE	Substantia nigra
Epilepsy (Focal)	Neurological disorder	OPC	8.09E-02	5.66E-01	1.51E-01	1.00E+00	NONE	Substantia nigra
Epilepsy (General)	Neurological disorder	Astrocyte	5.92E-01	1.00E+00	1.66E-01	1.00E+00	NONE	Substantia nigra
Epilepsy (General)	Neurological disorder	DaN	2.42E-01	1.00E+00	6.67E-02	4.67E-01	NONE	Substantia nigra
Epilepsy (General)	Neurological disorder	Endothelial	1.77E-01	1.00E+00	6.98E-01	1.00E+00	NONE	Substantia nigra
Epilepsy (General)	Neurological disorder	GABA	1.14E-01	7.95E-01	3.09E-02	2.16E-01	NONE	Substantia nigra
Epilepsy (General)	Neurological disorder	Microglia	8.90E-01	1.00E+00	7.19E-01	1.00E+00	NONE	Substantia nigra
Epilepsy (General)	Neurological disorder	ODC	1.28E-01	8.96E-01	6.55E-01	1.00E+00	NONE	Substantia nigra
Epilepsy (General)	Neurological disorder	OPC	3.78E-01	1.00E+00	3.52E-01	1.00E+00	NONE	Substantia nigra
Fasting Glucose	Metabolic/Cardio/Anthropometric	Astrocyte	5.65E-01	1.00E+00	1.84E-01	1.00E+00	NONE	Substantia nigra
Fasting Glucose	Metabolic/Cardio/Anthropometric	DaN	6.18E-01	1.00E+00	9.71E-01	1.00E+00	NONE	Substantia nigra
Fasting Glucose	Metabolic/Cardio/Anthropometric	Endothelial	7.06E-02	4.94E-01	2.15E-01	1.00E+00	NONE	Substantia nigra
Fasting Glucose	Metabolic/Cardio/Anthropometric	GABA	3.66E-01	1.00E+00	9.06E-01	1.00E+00	NONE	Substantia nigra
Fasting Glucose	Metabolic/Cardio/Anthropometric	Microglia	7.07E-01	1.00E+00	2.60E-01	1.00E+00	NONE	Substantia nigra
Fasting Glucose	Metabolic/Cardio/Anthropometric	ODC	6.31E-01	1.00E+00	6.11E-03	4.28E-02	MAGMA**	Substantia nigra
Fasting Glucose	Metabolic/Cardio/Anthropometric	OPC	5.97E-01	1.00E+00	6.16E-01	1.00E+00	NONE	Substantia nigra
Height (2014)	Metabolic/Cardio/Anthropometric	Astrocyte	6.61E-01	1.00E+00	2.91E-05	2.03E-04	MAGMA**	Substantia nigra
Height (2014)	Metabolic/Cardio/Anthropometric	DaN	9.98E-01	1.00E+00	9.88E-01	1.00E+00	NONE	Substantia nigra
Height (2014)	Metabolic/Cardio/Anthropometric	Endothelial	2.08E-01	1.00E+00	1.86E-03	1.30E-02	MAGMA**	Substantia nigra
Height (2014)	Metabolic/Cardio/Anthropometric	GABA	1.00E+00	1.00E+00	9.99E-01	1.00E+00	NONE	Substantia nigra
Height (2014)	Metabolic/Cardio/Anthropometric	Microglia	5.74E-01	1.00E+00	6.93E-02	4.85E-01	NONE	Substantia nigra
Height (2014)	Metabolic/Cardio/Anthropometric	ODC	7.15E-01	1.00E+00	1.06E-02	7.45E-02	NONE	Substantia nigra
Height (2014)	Metabolic/Cardio/Anthropometric	OPC	8.13E-01	1.00E+00	4.95E-01	1.00E+00	NONE	Substantia nigra
Insomnia	Psychatric disorder	Astrocyte	5.09E-01	1.00E+00	3.24E-01	1.00E+00	NONE	Substantia nigra
Insomnia	Psychatric disorder	DaN	5.17E-02	3.62E-01	5.67E-02	3.97E-01	NONE	Substantia nigra
Insomnia	Psychatric disorder	Endothelial	6.48E-01	1.00E+00	6.76E-01	1.00E+00	NONE	Substantia nigra
Insomnia	Psychatric disorder	GABA	2.87E-01	1.00E+00	9.16E-02	6.42E-01	NONE	Substantia nigra
Insomnia	Psychatric disorder	Microglia	3.83E-01	1.00E+00	4.26E-01	1.00E+00	NONE	Substantia nigra
Insomnia	Psychatric disorder	ODC	4.05E-01	1.00E+00	1.39E-01	9.76E-01	NONE	Substantia nigra
Insomnia	Psychatric disorder	OPC	3.68E-01	1.00E+00	2.98E-02	2.09E-01	NONE	Substantia nigra
Intelligence	Cog.	Astrocyte	3.62E-01	1.00E+00	3.02E-01	1.00E+00	NONE	Substantia nigra
Intelligence	Cog.	DaN	5.55E-02	3.89E-01	5.59E-02	3.91E-01	NONE	Substantia nigra
Intelligence	Cog.	Endothelial	5.72E-01	1.00E+00	5.14E-01	1.00E+00	NONE	Substantia nigra
Intelligence	Cog.	GABA	1.29E-02	9.04E-02	4.28E-02	2.99E-01	NONE	Substantia nigra
Intelligence	Cog.	Microglia	9.47E-02	6.63E-01	8.77E-02	6.14E-01	NONE	Substantia nigra
Intelligence	Cog.	ODC	4.86E-01	1.00E+00	5.16E-01	1.00E+00	NONE	Substantia nigra
Intelligence	Cog.	OPC	1.01E-02	7.10E-02	1.80E-03	1.26E-02	MAGMA** & LDSC*	Substantia nigra
Lipid HDL	Metabolic/Cardio/Anthropometric	Astrocyte	1.04E-01	7.31E-01	8.20E-03	5.74E-02	NONE	Substantia nigra
Lipid HDL	Metabolic/Cardio/Anthropometric	DaN	5.53E-01	1.00E+00	4.95E-01	1.00E+00	NONE	Substantia nigra
Lipid HDL	Metabolic/Cardio/Anthropometric	Endothelial	7.45E-02	5.21E-01	3.16E-03	2.21E-02	MAGMA**	Substantia nigra
Lipid HDL	Metabolic/Cardio/Anthropometric	GABA	6.20E-01	1.00E+00	6.89E-01	1.00E+00	NONE	Substantia nigra
Lipid HDL	Metabolic/Cardio/Anthropometric	Microglia	2.20E-02	1.54E-01	6.65E-03	4.66E-02	MAGMA** & LDSC*	Substantia nigra
Lipid HDL	Metabolic/Cardio/Anthropometric	ODC	8.46E-01	1.00E+00	1.41E-03	9.90E-03	MAGMA**	Substantia nigra
Lipid HDL	Metabolic/Cardio/Anthropometric	OPC	6.09E-01	1.00E+00	2.45E-01	1.00E+00	NONE	Substantia nigra
Lipid LDL	Metabolic/Cardio/Anthropometric	Astrocyte	5.36E-01	1.00E+00	2.18E-01	1.00E+00	NONE	Substantia nigra
Lipid LDL	Metabolic/Cardio/Anthropometric	DaN	9.97E-01	1.00E+00	9.74E-01	1.00E+00	NONE	Substantia nigra
Lipid LDL	Metabolic/Cardio/Anthropometric	Endothelial	5.27E-01	1.00E+00	1.98E-01	1.00E+00	NONE	Substantia nigra

Lipid LDL	Metabolic/Cardio/Anthropometric	GABA	6.32E-01	1.00E+00	9.90E-01	1.00E+00	NONE	Substantia nigra
Lipid LDL	Metabolic/Cardio/Anthropometric	Microglia	3.28E-01	1.00E+00	5.24E-03	3.67E-02	MAGMA**	Substantia nigra
Lipid LDL	Metabolic/Cardio/Anthropometric	ODC	3.42E-01	1.00E+00	1.78E-01	1.00E+00	NONE	Substantia nigra
Lipid LDL	Metabolic/Cardio/Anthropometric	OPC	6.21E-01	1.00E+00	9.36E-01	1.00E+00	NONE	Substantia nigra
Lipid TC	Metabolic/Cardio/Anthropometric	Astrocyte	7.94E-01	1.00E+00	6.04E-02	4.23E-01	NONE	Substantia nigra
Lipid TC	Metabolic/Cardio/Anthropometric	DaN	1.00E+00	1.00E+00	9.59E-01	1.00E+00	NONE	Substantia nigra
Lipid TC	Metabolic/Cardio/Anthropometric	Endothelial	4.57E-01	1.00E+00	7.34E-02	5.14E-01	NONE	Substantia nigra
Lipid TC	Metabolic/Cardio/Anthropometric	GABA	9.20E-01	1.00E+00	9.88E-01	1.00E+00	NONE	Substantia nigra
Lipid TC	Metabolic/Cardio/Anthropometric	Microglia	1.73E-01	1.00E+00	1.63E-04	1.14E-03	MAGMA**	Substantia nigra
Lipid TC	Metabolic/Cardio/Anthropometric	ODC	3.52E-01	1.00E+00	3.23E-01	1.00E+00	NONE	Substantia nigra
Lipid TC	Metabolic/Cardio/Anthropometric	OPC	6.95E-01	1.00E+00	7.86E-01	1.00E+00	NONE	Substantia nigra
Lipid TG	Metabolic/Cardio/Anthropometric	Astrocyte	3.17E-01	1.00E+00	1.15E-01	8.08E-01	NONE	Substantia nigra
Lipid TG	Metabolic/Cardio/Anthropometric	DaN	9.74E-01	1.00E+00	9.32E-01	1.00E+00	NONE	Substantia nigra
Lipid TG	Metabolic/Cardio/Anthropometric	Endothelial	1.30E-01	9.12E-01	4.47E-01	1.00E+00	NONE	Substantia nigra
Lipid TG	Metabolic/Cardio/Anthropometric	GABA	8.81E-01	1.00E+00	9.73E-01	1.00E+00	NONE	Substantia nigra
Lipid TG	Metabolic/Cardio/Anthropometric	Microglia	2.92E-01	1.00E+00	2.03E-03	1.42E-02	MAGMA**	Substantia nigra
Lipid TG	Metabolic/Cardio/Anthropometric	ODC	5.30E-01	1.00E+00	4.91E-02	3.44E-01	NONE	Substantia nigra
Lipid TG	Metabolic/Cardio/Anthropometric	OPC	1.84E-01	1.00E+00	3.36E-01	1.00E+00	NONE	Substantia nigra
Major Depression	Psychatric disorder	Astrocyte	9.47E-01	1.00E+00	8.77E-01	1.00E+00	NONE	Substantia nigra
Major Depression	Psychatric disorder	DaN	9.92E-03	6.94E-02	2.29E-02	1.60E-01	NONE	Substantia nigra
Major Depression	Psychatric disorder	Endothelial	9.39E-01	1.00E+00	8.84E-01	1.00E+00	NONE	Substantia nigra
Major Depression	Psychatric disorder	GABA	3.66E-04	2.56E-03	1.82E-03	1.27E-02	Both**	Substantia nigra
Major Depression	Psychatric disorder	Microglia	9.71E-01	1.00E+00	9.49E-01	1.00E+00	NONE	Substantia nigra
Major Depression	Psychatric disorder	ODC	3.62E-01	1.00E+00	1.91E-01	1.00E+00	NONE	Substantia nigra
Major Depression	Psychatric disorder	OPC	1.10E-01	7.68E-01	1.99E-02	1.40E-01	NONE	Substantia nigra
Neuroticism	Psychatric disorder	Astrocyte	2.18E-01	1.00E+00	1.82E-02	1.27E-01	NONE	Substantia nigra
Neuroticism	Psychatric disorder	DaN	3.46E-02	2.42E-01	1.63E-02	1.14E-01	NONE	Substantia nigra
Neuroticism	Psychatric disorder	Endothelial	2.88E-01	1.00E+00	5.29E-01	1.00E+00	NONE	Substantia nigra
Neuroticism	Psychatric disorder	GABA	3.67E-03	2.57E-02	2.57E-02	1.80E-01	LDSC** & MAGMA*	Substantia nigra
Neuroticism	Psychatric disorder	Microglia	1.42E-01	9.96E-01	4.23E-01	1.00E+00	NONE	Substantia nigra
Neuroticism	Psychatric disorder	ODC	6.66E-01	1.00E+00	5.25E-01	1.00E+00	NONE	Substantia nigra
Neuroticism	Psychatric disorder	OPC	2.05E-02	1.44E-01	2.68E-04	1.88E-03	MAGMA** & LDSC*	Substantia nigra
Parkinson disease	Neurological disorder	Astrocyte	5.74E-01	1.00E+00	1.74E-01	1.00E+00	NONE	Substantia nigra
Parkinson disease	Neurological disorder	DaN	4.38E-02	3.07E-01	6.63E-04	4.64E-03	MAGMA** & LDSC*	Substantia nigra
Parkinson disease	Neurological disorder	Endothelial	5.99E-01	1.00E+00	2.28E-01	1.00E+00	NONE	Substantia nigra
Parkinson disease	Neurological disorder	GABA	5.87E-01	1.00E+00	1.04E-02	7.25E-02	NONE	Substantia nigra
Parkinson disease	Neurological disorder	Microglia	7.86E-01	1.00E+00	4.77E-01	1.00E+00	NONE	Substantia nigra
Parkinson disease	Neurological disorder	ODC	4.85E-02	3.39E-01	5.12E-03	3.58E-02	MAGMA** & LDSC*	Substantia nigra
Parkinson disease	Neurological disorder	OPC	7.03E-01	1.00E+00	5.22E-03	3.66E-02	MAGMA**	Substantia nigra
Rheumatoid Arthritis	Immune	Astrocyte	5.74E-01	1.00E+00	2.92E-01	1.00E+00	NONE	Substantia nigra
Rheumatoid Arthritis	Immune	DaN	3.04E-01	1.00E+00	8.64E-01	1.00E+00	NONE	Substantia nigra
Rheumatoid Arthritis	Immune	Endothelial	9.10E-01	1.00E+00	3.21E-01	1.00E+00	NONE	Substantia nigra
Rheumatoid Arthritis	Immune	GABA	5.29E-01	1.00E+00	8.51E-01	1.00E+00	NONE	Substantia nigra
Rheumatoid Arthritis	Immune	Microglia	1.05E-01	7.33E-01	6.21E-01	1.00E+00	NONE	Substantia nigra
Rheumatoid Arthritis	Immune	ODC	9.22E-01	1.00E+00	5.20E-01	1.00E+00	NONE	Substantia nigra
Rheumatoid Arthritis	Immune	OPC	8.26E-01	1.00E+00	2.18E-01	1.00E+00	NONE	Substantia nigra
Schizophrenia (2018)	Psychatric disorder	Astrocyte	7.25E-02	5.08E-01	6.44E-02	4.51E-01	NONE	Substantia nigra
Schizophrenia (2018)	Psychatric disorder	DaN	3.66E-05	2.57E-04	2.14E-07	1.50E-06	Both**	Substantia nigra
Schizophrenia (2018)	Psychatric disorder	Endothelial	1.26E-01	8.81E-01	4.26E-02	2.98E-01	NONE	Substantia nigra

Schizophrenia (2018)	Psychatric disorder	GABA	1.17E-05	8.19E-05	1.03E-07	7.20E-07	Both**	Substantia nigra
Schizophrenia (2018)	Psychatric disorder	Microglia	8.13E-01	1.00E+00	1.69E-01	1.00E+00	NONE	Substantia nigra
Schizophrenia (2018)	Psychatric disorder	ODC	8.15E-03	5.70E-02	1.95E-06	1.36E-05	MAGMA** & LDSC*	Substantia nigra
Schizophrenia (2018)	Psychatric disorder	OPC	6.10E-04	4.27E-03	1.88E-05	1.32E-04	Both**	Substantia nigra
Tourette Syndrome	Psychatric disorder	Astrocyte	7.62E-02	5.33E-01	1.98E-02	1.39E-01	NONE	Substantia nigra
Tourette Syndrome	Psychatric disorder	DaN	4.60E-02	3.22E-01	7.58E-02	5.31E-01	NONE	Substantia nigra
Tourette Syndrome	Psychatric disorder	Endothelial	6.55E-01	1.00E+00	6.48E-01	1.00E+00	NONE	Substantia nigra
Tourette Syndrome	Psychatric disorder	GABA	1.25E-01	8.77E-01	4.63E-02	3.24E-01	NONE	Substantia nigra
Tourette Syndrome	Psychatric disorder	Microglia	5.34E-01	1.00E+00	3.24E-01	1.00E+00	NONE	Substantia nigra
Tourette Syndrome	Psychatric disorder	ODC	1.76E-02	1.23E-01	9.11E-03	6.38E-02	NONE	Substantia nigra
Tourette Syndrome	Psychatric disorder	OPC	9.15E-02	6.41E-01	1.38E-01	9.64E-01	NONE	Substantia nigra
Type 2 diabetes (Gaulton)	Metabolic/Cardio/Anthropometric	Astrocyte	5.08E-01	1.00E+00	3.02E-03	2.11E-02	MAGMA**	Substantia nigra
Type 2 diabetes (Gaulton)	Metabolic/Cardio/Anthropometric	DaN	9.65E-01	1.00E+00	6.38E-01	1.00E+00	NONE	Substantia nigra
Type 2 diabetes (Gaulton)	Metabolic/Cardio/Anthropometric	Endothelial	4.15E-01	1.00E+00	1.63E-01	1.00E+00	NONE	Substantia nigra
Type 2 diabetes (Gaulton)	Metabolic/Cardio/Anthropometric	GABA	8.84E-01	1.00E+00	9.03E-01	1.00E+00	NONE	Substantia nigra
Type 2 diabetes (Gaulton)	Metabolic/Cardio/Anthropometric	Microglia	7.70E-02	5.39E-01	6.09E-02	4.26E-01	NONE	Substantia nigra
Type 2 diabetes (Gaulton)	Metabolic/Cardio/Anthropometric	ODC	1.62E-01	1.00E+00	2.76E-01	1.00E+00	NONE	Substantia nigra
Type 2 diabetes (Gaulton)	Metabolic/Cardio/Anthropometric	OPC	9.05E-01	1.00E+00	7.04E-01	1.00E+00	NONE	Substantia nigra
Ulcerative colitis	Immune	Astrocyte	7.25E-01	1.00E+00	3.01E-02	2.11E-01	NONE	Substantia nigra
Ulcerative colitis	Immune	DaN	2.05E-01	1.00E+00	6.56E-01	1.00E+00	NONE	Substantia nigra
Ulcerative colitis	Immune	Endothelial	5.08E-01	1.00E+00	5.31E-02	3.72E-01	NONE	Substantia nigra
Ulcerative colitis	Immune	GABA	3.06E-01	1.00E+00	9.34E-01	1.00E+00	NONE	Substantia nigra
Ulcerative colitis	Immune	Microglia	3.64E-01	1.00E+00	1.19E-05	8.30E-05	MAGMA**	Substantia nigra
Ulcerative colitis	Immune	ODC	9.94E-01	1.00E+00	4.03E-01	1.00E+00	NONE	Substantia nigra
Ulcerative colitis	Immune	OPC	4.29E-01	1.00E+00	7.57E-01	1.00E+00	NONE	Substantia nigra

Supplementary Table 6: Conditional cell-type analyses to evaluate whether multiple significant Substantia nigra cell-type signatures associated with the same trait coincide or not with the same genetic variants.

Trait	Cell1	Cell2	Coefficient_P_value
Bipolar	DaN	GABA	0.114578609
Bipolar	DaN	OPC	0.005807543
Bipolar	GABA	DaN	0.007432271
Bipolar	GABA	OPC	0.001150634
Bipolar	OPC	DaN	0.002973909
Bipolar	OPC	GABA	0.009572031
Schizophrenia	DaN	GABA	0.079004758
Schizophrenia	DaN	ODC	3.74E-08
Schizophrenia	DaN	OPC	0.000957182
Schizophrenia	GABA	DaN	0.012404314
Schizophrenia	GABA	ODC	2.60E-08
Schizophrenia	GABA	OPC	0.000804478
Schizophrenia	ODC	DaN	8.44E-05
Schizophrenia	ODC	GABA	2.28E-05
Schizophrenia	ODC	OPC	6.87E-05
Schizophrenia	OPC	DaN	0.012346818
Schizophrenia	OPC	GABA	0.032063874
Schizophrenia	OPC	ODC	8.40E-06
Autism's disorder	DaN	OPC	0.171010952
Autism's disorder	OPC	DaN	0.057078669
Neuroticism	GABA	OPC	0.045695054
Neuroticism	OPC	GABA	0.127004013
Education Years	GABA	OPC	0.02869337
Education Years	OPC	GABA	0.090233222
Parkinson's disease	DaN	ODC	0.010621566
Parkinson's disease	DaN	OPC	0.026781408
Parkinson's disease	ODC	DaN	0.007357132
Parkinson's disease	ODC	OPC	0.044201249
Parkinson's disease	OPC	DaN	0.860303267
Parkinson's disease	OPC	ODC	0.365362925

Supplementary Table 7: Risk association p-values for 30 brain and non-brain-related traits with cell-types from the Cortex using LDSC & MAGMA

Trait	Category	Cell	Pvalue_LDSC	Qvalue_LDSC	Pvalue_MAGMA	Qvalue_MAGMA	Methods	Region
ADHD	Psychatric disorder	Astrocyte	5.61E-01	1.00E+00	3.67E-01	1.00E+00	NONE	Cortex
ADHD	Psychatric disorder	In neurons	7.18E-01	1.00E+00	5.68E-01	1.00E+00	NONE	Cortex
ADHD	Psychatric disorder	Microglia	5.17E-01	1.00E+00	3.67E-01	1.00E+00	NONE	Cortex
ADHD	Psychatric disorder	ODC	5.54E-01	1.00E+00	1.77E-01	1.00E+00	NONE	Cortex
ADHD	Psychatric disorder	OPC	7.20E-01	1.00E+00	2.01E-01	1.00E+00	NONE	Cortex
ADHD	Psychatric disorder	Ex neurons	9.55E-03	5.73E-02	7.76E-04	4.65E-03	MAGMA** & LDSC*	Cortex
Alzheimer's disease(Jansenetal)	Neurological disorder	Astrocyte	7.35E-01	1.00E+00	3.79E-01	1.00E+00	NONE	Cortex
Alzheimer's disease(Jansenetal)	Neurological disorder	In neurons	4.27E-01	1.00E+00	1.45E-01	8.69E-01	NONE	Cortex
Alzheimer's disease(Jansenetal)	Neurological disorder	Microglia	1.74E-03	1.04E-02	1.19E-04	7.17E-04	Both**	Cortex
Alzheimer's disease(Jansenetal)	Neurological disorder	ODC	8.83E-02	5.30E-01	2.97E-01	1.00E+00	NONE	Cortex
Alzheimer's disease(Jansenetal)	Neurological disorder	OPC	9.00E-01	1.00E+00	4.77E-01	1.00E+00	NONE	Cortex
Alzheimer's disease(Jansenetal)	Neurological disorder	Ex neurons	4.36E-01	1.00E+00	5.52E-01	1.00E+00	NONE	Cortex
Amyotrophic Lateral Sclerosis	Neurological disorder	Astrocyte	7.62E-01	1.00E+00	6.37E-01	1.00E+00	NONE	Cortex
Amyotrophic Lateral Sclerosis	Neurological disorder	In neurons	9.45E-01	1.00E+00	8.48E-01	1.00E+00	NONE	Cortex
Amyotrophic Lateral Sclerosis	Neurological disorder	Microglia	6.87E-01	1.00E+00	3.06E-02	1.84E-01	NONE	Cortex
Amyotrophic Lateral Sclerosis	Neurological disorder	ODC	3.64E-01	1.00E+00	3.98E-01	1.00E+00	NONE	Cortex
Amyotrophic Lateral Sclerosis	Neurological disorder	OPC	8.35E-01	1.00E+00	5.51E-01	1.00E+00	NONE	Cortex
Amyotrophic Lateral Sclerosis	Neurological disorder	Ex neurons	5.54E-02	3.32E-01	2.63E-01	1.00E+00	NONE	Cortex
Anorexia	Psychatric disorder	Astrocyte	8.47E-01	1.00E+00	9.49E-01	1.00E+00	NONE	Cortex
Anorexia	Psychatric disorder	In neurons	5.30E-01	1.00E+00	8.33E-01	1.00E+00	NONE	Cortex
Anorexia	Psychatric disorder	Microglia	5.33E-01	1.00E+00	9.76E-01	1.00E+00	NONE	Cortex
Anorexia	Psychatric disorder	ODC	9.32E-01	1.00E+00	9.00E-01	1.00E+00	NONE	Cortex
Anorexia	Psychatric disorder	OPC	7.27E-01	1.00E+00	2.17E-01	1.00E+00	NONE	Cortex
Anorexia	Psychatric disorder	Ex neurons	3.58E-01	1.00E+00	1.46E-01	8.77E-01	NONE	Cortex
Anxiety	Psychatric disorder	Astrocyte	7.11E-01	1.00E+00	3.69E-01	1.00E+00	NONE	Cortex
Anxiety	Psychatric disorder	In neurons	6.06E-01	1.00E+00	1.49E-02	8.93E-02	NONE	Cortex
Anxiety	Psychatric disorder	Microglia	2.26E-01	1.00E+00	5.09E-01	1.00E+00	NONE	Cortex
Anxiety	Psychatric disorder	ODC	5.79E-01	1.00E+00	5.33E-01	1.00E+00	NONE	Cortex
Anxiety	Psychatric disorder	OPC	5.61E-01	1.00E+00	6.39E-01	1.00E+00	NONE	Cortex
Anxiety	Psychatric disorder	Ex neurons	4.15E-02	2.49E-01	7.24E-03	4.34E-02	MAGMA** & LDSC*	Cortex
Autism disorder	Psychatric disorder	Astrocyte	1.61E-01	9.65E-01	3.49E-02	2.10E-01	NONE	Cortex
Autism disorder	Psychatric disorder	In neurons	7.61E-02	4.56E-01	1.08E-01	6.50E-01	NONE	Cortex
Autism disorder	Psychatric disorder	Microglia	7.61E-02	4.57E-01	1.54E-02	9.27E-02	NONE	Cortex
Autism disorder	Psychatric disorder	ODC	6.83E-02	4.10E-01	1.13E-01	6.77E-01	NONE	Cortex
Autism disorder	Psychatric disorder	OPC	2.82E-02	1.69E-01	1.32E-02	7.95E-02	NONE	Cortex
Autism disorder	Psychatric disorder	Ex neurons	3.00E-01	1.00E+00	3.39E-01	1.00E+00	NONE	Cortex
Bipolar	Psychatric disorder	Astrocyte	4.86E-01	1.00E+00	1.06E-01	6.34E-01	NONE	Cortex
Bipolar	Psychatric disorder	In neurons	4.57E-01	1.00E+00	3.94E-01	1.00E+00	NONE	Cortex
Bipolar	Psychatric disorder	Microglia	7.58E-01	1.00E+00	1.91E-01	1.00E+00	NONE	Cortex
Bipolar	Psychatric disorder	ODC	7.01E-01	1.00E+00	3.77E-02	2.26E-01	NONE	Cortex
Bipolar	Psychatric disorder	OPC	3.74E-01	1.00E+00	9.60E-02	5.76E-01	NONE	Cortex

Bipolar	Psychatric disorder	Ex neurons	6.80E-03	4.08E-02	2.89E-03	1.73E-02	Both**	Cortex
BMI	Metabolic/Cardio/Anthropometric	Astrocyte	4.52E-01	1.00E+00	3.23E-01	1.00E+00	NONE	Cortex
BMI	Metabolic/Cardio/Anthropometric	In neurons	8.97E-01	1.00E+00	3.91E-02	2.34E-01	NONE	Cortex
BMI	Metabolic/Cardio/Anthropometric	Microglia	8.50E-01	1.00E+00	5.11E-01	1.00E+00	NONE	Cortex
BMI	Metabolic/Cardio/Anthropometric	ODC	4.04E-01	1.00E+00	1.41E-01	8.49E-01	NONE	Cortex
BMI	Metabolic/Cardio/Anthropometric	OPC	5.25E-01	1.00E+00	5.87E-02	3.52E-01	NONE	Cortex
BMI	Metabolic/Cardio/Anthropometric	Ex neurons	2.27E-02	1.36E-01	1.03E-03	6.16E-03	MAGMA** & LDSC*	Cortex
Coronary Artery Disease	Metabolic/Cardio/Anthropometric	Astrocyte	4.96E-01	1.00E+00	9.32E-05	5.59E-04	MAGMA**	Cortex
Coronary Artery Disease	Metabolic/Cardio/Anthropometric	In neurons	7.84E-01	1.00E+00	9.80E-02	5.88E-01	NONE	Cortex
Coronary Artery Disease	Metabolic/Cardio/Anthropometric	Microglia	3.00E-01	1.00E+00	4.93E-03	2.96E-02	MAGMA**	Cortex
Coronary Artery Disease	Metabolic/Cardio/Anthropometric	ODC	5.97E-01	1.00E+00	4.26E-02	2.55E-01	NONE	Cortex
Coronary Artery Disease	Metabolic/Cardio/Anthropometric	OPC	4.35E-01	1.00E+00	3.08E-02	1.85E-01	NONE	Cortex
Coronary Artery Disease	Metabolic/Cardio/Anthropometric	Ex neurons	2.11E-01	1.00E+00	4.27E-01	1.00E+00	NONE	Cortex
Crohn Disease	Immune	Astrocyte	2.79E-01	1.00E+00	5.55E-02	3.33E-01	NONE	Cortex
Crohn Disease	Immune	In neurons	3.04E-01	1.00E+00	2.43E-01	1.00E+00	NONE	Cortex
Crohn Disease	Immune	Microglia	3.26E-01	1.00E+00	1.79E-03	1.07E-02	MAGMA**	Cortex
Crohn Disease	Immune	ODC	7.41E-01	1.00E+00	3.47E-01	1.00E+00	NONE	Cortex
Crohn Disease	Immune	OPC	1.18E-01	7.11E-01	1.18E-01	7.10E-01	NONE	Cortex
Crohn Disease	Immune	Ex neurons	2.83E-01	1.00E+00	3.99E-01	1.00E+00	NONE	Cortex
Depressive Symptoms	Psychatric disorder	Astrocyte	6.04E-01	1.00E+00	2.32E-01	1.00E+00	NONE	Cortex
Depressive Symptoms	Psychatric disorder	In neurons	1.20E-01	7.17E-01	3.16E-02	1.90E-01	NONE	Cortex
Depressive Symptoms	Psychatric disorder	Microglia	7.85E-01	1.00E+00	2.58E-01	1.00E+00	NONE	Cortex
Depressive Symptoms	Psychatric disorder	ODC	8.92E-01	1.00E+00	6.85E-01	1.00E+00	NONE	Cortex
Depressive Symptoms	Psychatric disorder	OPC	4.28E-02	2.57E-01	2.75E-03	1.65E-02	MAGMA** & LDSC*	Cortex
Depressive Symptoms	Psychatric disorder	Ex neurons	2.75E-01	1.00E+00	4.86E-01	1.00E+00	NONE	Cortex
Educations Years	Cog.	Astrocyte	7.41E-01	1.00E+00	1.23E-01	7.40E-01	NONE	Cortex
Educations Years	Cog.	In neurons	2.41E-01	1.00E+00	1.39E-01	8.37E-01	NONE	Cortex
Educations Years	Cog.	Microglia	8.06E-02	4.83E-01	1.93E-01	1.00E+00	NONE	Cortex
Educations Years	Cog.	ODC	8.80E-02	5.28E-01	7.02E-03	4.21E-02	MAGMA**	Cortex
Educations Years	Cog.	OPC	3.88E-01	1.00E+00	7.74E-02	4.64E-01	NONE	Cortex
Educations Years	Cog.	Ex neurons	1.87E-03	1.12E-02	4.77E-04	2.86E-03	Both**	Cortex
Epilepsy (All forms)	Neurological disorder	Astrocyte	6.76E-01	1.00E+00	1.78E-01	1.00E+00	NONE	Cortex
Epilepsy (All forms)	Neurological disorder	In neurons	1.06E-01	6.36E-01	5.21E-01	1.00E+00	NONE	Cortex
Epilepsy (All forms)	Neurological disorder	Microglia	2.14E-01	1.00E+00	5.64E-02	3.39E-01	NONE	Cortex
Epilepsy (All forms)	Neurological disorder	ODC	3.71E-01	1.00E+00	1.89E-01	1.00E+00	NONE	Cortex
Epilepsy (All forms)	Neurological disorder	OPC	2.09E-02	1.26E-01	1.54E-01	9.26E-01	NONE	Cortex
Epilepsy (All forms)	Neurological disorder	Ex neurons	5.15E-02	3.09E-01	1.41E-01	8.46E-01	NONE	Cortex
Epilepsy (Focal)	Neurological disorder	Astrocyte	7.00E-01	1.00E+00	1.01E-01	6.05E-01	NONE	Cortex
Epilepsy (Focal)	Neurological disorder	In neurons	2.90E-02	1.74E-01	3.23E-01	1.00E+00	NONE	Cortex
Epilepsy (Focal)	Neurological disorder	Microglia	2.09E-01	1.00E+00	3.29E-01	1.00E+00	NONE	Cortex
Epilepsy (Focal)	Neurological disorder	ODC	4.00E-01	1.00E+00	3.34E-01	1.00E+00	NONE	Cortex
Epilepsy (Focal)	Neurological disorder	OPC	8.01E-02	4.81E-01	1.60E-01	9.61E-01	NONE	Cortex
Epilepsy (Focal)	Neurological disorder	Ex neurons	2.28E-01	1.00E+00	7.69E-02	4.61E-01	NONE	Cortex
Epilepsy (General)	Neurological disorder	Astrocyte	8.23E-01	1.00E+00	9.31E-01	1.00E+00	NONE	Cortex
Epilepsy (General)	Neurological disorder	In neurons	6.60E-01	1.00E+00	1.19E-01	7.14E-01	NONE	Cortex
Epilepsy (General)	Neurological disorder	Microglia	5.21E-01	1.00E+00	8.70E-02	5.22E-01	NONE	Cortex

Epilepsy (General)	Neurological disorder	ODC	2.78E-01	1.00E+00	3.74E-01	1.00E+00	NONE	Cortex
Epilepsy (General)	Neurological disorder	OPC	2.91E-01	1.00E+00	4.93E-01	1.00E+00	NONE	Cortex
Epilepsy (General)	Neurological disorder	Ex neurons	1.41E-02	8.43E-02	4.70E-01	1.00E+00	NONE	Cortex
Fasting Glucose	Metabolic/Cardio/Anthropometric	Astrocyte	6.95E-01	1.00E+00	7.59E-02	4.56E-01	NONE	Cortex
Fasting Glucose	Metabolic/Cardio/Anthropometric	In neurons	3.54E-01	1.00E+00	7.97E-01	1.00E+00	NONE	Cortex
Fasting Glucose	Metabolic/Cardio/Anthropometric	Microglia	8.12E-01	1.00E+00	8.11E-02	4.86E-01	NONE	Cortex
Fasting Glucose	Metabolic/Cardio/Anthropometric	ODC	4.10E-01	1.00E+00	4.92E-02	2.95E-01	NONE	Cortex
Fasting Glucose	Metabolic/Cardio/Anthropometric	OPC	4.94E-01	1.00E+00	2.00E-01	1.00E+00	NONE	Cortex
Fasting Glucose	Metabolic/Cardio/Anthropometric	Ex neurons	5.13E-01	1.00E+00	8.01E-01	1.00E+00	NONE	Cortex
Height	Metabolic/Cardio/Anthropometric	Astrocyte	1.05E-01	6.32E-01	3.36E-05	2.02E-04	MAGMA**	Cortex
Height	Metabolic/Cardio/Anthropometric	In neurons	9.39E-02	5.64E-01	5.32E-02	3.19E-01	NONE	Cortex
Height	Metabolic/Cardio/Anthropometric	Microglia	5.38E-01	1.00E+00	1.22E-05	7.33E-05	MAGMA**	Cortex
Height	Metabolic/Cardio/Anthropometric	ODC	1.21E-01	7.26E-01	1.65E-05	9.89E-05	MAGMA**	Cortex
Height	Metabolic/Cardio/Anthropometric	OPC	6.81E-02	4.08E-01	1.88E-04	1.13E-03	MAGMA**	Cortex
Height	Metabolic/Cardio/Anthropometric	Ex neurons	9.99E-01	1.00E+00	9.59E-01	1.00E+00	NONE	Cortex
Insomnia	Psychatric disorder	Astrocyte	8.38E-01	1.00E+00	1.15E-01	6.88E-01	NONE	Cortex
Insomnia	Psychatric disorder	In neurons	5.01E-02	3.00E-01	7.59E-02	4.55E-01	NONE	Cortex
Insomnia	Psychatric disorder	Microglia	5.87E-01	1.00E+00	7.30E-01	1.00E+00	NONE	Cortex
Insomnia	Psychatric disorder	ODC	9.24E-01	1.00E+00	2.24E-01	1.00E+00	NONE	Cortex
Insomnia	Psychatric disorder	OPC	5.35E-01	1.00E+00	2.89E-01	1.00E+00	NONE	Cortex
Insomnia	Psychatric disorder	Ex neurons	8.64E-01	1.00E+00	4.61E-01	1.00E+00	NONE	Cortex
Intelligence	Cog.	Astrocyte	2.70E-01	1.00E+00	3.42E-01	1.00E+00	NONE	Cortex
Intelligence	Cog.	In neurons	8.11E-01	1.00E+00	3.73E-01	1.00E+00	NONE	Cortex
Intelligence	Cog.	Microglia	6.31E-02	3.78E-01	8.08E-02	4.85E-01	NONE	Cortex
Intelligence	Cog.	ODC	5.63E-02	3.38E-01	2.71E-01	1.00E+00	NONE	Cortex
Intelligence	Cog.	OPC	1.33E-01	7.98E-01	1.20E-02	7.21E-02	NONE	Cortex
Intelligence	Cog.	Ex neurons	8.84E-02	5.30E-01	9.92E-02	5.95E-01	NONE	Cortex
Lipid HDL	Metabolic/Cardio/Anthropometric	Astrocyte	9.64E-02	5.79E-01	6.04E-03	3.62E-02	MAGMA**	Cortex
Lipid HDL	Metabolic/Cardio/Anthropometric	In neurons	1.29E-01	7.77E-01	1.22E-02	7.30E-02	NONE	Cortex
Lipid HDL	Metabolic/Cardio/Anthropometric	Microglia	2.20E-01	1.00E+00	6.08E-04	3.65E-03	MAGMA**	Cortex
Lipid HDL	Metabolic/Cardio/Anthropometric	ODC	3.45E-01	1.00E+00	1.53E-03	9.16E-03	MAGMA**	Cortex
Lipid HDL	Metabolic/Cardio/Anthropometric	OPC	1.17E-01	6.99E-01	6.73E-04	4.04E-03	MAGMA**	Cortex
Lipid HDL	Metabolic/Cardio/Anthropometric	Ex neurons	9.82E-01	1.00E+00	9.25E-01	1.00E+00	NONE	Cortex
Lipid LDL	Metabolic/Cardio/Anthropometric	Astrocyte	2.70E-02	1.62E-01	1.06E-01	6.33E-01	NONE	Cortex
Lipid LDL	Metabolic/Cardio/Anthropometric	In neurons	8.61E-01	1.00E+00	3.78E-01	1.00E+00	NONE	Cortex
Lipid LDL	Metabolic/Cardio/Anthropometric	Microglia	8.45E-02	5.07E-01	6.29E-02	3.77E-01	NONE	Cortex
Lipid LDL	Metabolic/Cardio/Anthropometric	ODC	4.94E-02	2.96E-01	7.26E-02	4.35E-01	NONE	Cortex
Lipid LDL	Metabolic/Cardio/Anthropometric	OPC	6.35E-01	1.00E+00	7.82E-01	1.00E+00	NONE	Cortex
Lipid LDL	Metabolic/Cardio/Anthropometric	Ex neurons	5.34E-01	1.00E+00	3.21E-01	1.00E+00	NONE	Cortex
Lipid TC	Metabolic/Cardio/Anthropometric	Astrocyte	2.73E-02	1.64E-01	3.09E-01	1.00E+00	NONE	Cortex
Lipid TC	Metabolic/Cardio/Anthropometric	In neurons	5.80E-01	1.00E+00	6.98E-01	1.00E+00	NONE	Cortex
Lipid TC	Metabolic/Cardio/Anthropometric	Microglia	5.71E-02	3.43E-01	4.80E-03	2.88E-02	MAGMA**	Cortex
Lipid TC	Metabolic/Cardio/Anthropometric	ODC	1.23E-01	7.35E-01	3.14E-01	1.00E+00	NONE	Cortex
Lipid TC	Metabolic/Cardio/Anthropometric	OPC	5.86E-01	1.00E+00	5.38E-01	1.00E+00	NONE	Cortex
Lipid TC	Metabolic/Cardio/Anthropometric	Ex neurons	8.64E-01	1.00E+00	2.85E-01	1.00E+00	NONE	Cortex
Lipid TG	Metabolic/Cardio/Anthropometric	Astrocyte	1.08E-01	6.46E-01	1.65E-01	9.92E-01	NONE	Cortex

Lipid TG	Metabolic/Cardio/Anthropometric	In neurons	7.67E-01	1.00E+00	2.61E-01	1.00E+00	NONE	Cortex
Lipid TG	Metabolic/Cardio/Anthropometric	Microglia	4.66E-02	2.80E-01	2.03E-02	1.22E-01	NONE	Cortex
Lipid TG	Metabolic/Cardio/Anthropometric	ODC	1.55E-01	9.30E-01	2.36E-02	1.42E-01	NONE	Cortex
Lipid TG	Metabolic/Cardio/Anthropometric	OPC	1.22E-01	7.32E-01	1.20E-01	7.19E-01	NONE	Cortex
Lipid TG	Metabolic/Cardio/Anthropometric	Ex neurons	8.52E-01	1.00E+00	2.20E-01	1.00E+00	NONE	Cortex
Major Depression	Psychatric disorder	Astrocyte	8.90E-01	1.00E+00	3.28E-01	1.00E+00	NONE	Cortex
Major Depression	Psychatric disorder	In neurons	1.96E-05	1.18E-04	1.91E-02	1.14E-01	LDSC** & MAGMA*	Cortex
Major Depression	Psychatric disorder	Microglia	1.00E+00	1.00E+00	8.89E-01	1.00E+00	NONE	Cortex
Major Depression	Psychatric disorder	ODC	9.17E-01	1.00E+00	9.46E-01	1.00E+00	NONE	Cortex
Major Depression	Psychatric disorder	OPC	2.82E-01	1.00E+00	4.20E-01	1.00E+00	NONE	Cortex
Major Depression	Psychatric disorder	Ex neurons	3.28E-01	1.00E+00	5.82E-02	3.49E-01	NONE	Cortex
Neuroticism	Psychatric disorder	Astrocyte	8.31E-01	1.00E+00	4.32E-02	2.59E-01	NONE	Cortex
Neuroticism	Psychatric disorder	In neurons	5.62E-03	3.37E-02	9.50E-04	5.70E-03	Both**	Cortex
Neuroticism	Psychatric disorder	Microglia	9.79E-01	1.00E+00	4.60E-01	1.00E+00	NONE	Cortex
Neuroticism	Psychatric disorder	ODC	8.84E-01	1.00E+00	2.89E-01	1.00E+00	NONE	Cortex
Neuroticism	Psychatric disorder	OPC	8.41E-02	5.05E-01	2.08E-05	1.25E-04	MAGMA**	Cortex
Neuroticism	Psychatric disorder	Ex neurons	4.85E-03	2.91E-02	1.23E-02	7.38E-02	LDSC** & MAGMA*	Cortex
Parkinson disease	Neurological disorder	Astrocyte	8.92E-01	1.00E+00	8.87E-03	5.32E-02	NONE	Cortex
Parkinson disease	Neurological disorder	In neurons	9.71E-01	1.00E+00	8.10E-01	1.00E+00	NONE	Cortex
Parkinson disease	Neurological disorder	Microglia	7.50E-01	1.00E+00	9.11E-02	5.46E-01	NONE	Cortex
Parkinson disease	Neurological disorder	ODC	4.90E-02	2.94E-01	2.33E-02	1.40E-01	NONE	Cortex
Parkinson disease	Neurological disorder	OPC	5.73E-01	1.00E+00	4.21E-02	2.53E-01	NONE	Cortex
Parkinson disease	Neurological disorder	Ex neurons	4.31E-02	2.58E-01	1.03E-03	6.16E-03	MAGMA** & LDSC*	Cortex
Rheumatoid Arthritis	Immune	Astrocyte	9.89E-01	1.00E+00	5.88E-02	3.53E-01	NONE	Cortex
Rheumatoid Arthritis	Immune	In neurons	1.75E-01	1.00E+00	1.90E-01	1.00E+00	NONE	Cortex
Rheumatoid Arthritis	Immune	Microglia	4.17E-01	1.00E+00	9.68E-02	5.81E-01	NONE	Cortex
Rheumatoid Arthritis	Immune	ODC	8.62E-01	1.00E+00	2.13E-02	1.28E-01	NONE	Cortex
Rheumatoid Arthritis	Immune	OPC	9.60E-01	1.00E+00	4.27E-01	1.00E+00	NONE	Cortex
Rheumatoid Arthritis	Immune	Ex neurons	8.98E-01	1.00E+00	3.82E-01	1.00E+00	NONE	Cortex
Schizophrenia (2018)	Psychatric disorder	Astrocyte	4.81E-01	1.00E+00	2.90E-02	1.74E-01	NONE	Cortex
Schizophrenia (2018)	Psychatric disorder	In neurons	3.15E-02	1.89E-01	2.19E-05	1.31E-04	MAGMA** & LDSC*	Cortex
Schizophrenia (2018)	Psychatric disorder	Microglia	2.04E-01	1.00E+00	1.39E-02	8.34E-02	NONE	Cortex
Schizophrenia (2018)	Psychatric disorder	ODC	5.31E-02	3.18E-01	1.64E-06	9.86E-06	MAGMA**	Cortex
Schizophrenia (2018)	Psychatric disorder	OPC	3.84E-02	2.30E-01	2.36E-04	1.42E-03	MAGMA** & LDSC*	Cortex
Schizophrenia (2018)	Psychatric disorder	Ex neurons	2.32E-04	1.39E-03	1.29E-07	7.75E-07	Both**	Cortex
Tourette Syndrome	Psychatric disorder	Astrocyte	2.63E-01	1.00E+00	7.42E-02	4.45E-01	NONE	Cortex
Tourette Syndrome	Psychatric disorder	In neurons	5.76E-01	1.00E+00	1.63E-01	9.80E-01	NONE	Cortex
Tourette Syndrome	Psychatric disorder	Microglia	7.85E-02	4.71E-01	1.20E-02	7.22E-02	NONE	Cortex
Tourette Syndrome	Psychatric disorder	ODC	2.30E-03	1.38E-02	1.95E-02	1.17E-01	LDSC** & MAGMA*	Cortex
Tourette Syndrome	Psychatric disorder	OPC	6.93E-02	4.16E-01	7.98E-03	4.79E-02	MAGMA**	Cortex
Tourette Syndrome	Psychatric disorder	Ex neurons	3.53E-03	2.12E-02	1.01E-03	6.09E-03	Both**	Cortex
Type 2 diabetes (Gaulton)	Metabolic/Cardio/Anthropometric	Astrocyte	3.23E-01	1.00E+00	2.37E-01	1.00E+00	NONE	Cortex
Type 2 diabetes (Gaulton)	Metabolic/Cardio/Anthropometric	In neurons	9.18E-01	1.00E+00	9.82E-01	1.00E+00	NONE	Cortex
Type 2 diabetes (Gaulton)	Metabolic/Cardio/Anthropometric	Microglia	2.60E-01	1.00E+00	1.04E-01	6.24E-01	NONE	Cortex
Type 2 diabetes (Gaulton)	Metabolic/Cardio/Anthropometric	ODC	6.53E-01	1.00E+00	2.16E-02	1.29E-01	NONE	Cortex
Type 2 diabetes (Gaulton)	Metabolic/Cardio/Anthropometric	OPC	4.52E-01	1.00E+00	2.86E-02	1.71E-01	NONE	Cortex

Type 2 diabetes (Gaulton)	Metabolic/Cardio/Anthropometric	Ex neurons	1.22E-01	7.31E-01	9.04E-02	5.43E-01	NONE	Cortex
Ulcerative colitis	Immune	Astrocyte	9.37E-01	1.00E+00	5.55E-02	3.33E-01	NONE	Cortex
Ulcerative colitis	Immune	In neurons	5.39E-01	1.00E+00	2.43E-01	1.00E+00	NONE	Cortex
Ulcerative colitis	Immune	Microglia	7.05E-01	1.00E+00	1.79E-03	1.07E-02	MAGMA**	Cortex
Ulcerative colitis	Immune	ODC	7.04E-01	1.00E+00	3.47E-01	1.00E+00	NONE	Cortex
Ulcerative colitis	Immune	OPC	2.16E-01	1.00E+00	1.18E-01	7.10E-01	NONE	Cortex
Ulcerative colitis	Immune	Ex neurons	9.77E-02	5.86E-01	3.99E-01	1.00E+00	NONE	Cortex

Supplementary Table 8: Conditional cell-type analysis to evaluate whether a significant Microglia/OPC/ODC/Neurons detected simultaneously in the SN & cortex with the same trait coincide or not with the same genetic variants.

Atlas1	Cell1	Atlas2	Cell2	GWA	P-value (LDSC) (Original Cellular Atlas))	P-value (LDSC) (Matched Cellular Atlas)
SN	Microglia	Cortex	Microglia	Alzheimer's disease	0.014224028	0.847836066
SN	DaN	Cortex	In neurons	Schizophrenia	6.42E-05	0.000967932
SN	DaN	Cortex	Ex neurons	Schizophrenia	0.000259705	0.002295987
SN	GABA	Cortex	In neurons	Schizophrenia	5.93E-05	0.003811003
SN	GABA	Cortex	Ex neurons	Schizophrenia	0.000291526	0.008352035
SN	ODC	Cortex	ODC	Schizophrenia	0.014427592	0.216332078
SN	ODC	Cortex	OPC	Schizophrenia	0.056113268	0.005955183
SN	OPC	Cortex	ODC	Schizophrenia	0.000370757	0.000210072
SN	OPC	Cortex	OPC	Schizophrenia	0.004954273	0.001314577
SN	OPC	Cortex	OPC	Neuroticism	0.001314577	0.330261148
SN	GABA	Cortex	In neurons	Neuroticism	5.93E-05	0.174078781
SN	GABA	Cortex	Ex neurons	Neuroticism	0.000291526	0.066208922
SN	GABA	Cortex	In neurons	Major Depression	5.93E-05	0.05399817
SN	GABA	Cortex	Ex neurons	Education Years	0.000291526	0.02839125
SN	OPC	Cortex	ODC	Education Years	0.000210072	0.352050827
SN	GABA	Cortex	In neurons	Bipolar	5.93E-05	0.004425257
SN	GABA	Cortex	Ex neurons	Bipolar	0.000291526	0.010707199
SN	GABA	Cortex	Ex neurons	ADHD	0.000291526	0.783605292
SN	DaN	Cortex	Ex neurons	Parkinson's disease	0.042929872	0.152615645

Supplementary Table 9: Risk association p-value for 30 brain and non-brain-related traits with GTEx brain region tissue using LDSC & MAGMA.

Trait	Category	Tissue	Pvalue_LDSC	Qvalue_LDSC	Pvalue_MAGMA	Qvalue_MAGMA	Methods
ADHD	Psychatric disorder	Amygdala	0.843786097	1	0.66183	1	NONE
ADHD	Psychatric disorder	Anterior_cingulate_cortex	0.416480367	1	0.12278	1	NONE
ADHD	Psychatric disorder	Caudate	0.702401287	1	0.025126	0.326638	NONE
ADHD	Psychatric disorder	Cerebellar_Hemisphere	0.253728495	1	0.23061	1	NONE
ADHD	Psychatric disorder	Cerebellum	0.235157503	1	0.2207	1	NONE
ADHD	Psychatric disorder	Cortex	0.212142499	1	0.0015922	0.0206986	MAGMA**
ADHD	Psychatric disorder	Frontal_Cortex	0.128531007	1	0.003211	0.041743	MAGMA**
ADHD	Psychatric disorder	Hippocampus	0.727709971	1	0.87439	1	NONE
ADHD	Psychatric disorder	Hypothalamus	0.745228312	1	0.93139	1	NONE
ADHD	Psychatric disorder	Nucleus_accumbens	0.615538563	1	0.031785	0.413205	NONE
ADHD	Psychatric disorder	Putamen	0.68713332	1	0.2756	1	NONE
ADHD	Psychatric disorder	Spinal_cord	0.966858502	1	0.99822	1	NONE
ADHD	Psychatric disorder	Substantia_nigra	0.899672085	1	0.92551	1	NONE
Alzheimer's disease (Jansenetal)	Neurological disorder	Amygdala	0.121654528	1	0.48206	1	NONE
Alzheimer's disease (Jansenetal)	Neurological disorder	Anterior_cingulate_cortex	0.19865084	1	0.44979	1	NONE
Alzheimer's disease (Jansenetal)	Neurological disorder	Caudate	0.167267913	1	0.081579	1	NONE
Alzheimer's disease (Jansenetal)	Neurological disorder	Cerebellar_Hemisphere	0.877608952	1	0.72311	1	NONE
Alzheimer's disease (Jansenetal)	Neurological disorder	Cerebellum	0.871312959	1	0.47568	1	NONE
Alzheimer's disease (Jansenetal)	Neurological disorder	Cortex	0.13046283	1	0.21933	1	NONE
Alzheimer's disease (Jansenetal)	Neurological disorder	Frontal_Cortex	0.484615561	1	0.3901	1	NONE
Alzheimer's disease (Jansenetal)	Neurological disorder	Hippocampus	0.06508845	0.846149844	0.35757	1	NONE
Alzheimer's disease (Jansenetal)	Neurological disorder	Hypothalamus	0.186727465	1	0.97949	1	NONE
Alzheimer's disease (Jansenetal)	Neurological disorder	Nucleus_accumbens	0.197825446	1	0.51384	1	NONE
Alzheimer's disease (Jansenetal)	Neurological disorder	Putamen	0.220845919	1	0.35405	1	NONE
Alzheimer's disease (Jansenetal)	Neurological disorder	Spinal_cord	0.00706361	0.09182693	0.052219	0.678847	NONE
Alzheimer's disease (Jansenetal)	Neurological disorder	Substantia_nigra	0.00355496	0.046214482	0.030262	0.393406	LDSC** & MAGMA*
Amyotrophic Lateral Sclerosis	Neurological disorder	Amygdala	0.951999635	1	0.94028	1	NONE
Amyotrophic Lateral Sclerosis	Neurological disorder	Anterior_cingulate_cortex	0.37933667	1	0.54995	1	NONE
Amyotrophic Lateral Sclerosis	Neurological disorder	Caudate	0.852476504	1	0.47157	1	NONE
Amyotrophic Lateral Sclerosis	Neurological disorder	Cerebellar_Hemisphere	0.239601849	1	0.41188	1	NONE
Amyotrophic Lateral Sclerosis	Neurological disorder	Cerebellum	0.075955526	0.987421834	0.12364	1	NONE
Amyotrophic Lateral Sclerosis	Neurological disorder	Cortex	0.004223952	0.054911381	0.22856	1	NONE
Amyotrophic Lateral Sclerosis	Neurological disorder	Frontal_Cortex	0.001926769	0.025047996	0.43596	1	LDSC**
Amyotrophic Lateral Sclerosis	Neurological disorder	Hippocampus	0.952303021	1	0.93719	1	NONE
Amyotrophic Lateral Sclerosis	Neurological disorder	Hypothalamus	0.757607609	1	0.94035	1	NONE
Amyotrophic Lateral Sclerosis	Neurological disorder	Nucleus_accumbens	0.805473027	1	0.91542	1	NONE
Amyotrophic Lateral Sclerosis	Neurological disorder	Putamen	0.340778768	1	0.47391	1	NONE
Amyotrophic Lateral Sclerosis	Neurological disorder	Spinal_cord	0.451709653	1	0.92914	1	NONE
Amyotrophic Lateral Sclerosis	Neurological disorder	Substantia_nigra	0.671130797	1	0.73941	1	NONE
Anorexia	Psychatric disorder	Amygdala	0.023582887	0.306577528	0.23368	1	NONE
Anorexia	Psychatric disorder	Anterior_cingulate_cortex	0.163816145	1	0.11389	1	NONE
Anorexia	Psychatric disorder	Caudate	0.039790882	0.517281465	0.023458	0.304954	NONE
Anorexia	Psychatric disorder	Cerebellar_Hemisphere	0.320961201	1	0.46615	1	NONE
Anorexia	Psychatric disorder	Cerebellum	0.286707206	1	0.75539	1	NONE
Anorexia	Psychatric disorder	Cortex	0.207184576	1	0.67279	1	NONE

Anorexia	Psychatric disorder	Frontal_Cortex	0.147951838	1	0.42125	1	NONE
Anorexia	Psychatric disorder	Hippocampus	0.091480614	1	0.67736	1	NONE
Anorexia	Psychatric disorder	Hypothalamus	0.225682262	1	0.53188	1	NONE
Anorexia	Psychatric disorder	Nucleus_accumbens	0.092905671	1	0.065097	0.846261	NONE
Anorexia	Psychatric disorder	Putamen	0.244822544	1	0.68488	1	NONE
Anorexia	Psychatric disorder	Spinal_cord	0.547080155	1	0.76665	1	NONE
Anorexia	Psychatric disorder	Substantia_nigra	0.28538459	1	0.83971	1	NONE
Anxiety	Psychatric disorder	Amygdala	0.092221113	1	0.16473	1	NONE
Anxiety	Psychatric disorder	Anterior_cingulate_cortex	0.207852012	1	0.19264	1	NONE
Anxiety	Psychatric disorder	Caudate	0.74469644	1	0.47198	1	NONE
Anxiety	Psychatric disorder	Cerebellar_Hemisphere	0.203456844	1	0.01343	0.17459	NONE
Anxiety	Psychatric disorder	Cerebellum	0.275310825	1	0.61725	1	NONE
Anxiety	Psychatric disorder	Cortex	0.010962157	0.142508044	0.070309	0.914017	NONE
Anxiety	Psychatric disorder	Frontal_Cortex	0.019517647	0.253729416	0.01912	0.24856	NONE
Anxiety	Psychatric disorder	Hippocampus	0.042092241	0.547199134	0.53216	1	NONE
Anxiety	Psychatric disorder	Hypothalamus	0.734070259	1	0.92723	1	NONE
Anxiety	Psychatric disorder	Nucleus_accumbens	0.541469893	1	0.49997	1	NONE
Anxiety	Psychatric disorder	Putamen	0.590654046	1	0.7458	1	NONE
Anxiety	Psychatric disorder	Spinal_cord	0.813616373	1	0.7319	1	NONE
Anxiety	Psychatric disorder	Substantia_nigra	0.622042386	1	0.21544	1	NONE
Autism disorder	Psychatric disorder	Amygdala	0.61548395	1	0.57173	1	NONE
Autism disorder	Psychatric disorder	Anterior_cingulate_cortex	0.599073082	1	0.056714	0.737282	NONE
Autism disorder	Psychatric disorder	Caudate	0.63282825	1	0.5853	1	NONE
Autism disorder	Psychatric disorder	Cerebellar_Hemisphere	0.088889711	1	0.15087	1	NONE
Autism disorder	Psychatric disorder	Cerebellum	0.185163005	1	0.60595	1	NONE
Autism disorder	Psychatric disorder	Cortex	0.352148029	1	0.20875	1	NONE
Autism disorder	Psychatric disorder	Frontal_Cortex	0.187626314	1	0.38107	1	NONE
Autism disorder	Psychatric disorder	Hippocampus	0.543182711	1	0.64665	1	NONE
Autism disorder	Psychatric disorder	Hypothalamus	0.416999968	1	0.2991	1	NONE
Autism disorder	Psychatric disorder	Nucleus_accumbens	0.401109639	1	0.46722	1	NONE
Autism disorder	Psychatric disorder	Putamen	0.419711165	1	0.32737	1	NONE
Autism disorder	Psychatric disorder	Spinal_cord	0.181943623	1	0.52207	1	NONE
Autism disorder	Psychatric disorder	Substantia_nigra	0.225859978	1	0.83544	1	NONE
Bipolar	Psychatric disorder	Amygdala	0.532134827	1	0.8082	1	NONE
Bipolar	Psychatric disorder	Anterior_cingulate_cortex	0.006060908	0.078791807	0.11438	1	NONE
Bipolar	Psychatric disorder	Caudate	0.298553952	1	0.083027	1	NONE
Bipolar	Psychatric disorder	Cerebellar_Hemisphere	0.003064194	0.039834526	0.032531	0.422903	LDSC** & MAGMA*
Bipolar	Psychatric disorder	Cerebellum	0.024020788	0.312270248	0.0038259	0.0497367	MAGMA** & LDSC*
Bipolar	Psychatric disorder	Cortex	0.000321059	0.00417377	0.00053106	0.00690378	Both**
Bipolar	Psychatric disorder	Frontal_Cortex	0.000393272	0.00511253	0.0013307	0.0172991	Both**
Bipolar	Psychatric disorder	Hippocampus	0.541206559	1	0.20324	1	NONE
Bipolar	Psychatric disorder	Hypothalamus	0.914327845	1	0.99997	1	NONE
Bipolar	Psychatric disorder	Nucleus_accumbens	0.042144272	0.547875531	0.35089	1	NONE
Bipolar	Psychatric disorder	Putamen	0.247722024	1	0.029643	0.385359	NONE
Bipolar	Psychatric disorder	Spinal_cord	0.934283445	1	0.90401	1	NONE
Bipolar	Psychatric disorder	Substantia_nigra	0.802413503	1	0.93039	1	NONE
BMI	Metabolic/Cardio/Anthropometric	Amygdala	0.890635975	1	0.58552	1	NONE
BMI	Metabolic/Cardio/Anthropometric	Anterior_cingulate_cortex	0.023201698	0.301622068	0.0062159	0.0808067	NONE
BMI	Metabolic/Cardio/Anthropometric	Caudate	0.780922703	1	0.75794	1	NONE

BMI	Metabolic/Cardio/Anthropometric	Cerebellar_Hemisphere	0.019466683	0.253066873	0.0048652	0.0632476	NONE
BMI	Metabolic/Cardio/Anthropometric	Cerebellum	0.000104102	0.00135332	0.0036409	0.0473317	Both**
BMI	Metabolic/Cardio/Anthropometric	Cortex	0.001926005	0.02503806	0.00098734	0.01283542	Both**
BMI	Metabolic/Cardio/Anthropometric	Frontal_Cortex	0.013200714	0.171609284	0.0023202	0.0301626	MAGMA** & LDSC*
BMI	Metabolic/Cardio/Anthropometric	Hippocampus	0.860487224	1	0.98939	1	NONE
BMI	Metabolic/Cardio/Anthropometric	Hypothalamus	0.851994165	1	0.75614	1	NONE
BMI	Metabolic/Cardio/Anthropometric	Nucleus_accumbens	0.387212706	1	0.25133	1	NONE
BMI	Metabolic/Cardio/Anthropometric	Putamen	0.895572726	1	0.84178	1	NONE
BMI	Metabolic/Cardio/Anthropometric	Spinal_cord	0.908098177	1	0.98608	1	NONE
BMI	Metabolic/Cardio/Anthropometric	Substantia_nigra	0.981336436	1	0.91671	1	NONE
Coronary Artery Disease	Metabolic/Cardio/Anthropometric	Amygdala	0.039860459	0.518185972	0.15165	1	NONE
Coronary Artery Disease	Metabolic/Cardio/Anthropometric	Anterior_cingulate_cortex	0.157878697	1	0.085088	1	NONE
Coronary Artery Disease	Metabolic/Cardio/Anthropometric	Caudate	0.167303031	1	0.0021064	0.0273832	MAGMA**
Coronary Artery Disease	Metabolic/Cardio/Anthropometric	Cerebellar_Hemisphere	0.440684608	1	0.58281	1	NONE
Coronary Artery Disease	Metabolic/Cardio/Anthropometric	Cerebellum	0.03803327	0.49443251	0.16531	1	NONE
Coronary Artery Disease	Metabolic/Cardio/Anthropometric	Cortex	0.275492614	1	0.036676	0.476788	NONE
Coronary Artery Disease	Metabolic/Cardio/Anthropometric	Frontal_Cortex	0.104072063	1	0.25449	1	NONE
Coronary Artery Disease	Metabolic/Cardio/Anthropometric	Hippocampus	0.304696992	1	0.78089	1	NONE
Coronary Artery Disease	Metabolic/Cardio/Anthropometric	Hypothalamus	0.226745794	1	0.99383	1	NONE
Coronary Artery Disease	Metabolic/Cardio/Anthropometric	Nucleus_accumbens	0.32737581	1	0.73975	1	NONE
Coronary Artery Disease	Metabolic/Cardio/Anthropometric	Putamen	0.309876894	1	0.01104	0.14352	NONE
Coronary Artery Disease	Metabolic/Cardio/Anthropometric	Spinal_cord	0.08218428	1	0.74964	1	NONE
Coronary Artery Disease	Metabolic/Cardio/Anthropometric	Substantia_nigra	0.118499626	1	0.36787	1	NONE
Crohn Disease	Immune	Amygdala	0.654847748	1	0.60465	1	NONE
Crohn Disease	Immune	Anterior_cingulate_cortex	0.623621781	1	0.56889	1	NONE
Crohn Disease	Immune	Caudate	0.575407218	1	0.15893	1	NONE
Crohn Disease	Immune	Cerebellar_Hemisphere	0.245233058	1	0.21016	1	NONE
Crohn Disease	Immune	Cerebellum	0.121230204	1	0.45617	1	NONE
Crohn Disease	Immune	Cortex	0.727119656	1	0.76305	1	NONE
Crohn Disease	Immune	Frontal_Cortex	0.762653169	1	0.76131	1	NONE
Crohn Disease	Immune	Hippocampus	0.89423988	1	0.8649	1	NONE
Crohn Disease	Immune	Hypothalamus	0.899806632	1	0.97858	1	NONE
Crohn Disease	Immune	Nucleus_accumbens	0.812811782	1	0.83661	1	NONE
Crohn Disease	Immune	Putamen	0.508350851	1	0.12369	1	NONE
Crohn Disease	Immune	Spinal_cord	0.179687589	1	0.0096417	0.1253421	NONE
Crohn Disease	Immune	Substantia_nigra	0.86984102	1	0.0071574	0.0930462	NONE
Depressive Symptoms	Psychatric disorder	Amygdala	0.46622508	1	0.43332	1	NONE
Depressive Symptoms	Psychatric disorder	Anterior_cingulate_cortex	0.017943273	0.233262544	0.44631	1	NONE
Depressive Symptoms	Psychatric disorder	Caudate	0.849208241	1	0.889	1	NONE
Depressive Symptoms	Psychatric disorder	Cerebellar_Hemisphere	0.106903558	1	0.10406	1	NONE
Depressive Symptoms	Psychatric disorder	Cerebellum	0.056993323	0.740913197	0.17523	1	NONE
Depressive Symptoms	Psychatric disorder	Cortex	0.037352847	0.485587017	0.73205	1	NONE
Depressive Symptoms	Psychatric disorder	Frontal_Cortex	0.042903565	0.557746346	0.33644	1	NONE
Depressive Symptoms	Psychatric disorder	Hippocampus	0.995943269	1	0.97606	1	NONE
Depressive Symptoms	Psychatric disorder	Hypothalamus	0.622662725	1	0.43067	1	NONE
Depressive Symptoms	Psychatric disorder	Nucleus_accumbens	0.683524703	1	0.89961	1	NONE
Depressive Symptoms	Psychatric disorder	Putamen	0.834173267	1	0.86682	1	NONE
Depressive Symptoms	Psychatric disorder	Spinal_cord	0.998384224	1	0.74991	1	NONE
Depressive Symptoms	Psychatric disorder	Substantia_nigra	0.998500465	1	0.99068	1	NONE

Educations Years	Cog.	Amygdala	0.887348514	1	0.87532	1	NONE
Educations Years	Cog.	Anterior_cingulate_cortex	0.21441416	1	0.052323	0.680199	NONE
Educations Years	Cog.	Caudate	0.522692959	1	0.40875	1	NONE
Educations Years	Cog.	Cerebellar_Hemisphere	0.000203012	0.002639154	0.00058789	0.00764257	Both**
Educations Years	Cog.	Cerebellum	0.003999112	0.051988459	0.030728	0.399464	NONE
Educations Years	Cog.	Cortex	0.066522845	0.864796981	0.0098088	0.1275144	NONE
Educations Years	Cog.	Frontal_Cortex	0.251891013	1	0.042809	0.556517	NONE
Educations Years	Cog.	Hippocampus	0.647237321	1	0.3366	1	NONE
Educations Years	Cog.	Hypothalamus	0.848313075	1	0.95002	1	NONE
Educations Years	Cog.	Nucleus_accumbens	0.366072895	1	0.16087	1	NONE
Educations Years	Cog.	Putamen	0.3282622	1	0.32173	1	NONE
Educations Years	Cog.	Spinal_cord	0.821660454	1	0.99145	1	NONE
Educations Years	Cog.	Substantia_nigra	0.948266927	1	0.99972	1	NONE
Epilepsy (All forms)	Neurological disorder	Amygdala	0.790374152	1	0.38292	1	NONE
Epilepsy (All forms)	Neurological disorder	Anterior_cingulate_cortex	0.087734605	1	0.1373	1	NONE
Epilepsy (All forms)	Neurological disorder	Caudate	0.66979826	1	0.81011	1	NONE
Epilepsy (All forms)	Neurological disorder	Cerebellar_Hemisphere	0.002976109	0.038689419	0.065762	0.854906	LDSC**
Epilepsy (All forms)	Neurological disorder	Cerebellum	0.024322259	0.316189371	0.071993	0.935909	NONE
Epilepsy (All forms)	Neurological disorder	Cortex	0.183907458	1	0.20659	1	NONE
Epilepsy (All forms)	Neurological disorder	Frontal_Cortex	0.042680985	0.554852802	0.12754	1	NONE
Epilepsy (All forms)	Neurological disorder	Hippocampus	0.65798373	1	0.41679	1	NONE
Epilepsy (All forms)	Neurological disorder	Hypothalamus	0.842342884	1	0.96281	1	NONE
Epilepsy (All forms)	Neurological disorder	Nucleus_accumbens	0.991356409	1	0.97146	1	NONE
Epilepsy (All forms)	Neurological disorder	Putamen	0.433255347	1	0.3708	1	NONE
Epilepsy (All forms)	Neurological disorder	Spinal_cord	0.153137645	1	0.29926	1	NONE
Epilepsy (All forms)	Neurological disorder	Substantia_nigra	0.969652489	1	0.6563	1	NONE
Epilepsy (Focal)	Neurological disorder	Amygdala	0.734969528	1	0.36487	1	NONE
Epilepsy (Focal)	Neurological disorder	Anterior_cingulate_cortex	0.357420644	1	0.16499	1	NONE
Epilepsy (Focal)	Neurological disorder	Caudate	0.940972134	1	0.95974	1	NONE
Epilepsy (Focal)	Neurological disorder	Cerebellar_Hemisphere	0.025669622	0.333705089	0.44735	1	NONE
Epilepsy (Focal)	Neurological disorder	Cerebellum	0.079265311	1	0.42781	1	NONE
Epilepsy (Focal)	Neurological disorder	Cortex	0.315854339	1	0.04915	0.63895	NONE
Epilepsy (Focal)	Neurological disorder	Frontal_Cortex	0.219762537	1	0.37636	1	NONE
Epilepsy (Focal)	Neurological disorder	Hippocampus	0.340954386	1	0.41205	1	NONE
Epilepsy (Focal)	Neurological disorder	Hypothalamus	0.858489939	1	0.99039	1	NONE
Epilepsy (Focal)	Neurological disorder	Nucleus_accumbens	0.988902533	1	0.99435	1	NONE
Epilepsy (Focal)	Neurological disorder	Putamen	0.534170629	1	0.41034	1	NONE
Epilepsy (Focal)	Neurological disorder	Spinal_cord	0.042888728	0.557553461	0.77929	1	NONE
Epilepsy (Focal)	Neurological disorder	Substantia_nigra	0.911250835	1	0.94743	1	NONE
Epilepsy (General)	Neurological disorder	Amygdala	0.29544378	1	0.76186	1	NONE
Epilepsy (General)	Neurological disorder	Anterior_cingulate_cortex	0.092551545	1	0.11396	1	NONE
Epilepsy (General)	Neurological disorder	Caudate	0.903818745	1	0.89489	1	NONE
Epilepsy (General)	Neurological disorder	Cerebellar_Hemisphere	0.183213796	1	0.19909	1	NONE
Epilepsy (General)	Neurological disorder	Cerebellum	0.183326827	1	0.24861	1	NONE
Epilepsy (General)	Neurological disorder	Cortex	0.10374305	1	0.029441	0.382733	NONE
Epilepsy (General)	Neurological disorder	Frontal_Cortex	0.065650348	0.853454528	0.0013592	0.0176696	MAGMA**
Epilepsy (General)	Neurological disorder	Hippocampus	0.089552724	1	0.52327	1	NONE
Epilepsy (General)	Neurological disorder	Hypothalamus	0.013039116	0.169508513	0.76752	1	NONE
Epilepsy (General)	Neurological disorder	Nucleus_accumbens	0.743963258	1	0.69199	1	NONE

Epilepsy (General)	Neurological disorder	Putamen	0.771553484	1	0.94266	1	NONE
Epilepsy (General)	Neurological disorder	Spinal_cord	0.032679516	0.42483371	0.39266	1	NONE
Epilepsy (General)	Neurological disorder	Substantia_nigra	0.176528403	1	0.46946	1	NONE
Fasting Glucose	Metabolic/Cardio/Anthropometric	Amygdala	0.415317535	1	0.84118	1	NONE
Fasting Glucose	Metabolic/Cardio/Anthropometric	Anterior_cingulate_cortex	0.246960375	1	0.7978	1	NONE
Fasting Glucose	Metabolic/Cardio/Anthropometric	Caudate	0.219613299	1	0.062897	0.817661	NONE
Fasting Glucose	Metabolic/Cardio/Anthropometric	Cerebellar_Hemisphere	0.269508451	1	0.41411	1	NONE
Fasting Glucose	Metabolic/Cardio/Anthropometric	Cerebellum	0.333597285	1	0.68311	1	NONE
Fasting Glucose	Metabolic/Cardio/Anthropometric	Cortex	0.244441387	1	0.8769	1	NONE
Fasting Glucose	Metabolic/Cardio/Anthropometric	Frontal_Cortex	0.292306252	1	0.81292	1	NONE
Fasting Glucose	Metabolic/Cardio/Anthropometric	Hippocampus	0.672590829	1	0.22603	1	NONE
Fasting Glucose	Metabolic/Cardio/Anthropometric	Hypothalamus	0.481782276	1	0.9316	1	NONE
Fasting Glucose	Metabolic/Cardio/Anthropometric	Nucleus_accumbens	0.040821542	0.530680048	0.54909	1	NONE
Fasting Glucose	Metabolic/Cardio/Anthropometric	Putamen	0.435658618	1	0.12761	1	NONE
Fasting Glucose	Metabolic/Cardio/Anthropometric	Spinal_cord	0.842592201	1	0.33759	1	NONE
Fasting Glucose	Metabolic/Cardio/Anthropometric	Substantia_nigra	0.722361125	1	0.59667	1	NONE
Height (2014)	Metabolic/Cardio/Anthropometric	Amygdala	0.219309732	1	0.0096019	0.1248247	NONE
Height (2014)	Metabolic/Cardio/Anthropometric	Anterior_cingulate_cortex	0.994249284	1	0.91124	1	NONE
Height (2014)	Metabolic/Cardio/Anthropometric	Caudate	0.360504136	1	0.026786	0.348218	NONE
Height (2014)	Metabolic/Cardio/Anthropometric	Cerebellar_Hemisphere	0.647046999	1	0.041126	0.534638	NONE
Height (2014)	Metabolic/Cardio/Anthropometric	Cerebellum	0.48771979	1	0.019282	0.250666	NONE
Height (2014)	Metabolic/Cardio/Anthropometric	Cortex	0.970865696	1	0.84963	1	NONE
Height (2014)	Metabolic/Cardio/Anthropometric	Frontal_Cortex	0.999617388	1	0.99721	1	NONE
Height (2014)	Metabolic/Cardio/Anthropometric	Hippocampus	0.594620056	1	0.12437	1	NONE
Height (2014)	Metabolic/Cardio/Anthropometric	Hypothalamus	0.000100456	0.001305928	0.24005	1	LDSC**
Height (2014)	Metabolic/Cardio/Anthropometric	Nucleus_accumbens	0.845602121	1	0.63463	1	NONE
Height (2014)	Metabolic/Cardio/Anthropometric	Putamen	0.115139254	1	0.01385	0.18005	NONE
Height (2014)	Metabolic/Cardio/Anthropometric	Spinal_cord	0.995164173	1	0.98289	1	NONE
Height (2014)	Metabolic/Cardio/Anthropometric	Substantia_nigra	0.885326721	1	0.95666	1	NONE
Insomnia	Psychatric disorder	Amygdala	0.832372788	1	0.62458	1	NONE
Insomnia	Psychatric disorder	Anterior_cingulate_cortex	0.932176643	1	0.37435	1	NONE
Insomnia	Psychatric disorder	Caudate	0.923134429	1	0.61185	1	NONE
Insomnia	Psychatric disorder	Cerebellar_Hemisphere	0.863784416	1	0.52579	1	NONE
Insomnia	Psychatric disorder	Cerebellum	0.75493558	1	0.48552	1	NONE
Insomnia	Psychatric disorder	Cortex	0.637868447	1	0.61451	1	NONE
Insomnia	Psychatric disorder	Frontal_Cortex	0.68090397	1	0.13615	1	NONE
Insomnia	Psychatric disorder	Hippocampus	0.916255442	1	0.53475	1	NONE
Insomnia	Psychatric disorder	Hypothalamus	0.534115644	1	0.34057	1	NONE
Insomnia	Psychatric disorder	Nucleus_accumbens	0.550963148	1	0.65438	1	NONE
Insomnia	Psychatric disorder	Putamen	0.949157696	1	0.96535	1	NONE
Insomnia	Psychatric disorder	Spinal_cord	0.99910725	1	0.72419	1	NONE
Insomnia	Psychatric disorder	Substantia_nigra	0.851886623	1	0.73173	1	NONE
Intelligence	Cog.	Amygdala	0.295079222	1	0.41859	1	NONE
Intelligence	Cog.	Anterior_cingulate_cortex	0.035930416	0.467095414	0.019685	0.255905	NONE
Intelligence	Cog.	Caudate	0.543181617	1	0.56651	1	NONE
Intelligence	Cog.	Cerebellar_Hemisphere	0.007540348	0.098024529	0.040208	0.522704	NONE
Intelligence	Cog.	Cerebellum	0.297475841	1	0.086485	1	NONE
Intelligence	Cog.	Cortex	0.005945848	0.077296027	0.019141	0.248833	NONE
Intelligence	Cog.	Frontal_Cortex	0.002364413	0.030737365	0.033909	0.440817	LDSC** & MAGMA*

Intelligence	Cog.	Hippocampus	0.511091383	1	0.75865	1	NONE
Intelligence	Cog.	Hypothalamus	0.884552965	1	0.89904	1	NONE
Intelligence	Cog.	Nucleus_accumbens	0.209490087	1	0.09973	1	NONE
Intelligence	Cog.	Putamen	0.053307797	0.693001364	0.6328	1	NONE
Intelligence	Cog.	Spinal_cord	0.418123992	1	0.81807	1	NONE
Intelligence	Cog.	Substantia_nigra	0.594444108	1	0.69785	1	NONE
Lipid HDL	Metabolic/Cardio/Anthropometric	Amygdala	0.474638073	1	0.77391	1	NONE
Lipid HDL	Metabolic/Cardio/Anthropometric	Anterior_cingulate_cortex	0.279963428	1	0.59905	1	NONE
Lipid HDL	Metabolic/Cardio/Anthropometric	Caudate	0.005510749	0.071639737	0.056258	0.731354	NONE
Lipid HDL	Metabolic/Cardio/Anthropometric	Cerebellar_Hemisphere	0.313788622	1	0.052622	0.684086	NONE
Lipid HDL	Metabolic/Cardio/Anthropometric	Cerebellum	0.786216935	1	0.0015153	0.0196989	MAGMA**
Lipid HDL	Metabolic/Cardio/Anthropometric	Cortex	0.510234641	1	0.70414	1	NONE
Lipid HDL	Metabolic/Cardio/Anthropometric	Frontal_Cortex	0.41389275	1	0.62697	1	NONE
Lipid HDL	Metabolic/Cardio/Anthropometric	Hippocampus	0.278957759	1	0.77557	1	NONE
Lipid HDL	Metabolic/Cardio/Anthropometric	Hypothalamus	0.158373797	1	0.9463	1	NONE
Lipid HDL	Metabolic/Cardio/Anthropometric	Nucleus_accumbens	0.16693909	1	0.094754	1	NONE
Lipid HDL	Metabolic/Cardio/Anthropometric	Putamen	0.005824675	0.075720773	0.045069	0.585897	NONE
Lipid HDL	Metabolic/Cardio/Anthropometric	Spinal_cord	0.229658232	1	0.23475	1	NONE
Lipid HDL	Metabolic/Cardio/Anthropometric	Substantia_nigra	0.096495994	1	0.073137	0.950781	NONE
Lipid LDL	Metabolic/Cardio/Anthropometric	Amygdala	0.829956954	1	0.34717	1	NONE
Lipid LDL	Metabolic/Cardio/Anthropometric	Anterior_cingulate_cortex	0.732916869	1	0.89993	1	NONE
Lipid LDL	Metabolic/Cardio/Anthropometric	Caudate	0.034997927	0.454973045	0.11755	1	NONE
Lipid LDL	Metabolic/Cardio/Anthropometric	Cerebellar_Hemisphere	0.85244746	1	0.42873	1	NONE
Lipid LDL	Metabolic/Cardio/Anthropometric	Cerebellum	0.977833726	1	0.2328	1	NONE
Lipid LDL	Metabolic/Cardio/Anthropometric	Cortex	0.229307398	1	0.0945	1	NONE
Lipid LDL	Metabolic/Cardio/Anthropometric	Frontal_Cortex	0.661296985	1	0.6144	1	NONE
Lipid LDL	Metabolic/Cardio/Anthropometric	Hippocampus	0.809875034	1	0.15953	1	NONE
Lipid LDL	Metabolic/Cardio/Anthropometric	Hypothalamus	0.764739255	1	0.92885	1	NONE
Lipid LDL	Metabolic/Cardio/Anthropometric	Nucleus_accumbens	0.633377171	1	0.92814	1	NONE
Lipid LDL	Metabolic/Cardio/Anthropometric	Putamen	0.380484059	1	0.092791	1	NONE
Lipid LDL	Metabolic/Cardio/Anthropometric	Spinal_cord	0.689093892	1	0.35817	1	NONE
Lipid LDL	Metabolic/Cardio/Anthropometric	Substantia_nigra	0.348125988	1	0.0048197	0.0626561	NONE
Lipid TC	Metabolic/Cardio/Anthropometric	Amygdala	0.730071873	1	0.55461	1	NONE
Lipid TC	Metabolic/Cardio/Anthropometric	Anterior_cingulate_cortex	0.660091931	1	0.97588	1	NONE
Lipid TC	Metabolic/Cardio/Anthropometric	Caudate	0.009061769	0.117802996	0.20346	1	NONE
Lipid TC	Metabolic/Cardio/Anthropometric	Cerebellar_Hemisphere	0.776447029	1	0.091859	1	NONE
Lipid TC	Metabolic/Cardio/Anthropometric	Cerebellum	0.94838482	1	0.25891	1	NONE
Lipid TC	Metabolic/Cardio/Anthropometric	Cortex	0.185020331	1	0.14939	1	NONE
Lipid TC	Metabolic/Cardio/Anthropometric	Frontal_Cortex	0.499602222	1	0.8555	1	NONE
Lipid TC	Metabolic/Cardio/Anthropometric	Hippocampus	0.55958559	1	0.49888	1	NONE
Lipid TC	Metabolic/Cardio/Anthropometric	Hypothalamus	0.553684192	1	0.91675	1	NONE
Lipid TC	Metabolic/Cardio/Anthropometric	Nucleus_accumbens	0.413170916	1	0.83926	1	NONE
Lipid TC	Metabolic/Cardio/Anthropometric	Putamen	0.086515194	1	0.075902	0.986726	NONE
Lipid TC	Metabolic/Cardio/Anthropometric	Spinal_cord	0.475525631	1	0.63745	1	NONE
Lipid TC	Metabolic/Cardio/Anthropometric	Substantia_nigra	0.257509018	1	0.0093924	0.1221012	NONE
Lipid TG	Metabolic/Cardio/Anthropometric	Amygdala	0.251686789	1	0.84004	1	NONE
Lipid TG	Metabolic/Cardio/Anthropometric	Anterior_cingulate_cortex	0.294925465	1	0.89458	1	NONE
Lipid TG	Metabolic/Cardio/Anthropometric	Caudate	0.024047794	0.312621327	0.47282	1	NONE
Lipid TG	Metabolic/Cardio/Anthropometric	Cerebellar_Hemisphere	0.120009226	1	0.078872	1	NONE

Lipid TG	Metabolic/Cardio/Anthropometric	Cerebellum	0.292745682	1	0.03227	0.41951	NONE
Lipid TG	Metabolic/Cardio/Anthropometric	Cortex	0.084475995	1	0.382	1	NONE
Lipid TG	Metabolic/Cardio/Anthropometric	Frontal_Cortex	0.189191534	1	0.62022	1	NONE
Lipid TG	Metabolic/Cardio/Anthropometric	Hippocampus	0.552494059	1	0.64677	1	NONE
Lipid TG	Metabolic/Cardio/Anthropometric	Hypothalamus	0.20609831	1	0.96493	1	NONE
Lipid TG	Metabolic/Cardio/Anthropometric	Nucleus_accumbens	0.315182782	1	0.67866	1	NONE
Lipid TG	Metabolic/Cardio/Anthropometric	Putamen	0.128984153	1	0.20991	1	NONE
Lipid TG	Metabolic/Cardio/Anthropometric	Spinal_cord	0.590144523	1	0.24064	1	NONE
Lipid TG	Metabolic/Cardio/Anthropometric	Substantia_nigra	0.338804132	1	0.46509	1	NONE
Major Depression	Psychatric disorder	Amygdala	0.786680447	1	0.5096	1	NONE
Major Depression	Psychatric disorder	Anterior_cingulate_cortex	0.158483396	1	0.27421	1	NONE
Major Depression	Psychatric disorder	Caudate	0.71812775	1	0.51162	1	NONE
Major Depression	Psychatric disorder	Cerebellar_Hemisphere	0.06927901	0.900627124	0.018565	0.241345	NONE
Major Depression	Psychatric disorder	Cerebellum	0.022828082	0.29676507	0.0095736	0.1244568	NONE
Major Depression	Psychatric disorder	Cortex	0.025772951	0.335048365	0.077505	1	NONE
Major Depression	Psychatric disorder	Frontal_Cortex	0.051221217	0.665875817	0.091696	1	NONE
Major Depression	Psychatric disorder	Hippocampus	0.9393119	1	0.78885	1	NONE
Major Depression	Psychatric disorder	Hypothalamus	0.87678464	1	0.4899	1	NONE
Major Depression	Psychatric disorder	Nucleus_accumbens	0.222770506	1	0.75912	1	NONE
Major Depression	Psychatric disorder	Putamen	0.652138661	1	0.79165	1	NONE
Major Depression	Psychatric disorder	Spinal_cord	0.994356815	1	0.99117	1	NONE
Major Depression	Psychatric disorder	Substantia_nigra	0.987699795	1	0.99268	1	NONE
Neuroticism	Psychatric disorder	Amygdala	0.784452858	1	0.72413	1	NONE
Neuroticism	Psychatric disorder	Anterior_cingulate_cortex	0.447673216	1	0.38005	1	NONE
Neuroticism	Psychatric disorder	Caudate	0.462054207	1	0.31662	1	NONE
Neuroticism	Psychatric disorder	Cerebellar_Hemisphere	0.137099107	1	0.12861	1	NONE
Neuroticism	Psychatric disorder	Cerebellum	0.101146529	1	0.18837	1	NONE
Neuroticism	Psychatric disorder	Cortex	0.199615178	1	0.1309	1	NONE
Neuroticism	Psychatric disorder	Frontal_Cortex	0.188466443	1	0.078383	1	NONE
Neuroticism	Psychatric disorder	Hippocampus	0.947113402	1	0.6095	1	NONE
Neuroticism	Psychatric disorder	Hypothalamus	0.957471707	1	0.36282	1	NONE
Neuroticism	Psychatric disorder	Nucleus_accumbens	0.470765001	1	0.62321	1	NONE
Neuroticism	Psychatric disorder	Putamen	0.793091071	1	0.82845	1	NONE
Neuroticism	Psychatric disorder	Spinal_cord	0.979905199	1	0.92376	1	NONE
Neuroticism	Psychatric disorder	Substantia_nigra	0.944582563	1	0.98185	1	NONE
Parkinson disease	Neurological disorder	Amygdala	0.772135115	1	0.75569	1	NONE
Parkinson disease	Neurological disorder	Anterior_cingulate_cortex	0.162043158	1	0.084374	1	NONE
Parkinson disease	Neurological disorder	Caudate	0.932979485	1	0.74277	1	NONE
Parkinson disease	Neurological disorder	Cerebellar_Hemisphere	0.201321951	1	0.0061884	0.0804492	NONE
Parkinson disease	Neurological disorder	Cerebellum	0.314845748	1	0.0015232	0.0198016	MAGMA**
Parkinson disease	Neurological disorder	Cortex	0.092716619	1	0.023545	0.306085	NONE
Parkinson disease	Neurological disorder	Frontal_Cortex	0.03257016	0.42341208	0.0052058	0.0676754	NONE
Parkinson disease	Neurological disorder	Hippocampus	0.577054926	1	0.81949	1	NONE
Parkinson disease	Neurological disorder	Hypothalamus	0.666970551	1	0.90195	1	NONE
Parkinson disease	Neurological disorder	Nucleus_accumbens	0.417505565	1	0.6477	1	NONE
Parkinson disease	Neurological disorder	Putamen	0.719419174	1	0.67269	1	NONE
Parkinson disease	Neurological disorder	Spinal_cord	0.506109397	1	0.61531	1	NONE
Parkinson disease	Neurological disorder	Substantia_nigra	0.578777338	1	0.36872	1	NONE
Rheumatoid Arthritis	Immune	Amygdala	0.536201066	1	0.27059	1	NONE

Rheumatoid Arthritis	Immune	Anterior_cingulate_cortex	0.961230764	1	0.55971	1	NONE
Rheumatoid Arthritis	Immune	Caudate	0.310837962	1	0.11791	1	NONE
Rheumatoid Arthritis	Immune	Cerebellar_Hemisphere	0.11071239	1	0.66648	1	NONE
Rheumatoid Arthritis	Immune	Cerebellum	0.226549515	1	0.73595	1	NONE
Rheumatoid Arthritis	Immune	Cortex	0.915359987	1	0.92192	1	NONE
Rheumatoid Arthritis	Immune	Frontal_Cortex	0.877203957	1	0.37332	1	NONE
Rheumatoid Arthritis	Immune	Hippocampus	0.299512463	1	0.2797	1	NONE
Rheumatoid Arthritis	Immune	Hypothalamus	0.501611738	1	0.5359	1	NONE
Rheumatoid Arthritis	Immune	Nucleus_accumbens	0.396173885	1	0.22292	1	NONE
Rheumatoid Arthritis	Immune	Putamen	0.159958251	1	0.32183	1	NONE
Rheumatoid Arthritis	Immune	Spinal_cord	0.126456698	1	0.47572	1	NONE
Rheumatoid Arthritis	Immune	Substantia_nigra	0.359380416	1	0.44019	1	NONE
Schizophrenia (2018)	Psychatric disorder	Amygdala	0.163887171	1	0.63139	1	NONE
Schizophrenia (2018)	Psychatric disorder	Anterior_cingulate_cortex	3.15E-07	4.09E-06	6.68E-06	8.68E-05	Both**
Schizophrenia (2018)	Psychatric disorder	Caudate	0.113654645	1	0.46657	1	NONE
Schizophrenia (2018)	Psychatric disorder	Cerebellar_Hemisphere	0.000815551	0.010602169	0.0022721	0.0295373	Both**
Schizophrenia (2018)	Psychatric disorder	Cerebellum	0.000285479	0.003711221	0.0097462	0.1267006	LDSC** & MAGMA*
Schizophrenia (2018)	Psychatric disorder	Cortex	1.05E-09	1.37E-08	3.91E-05	0.000507845	Both**
Schizophrenia (2018)	Psychatric disorder	Frontal_Cortex	3.40E-08	4.42E-07	5.85E-05	0.000760825	Both**
Schizophrenia (2018)	Psychatric disorder	Hippocampus	0.200531779	1	0.54445	1	NONE
Schizophrenia (2018)	Psychatric disorder	Hypothalamus	0.803470613	1	0.989	1	NONE
Schizophrenia (2018)	Psychatric disorder	Nucleus_accumbens	0.040022524	0.520292808	0.60146	1	NONE
Schizophrenia (2018)	Psychatric disorder	Putamen	0.086317764	1	0.43856	1	NONE
Schizophrenia (2018)	Psychatric disorder	Spinal_cord	0.586431816	1	0.51348	1	NONE
Schizophrenia (2018)	Psychatric disorder	Substantia_nigra	0.831266492	1	0.94459	1	NONE
Tourette Syndrome	Psychatric disorder	Amygdala	0.527102843	1	0.63174	1	NONE
Tourette Syndrome	Psychatric disorder	Anterior_cingulate_cortex	0.104431659	1	0.0090007	0.1170091	NONE
Tourette Syndrome	Psychatric disorder	Caudate	0.431174014	1	0.13546	1	NONE
Tourette Syndrome	Psychatric disorder	Cerebellar_Hemisphere	0.04693715	0.610182953	0.35695	1	NONE
Tourette Syndrome	Psychatric disorder	Cerebellum	0.039883513	0.518485667	0.53492	1	NONE
Tourette Syndrome	Psychatric disorder	Cortex	0.074795913	0.972346866	0.015513	0.201669	NONE
Tourette Syndrome	Psychatric disorder	Frontal_Cortex	0.015487436	0.201336673	0.020186	0.262418	NONE
Tourette Syndrome	Psychatric disorder	Hippocampus	0.63760828	1	0.56816	1	NONE
Tourette Syndrome	Psychatric disorder	Hypothalamus	0.332804867	1	0.87759	1	NONE
Tourette Syndrome	Psychatric disorder	Nucleus_accumbens	0.160201186	1	0.10805	1	NONE
Tourette Syndrome	Psychatric disorder	Putamen	0.298500412	1	0.09133	1	NONE
Tourette Syndrome	Psychatric disorder	Spinal_cord	0.740175676	1	0.83835	1	NONE
Tourette Syndrome	Psychatric disorder	Substantia_nigra	0.98004308	1	0.96452	1	NONE
Type 2 diabetes (Gaulton)	Metabolic/Cardio/Anthropometric	Amygdala	0.998584164	1	0.14417	1	NONE
Type 2 diabetes (Gaulton)	Metabolic/Cardio/Anthropometric	Anterior_cingulate_cortex	0.283468617	1	0.43801	1	NONE
Type 2 diabetes (Gaulton)	Metabolic/Cardio/Anthropometric	Caudate	0.529931882	1	0.0067658	0.0879554	NONE
Type 2 diabetes (Gaulton)	Metabolic/Cardio/Anthropometric	Cerebellar_Hemisphere	0.942227621	1	0.9671	1	NONE
Type 2 diabetes (Gaulton)	Metabolic/Cardio/Anthropometric	Cerebellum	0.914366747	1	0.98782	1	NONE
Type 2 diabetes (Gaulton)	Metabolic/Cardio/Anthropometric	Cortex	0.027576824	0.358498707	0.065833	0.855829	NONE
Type 2 diabetes (Gaulton)	Metabolic/Cardio/Anthropometric	Frontal_Cortex	0.105853063	1	0.39234	1	NONE
Type 2 diabetes (Gaulton)	Metabolic/Cardio/Anthropometric	Hippocampus	0.914848789	1	0.96454	1	NONE
Type 2 diabetes (Gaulton)	Metabolic/Cardio/Anthropometric	Hypothalamus	0.9173301	1	0.73938	1	NONE
Type 2 diabetes (Gaulton)	Metabolic/Cardio/Anthropometric	Nucleus_accumbens	0.411554036	1	0.060463	0.786019	NONE
Type 2 diabetes (Gaulton)	Metabolic/Cardio/Anthropometric	Putamen	0.766141995	1	0.076576	0.995488	NONE

Type 2 diabetes (Gaulton)	Metabolic/Cardio/Anthropometric	Spinal_cord	0.608232653	1	0.45467	1	NONE
Type 2 diabetes (Gaulton)	Metabolic/Cardio/Anthropometric	Substantia_nigra	0.995583675	1	0.47687	1	NONE
Ulcerative colitis	Immune	Amygdala	0.340606034	1	0.60465	1	NONE
Ulcerative colitis	Immune	Anterior_cingulate_cortex	0.146844267	1	0.56889	1	NONE
Ulcerative colitis	Immune	Caudate	0.615659713	1	0.15893	1	NONE
Ulcerative colitis	Immune	Cerebellar_Hemisphere	0.792221737	1	0.21016	1	NONE
Ulcerative colitis	Immune	Cerebellum	0.62137179	1	0.45617	1	NONE
Ulcerative colitis	Immune	Cortex	0.275256672	1	0.76305	1	NONE
Ulcerative colitis	Immune	Frontal_Cortex	0.161964104	1	0.76131	1	NONE
Ulcerative colitis	Immune	Hippocampus	0.426447571	1	0.8649	1	NONE
Ulcerative colitis	Immune	Hypothalamus	0.13304136	1	0.97858	1	NONE
Ulcerative colitis	Immune	Nucleus_accumbens	0.356823239	1	0.83661	1	NONE
Ulcerative colitis	Immune	Putamen	0.608267821	1	0.12369	1	NONE
Ulcerative colitis	Immune	Spinal_cord	0.281078002	1	0.0096417	0.1253421	NONE
Ulcerative colitis	Immune	Substantia_nigra	0.514750416	1	0.0071574	0.0930462	NONE

Supplementary References

1. Krishnaswami SR*, et al.* Using single nuclei for RNA-seq to capture the transcriptome of postmortem neurons. *Nat Protoc* **11**, 499-524 (2016).

2. Butler A, Hoffman P, Smibert P, Papalexi E, Satija R. Integrating single-cell transcriptomic data across different conditions, technologies, and species. *Nat Biotechnol* **36**, 411-420 (2018).

3. Blondel VD, Guillaume J-L, Lambiotte R, Lefebvre E. Fast unfolding of communities in large networks %U http://stacks.iop.org/1742-5468/2008/i=10/a=P10008. *Journal of Statistical Mechanics: Theory and Experiment* **2008**, P10008 (2008).

4. Lake BB*, et al.* Neuronal subtypes and diversity revealed by single-nucleus RNA sequencing of the human brain. *Science* **352**, 1586-1590 (2016).

5. Lake BB*, et al.* Integrative single-cell analysis of transcriptional and epigenetic states in the human adult brain. *Nat Biotechnol* **36**, 70-80 (2018).

6. Zheng B*, et al.* PGC-1alpha, a potential therapeutic target for early intervention in Parkinson's disease. *Sci Transl Med* **2**, 52ra73 (2010).

7. Carvalho BS, Irizarry RA. A framework for oligonucleotide microarray preprocessing. *Bioinformatics* **26**, 2363-2367 (2010).

8. Mastroeni D*, et al.* Laser-captured microglia in the Alzheimer's and Parkinson's brain reveal unique regional expression profiles and suggest a potential role for hepatitis B in the Alzheimer's brain. *Neurobiol Aging* **63**, 12-21 (2018).

9. Patro R, Duggal G, Love MI, Irizarry RA, Kingsford C. Salmon provides fast and bias-aware quantification of transcript expression. *Nat Methods* **14**, 417-419 (2017).

10. Soneson C, Love MI, Robinson MD. Differential analyses for RNA-seq: transcript-level estimates improve gene-level inferences. *F1000Res* **4**, 1521 (2015).

11. Love MI, Huber W, Anders S. Moderated estimation of fold change and dispersion for RNA-seq data with DESeq2. *Genome Biol* **15**, 550 (2014).

12. Ritchie ME*, et al.* limma powers differential expression analyses for RNA-sequencing and microarray studies. *Nucleic Acids Res* **43**, e47 (2015).

13. Leek JT, Johnson WE, Parker HS, Jaffe AE, Storey JD. The sva package for removing batch effects and other unwanted variation in high-throughput experiments. *Bioinformatics* **28**, 882-883 (2012).

14. Darmanis S*, et al.* A survey of human brain transcriptome diversity at the single cell level. *Proc Natl Acad Sci U S A* **112**, 7285-7290 (2015).

15. Finucane HK*, et al.* Heritability enrichment of specifically expressed genes identifies disease-relevant tissues and cell types. *Nat Genet* **50**, 621-629 (2018).

16. de Leeuw CA, Mooij JM, Heskes T, Posthuma D. MAGMA: generalized gene-set analysis of GWAS data. *PLoS Comput Biol* **11**, e1004219 (2015).

17. Skene NG*, et al.* Genetic identification of brain cell types underlying schizophrenia. *Nat Genet* **50**, 825-833 (2018).

18. Pardinas AF*, et al.* Common schizophrenia alleles are enriched in mutation-intolerant genes and in regions under strong background selection. *Nat Genet* **50**, 381-389 (2018).

19. Chatr-Aryamontri A*, et al.* The BioGRID interaction database: 2017 update. *Nucleic Acids Res* **45**, D369-D379 (2017).

20. Lopez Y, Nakai K, Patil A. HitPredict version 4: comprehensive reliability scoring of physical protein-protein interactions from more than 100 species. *Database (Oxford)* **2015**, (2015).

21. Orchard S*, et al.* The MIntAct project--IntAct as a common curation platform for 11 molecular interaction databases. *Nucleic Acids Res* **42**, D358-363 (2014).

22. Szklarczyk D*, et al.* STRING v10: protein-protein interaction networks, integrated over the tree of life. *Nucleic Acids Res* **43**, D447-452 (2015).

23. Ruepp A*, et al.* CORUM: the comprehensive resource of mammalian protein complexes--2009. *Nucleic Acids Res* **38**, D497-501 (2010).

24. Fabregat A*, et al.* The Reactome pathway Knowledgebase. *Nucleic Acids Res* **44**, D481-487 (2016).
