# Supplementary material for: A single-cell atlas of the human substantia nigra reveals cell-specific pathways associated with neurological disorders
Source: Nat Commun. 2020 Aug 21;11:4183. doi: 10.1038/s41467-020-17876-0 (PMC7442652; doi:10.1038/s41467-020-17876-0)
Supplement: Supplementary file 3 — Description of Additional Supplementary Files [file 41467_2020_17876_MOESM3_ESM.pdf]

Description of Additional Supplementary Files

File Name: Supplementary Data 1
Description: 10x genomics single nuclei RNA sequencing quality control metrics for samples

File Name: Supplementary Data 2
Description: Cortex-substantia nigra cell-type annotations

File Name: Supplementary Data 3
Description: Differentially expressed genes for cell-types and cell-type subpopulation clusters in the substantia nigra (SN) and cortex single nuclei RNAseq

File Name: Supplementary Data 4
Description: Cell-type specific gene sets generated using t-statistics for cortex and SN cell atlas

File Name: Supplementary Data 5
Description: Cell-type specific gene sets generated using t-statistics for cortex and SN cell atlas without the amyloidosis sample

File Name: Supplementary Data 6
Description: Top 100 GO enriched pathways for cell-type specific modules in substantia nigra

File Name: Supplementary Data 7
Description: Top 100 GO enriched pathways for cell-type specific modules in cortex
