# Supplementary material for: A single-cell atlas of the human substantia nigra reveals cell-specific pathways associated with neurological disorders
Source: Nat Commun. 2020 Aug 21;11:4183. doi: 10.1038/s41467-020-17876-0 (PMC7442652; doi:10.1038/s41467-020-17876-0)
Supplement: Supplementary file 11 — Reporting Summary [file 41467_2020_17876_MOESM11_ESM.pdf]

nature research

Corresponding author(s): Caleb webber

Last updated by author(s): 30-06-2020

Reporting Summary

Nature Research wishes to improve the reproducibility of the work that we publish. This form provides structure for consistency and transparency in reporting. For further information on Nature Research policies, see Authors & Referees and the Editorial Policy Checklist .

Statistics

For all statistical analyses, confirm that the following items are present in the figure legend, table legend, main text, or Methods section.

n/a	Confirmed	
☐	☒	The exact sample size (*n*) for each experimental group/condition, given as a discrete number and unit of measurement
☐	☒	A statement on whether measurements were taken from distinct samples or whether the same sample was measured repeatedly
☐	☒	The statistical test(s) used AND whether they are one- or two-sided<br>*Only common tests should be described solely by name; describe more complex techniques in the Methods section.*
☐	☒	A description of all covariates tested
☐	☒	A description of any assumptions or corrections, such as tests of normality and adjustment for multiple comparisons
☐	☒	A full description of the statistical parameters including central tendency (e.g. means) or other basic estimates (e.g. regression coefficient) AND variation (e.g. standard deviation) or associated estimates of uncertainty (e.g. confidence intervals)
☐	☒	For null hypothesis testing, the test statistic (e.g. *F*, *t*, *r*) with confidence intervals, effect sizes, degrees of freedom and *P* value noted<br>*Give P values as exact values whenever suitable.*
☒	☐	For Bayesian analysis, information on the choice of priors and Markov chain Monte Carlo settings
☐	☒	For hierarchical and complex designs, identification of the appropriate level for tests and full reporting of outcomes
☐	☒	Estimates of effect sizes (e.g. Cohen's *d*, Pearson's *r*), indicating how they were calculated

Our web collection on statistics for biologists contains articles on many of the points above.

Software and code

Policy information about availability of computer code

Data collection	No Software was used for data collection
Data analysis	We used the following open source and commercial software to analyse the data (parameters described in Methods/Supplementary Material):<br><br>(1) Cell Ranger 2.1.1<br>(2) Seurat R package (v2.3.4)<br>(3) LD score regression (LDSC) (Version 1.0.0)<br>(4) Multi-marker Analysis of GenoMic Annotation (MAGMA) (v1.06b)<br>(5) igraph” R package (1.2.4.2)<br>(6) topGO R package (2.38.1)<br>(7) Revigo (http://revigo.irb.hr/)<br>(8) oligo R package(1.52.0)<br>(9) Salmon (0.11.0)<br>(10) DESeq2 R package (1.16.1)<br>(11) limma R package (3.26.9)<br>(12) sva R package (3.36.0)

For manuscripts utilizing custom algorithms or software that are central to the research but not yet described in published literature, software must be made available to editors/reviewers. We strongly encourage code deposition in a community repository (e.g. GitHub). See the Nature Research guidelines for submitting code & software for further information.

Data

Policy information about availability of data

All manuscripts must include a data availability statement. This statement should provide the following information, where applicable:

- Accession codes, unique identifiers, or web links for publicly available datasets
- A list of figures that have associated raw data
- A description of any restrictions on data availability

The processed 10x 3' Chromium single nuclei RNAseq UMI-barcode matrices for each sample are available from the Gene Expression Omnibus under the accession code GSE140231.

Field-specific reporting

Please select the one below that is the best fit for your research. If you are not sure, read the appropriate sections before making your selection.

☒ Life sciences ☐ Behavioural & social sciences ☐ Ecological, evolutionary & environmental sciences

For a reference copy of the document with all sections, see nature.com/documents/nr-reporting-summary-flat.pdf

Life sciences study design

All studies must disclose on these points even when the disclosure is negative.

Sample size	We used post-mortem brain tisssue from 5 individuals. Informed consent had been collected from all cases fulfilling the requirements of the Human Tissue Act 2004. The sample size met the criteria of a minimum of 3 replicates per group for the study
Data exclusions	No data was excluded from the analysis
Replication	The reproducibility of experimental findings was confirmed by analyzing multiple matched samples and biological replicates across the Cortex and Substantia nigra. We observed consistent clustering by cell type between replicates, across samples and regions .
Randomization	Data from five control post-mortem brain tissues from donors was allocated in to groups based on the brain region. All individuals across groups were of similar age. To control for covariates not affecting the results and conclusions of this study (e.g. Sample 3 exhibited cerebral amyloid angiopathy), we repeated all analyses with and without the samples from this individual and found a very high correlation between results.
Blinding	Blinding was not relevant to this study as only 5 controls were selected on the basis of the absence of neurological clinical disease and group allocation was based on known brain regions of interest (cortex and substantia nigra)

Reporting for specific materials, systems and methods

We require information from authors about some types of materials, experimental systems and methods used in many studies. Here, indicate whether each material, system or method listed is relevant to your study. If you are not sure if a list item applies to your research, read the appropriate section before selecting a response.

Materials & experimental systems

n/a	Involved in the study	
☒	☐	Antibodies
☒	☐	Eukaryotic cell lines
☒	☐	Palaeontology
☒	☐	Animals and other organisms
☐	☒	Human research participants
☒	☐	Clinical data

Methods

n/a	Involved in the study	
☒	☐	ChIP-seq
☐	☒	Flow cytometry
☒	☐	MRI-based neuroimaging

Human research participants

Policy information about studies involving human research participants

Population characteristics	We used post-mortem brain tisssue from 5 individuals, selected on the basis of the absence of neurological clinical disease and by midbrain RNA integrity number (RIN) yielding scores over eight from the Oxford Brain Bank. Informed consent had been collected from all cases fulfilling the requirements of the Human Tissue Act 2004.
Recruitment	Post-mortem tissue was provided from what was available to the Oxford Brain Bank.
Ethics oversight	The tissue request was made by a clinical pathologist (author J.A.A) to the Oxford Brain Bank.

Note that full information on the approval of the study protocol must also be provided in the manuscript.

Flow Cytometry

Plots

Confirm that:

- [ ] The axis labels state the marker and fluorochrome used (e.g. CD4-FITC).
- [ ] The axis scales are clearly visible. Include numbers along axes only for bottom left plot of group (a 'group' is an analysis of identical markers).
- [ ] All plots are contour plots with outliers or pseudocolor plots.
- [ ] A numerical value for number of cells or percentage (with statistics) is provided.

Methodology

Sample preparation	[NOTE: We have provided the information below as this was requested by the journal but the FACS sorting used was only to sort nuclei from debris for sequencing and thus we do not provide a figure exemplifying the gating strategy requested below. We weren't separating out different types of same thing and 10X sequencing would only work if a nuclei was captured. Nonetheless, we provide all the other details requested.]<br><br>3mm2 pieces of fresh-frozen brain tissue were placed in homogenization buffer (250 mM sucrose, 25 mM KCl, 5 mM MgCl2, 10 mM Tris Buffer pH 8.0, 1 mM DTT, 1X protease inhibitor, 0.4 U ul-1 RNaseIN, 0.2 U ul-1 Superasin, 0.1% v/v Triton X-100) for 10 minutes prior to mechanical disruption with a dounce homogenizer. Homogenate was filtered through a 35 mM cell strainer before concentrating nuclei by centrifugation 400 g 5 minutes. Nuclei were resuspended in FACS buffer (1 XPBS, 1 RNase-Free BSA, 0.2 U µl−1 of RNasin Plus RNase inhibitor, 10 ng ml-1 Hoechst 33342) prior to FACS to obtain a population of nuclei free cellular debris.
Instrument	Hardware: MoFlo XDP equipped with:<br>488nm laser (Coherent Sapphire, 200mW)<br>355nm (UV) laser (JDSU XCyte 200mW)<br>fluorescent channel optical filter: 505 nm dichroic short-pass filter and 450/65nm band-pass filter.
Software	Software: Summit V.5.5.0.16880 (Beckman Coulter)
Cell population abundance	Effective isolation of nuclei after FACS-sorting was assessed by microscopy to determine if suspension was sufficient clear of debris to use in 10X microfluidic platform.
Gating strategy	We isolated all nuclei from the post-mortem brain preparation using flourescent DNA stain Hoechst 33342.

- [ ] Tick this box to confirm that a figure exemplifying the gating strategy is provided in the Supplementary Information.
