# Supplementary material for: A single-cell atlas of the human substantia nigra reveals cell-specific pathways associated with neurological disorders
Source: Nat Commun. 2020 Aug 21;11:4183. doi: 10.1038/s41467-020-17876-0 (PMC7442652; doi:10.1038/s41467-020-17876-0)
Supplement: Supplementary file 2 — Peer Review File [file 41467_2020_17876_MOESM2_ESM.pdf]

REVIEWER COMMENTS

Reviewer #1 (Remarks to the Author):

This paper presents a single cell transcriptomic atlas for the human Substantia nigra. They sequenced thousands of cells in the cortex and nigra from 5 postmortem human brains and used LD score regression (LDSC) and Multi-marker Analysis of GenoMic Annotation (MAGMA) to analyze genetic risks for parkinson's disease associated with expression patterns in specific cell types. They found association with dopamine neuron and oligodendrocyte-specific gene expression but no association with microglia and astrocytes, different from Alzheimer's disease. In addition, they also report associations between nigra neuron expression pattern with several other psychiatric disorders.

Given the paucity of data on human substantia nigra, the atlas from this study is highly valuable. In particular, they found a large difference in the relative proportions of neurons and glial cells between the cortex and nigra, indicating the importance of examining gene expression at the level of specific cell types rather than bulk brain tissues. However, I am not qualified to evaluate the analyses on disease association of the gene expression patterns to know whether the main conclusions are sufficiently supported. I will leave that to the other reviewers.

Reviewer #3 (Remarks to the Author):

In this work the Webber group generate a single cell atlas for Parkinson disease, by examining SN and cortical regions. The work is performed in a relatively modest number of samples. The results are mainly consistent with previous analysis of mouse single cell sequencing - suggesting that the majority of genetic risk loci have a functional context within DA neurons, interestingly the authors identified a distinct set within the oligodendrocyte population. That these are apparently distinct is one of the most interesting parts of this work.

The manuscript is refreshingly simple, precise, and clear - and this leads me to have relatively little to suggest.

Data availability - it would be wonderful to make these data available in a more useful way - GEO is a great step, but creating or leveraging an existing interactive portal would be useful for the PD community. I recognize this is not a small ask.

Gene selection and models used. While MAGMA and LDSC are quite commonly used methods within this particular discipline, I believe that that work is of interest to the broader community - some simple discussion of how genes from risk loci are selected and what potential biases exist, would be useful.

The authors mention that "We observed for the first time in human a significant association between PD genetic risk and genes with DaN-specific
expression patterns" - this is true, but it may be worth contrasting this with the work previously done using available reference data (mouse) - the latest Nalls GWA and the work by Reynolds et al.

Reviewer #4 (Remarks to the Author):

In this work, Agarwal et. al present a single-cell transcriptomic atlas of the human substantia nigra (SN). The authors perform in-depth analyses of this atlas and identify cell type-specific

associations between SN cell populations and risk genes for Parkinson's disease (PD), as well as for a range of neuropsychiatric, neurologic, and other complex traits. The authors complement this atlas with a transcriptional characterization of matched cortical samples, similarly associating risk genes with specific cell types in this region. This work substantiates existing knowledge of PD etiology by demonstrating, for the first time in humans, an association between PD risk genes and transcriptomics of SN dopaminergic neurons. In addition, this work highlights novel associations between PD risk genes and oligodendrocyte-specific gene expression. These data allow for a critical evaluation of neuronal and glial mechanisms contributing to the progression of PD and other brain disorders.

Overall, the key conclusions are well-supported by the data presented, which provide a valuable resource for the study of the SN in both homeostasis and disease. That said, additional commentary or revisions regarding comments below would strengthen the paper prior to publication.

• On Line 84-86, it seems that the authors rely on GO terms to conclude that the astrocyte-1 sub-cluster in SN samples is representative of A1 astrocytes, while the astrocyte-2 aligns with A2 astrocytes (supplementary Table 2). However, A1 and A2 astrocytes are defined by very specific gene expression programs, and therefore overlapping genes should be shown or described. Alternatively, revising the text to omit this classification can be done.

• According to Figure S1c, astrocyte-2 cells almost entirely come from the N2B patient sample. Similarly, microglia bias towards N3, N4B samples, and ODC-3 bias towards the N2B sample. Such big variations within the same region (sometimes from the sample patient as shown in Figure S12d) make it difficult to interpret the differences in proportions of cells between regions (line 102). In addition to possible biological differences, technical variations or regional features reacting differently to experimental procedures may also contribute to the observed regional differences in neuronal and glial cell proportions. This caveat should be noted.

• It is striking that the data show over 50% of post-mitotic cells are dividing (in G2M or S phases, Figure S1d), particularly in aged patient samples. This requires some explanation or validation.

• Key example networks showing DaN (Figure S10) and ODC modules (e.g. M5 or M1) associated with PD should be displayed as a panel in Figure 4 rather than in a supplementary figure.

Minor Comments:

• Line 109-110: A more concise and clearer subheading is needed.

• Line 97: Should refer to Figure 1d & e instead of Supplemental Figures 1d & e.

• Figure S5 is missing an explanation of what the sizes of the circles mean in this graph.

• The authors provide a clear description of their conditional analyses in the methods section, but a brief clarification in the main text would help readers more easily follow the conclusions drawn from these analyses (for ex: what does $p < 0.05$ mean in this context?).

• The authors show that converging OPC/ODC genes are also associated with a range of psychiatric conditions. This should be mentioned in the abstract and/or introduction, as this is an important and novel result of this work.

<u>Response to Reviewers</u>
"A human single-cell atlas of the Substantia nigra reveals novel cell-specific pathways associated with the genetic risk of Parkinson's disease and neuropsychiatric disorders."
by Agarwal et al.

To all Reviewers: We are genuinely grateful for all the time and effort the Reviewers have put into reviewing our manuscript and believe that their input has significantly enhanced the work. Thank you.

Reviewer #1:

Comments to the Author

[Reviewer #1] This paper presents a single cell transcriptomic atlas for the human Substantia nigra. They sequenced thousands of cells in the cortex and nigra from 5 postmortem human brains and used LD score regression (LDSC) and Multi-marker Analysis of GenoMic Annotation (MAGMA) to analyze genetic risks for parkinson's disease associated with expression patterns in specific cell types. They found association with dopamine neuron and oligodendrocyte-specific gene expression but no association with microglia and astrocytes, different from Alzheimer's disease. In addition, they also report associations between nigra neuron expression pattern with several other psychiatric disorders. Given the paucity of data on human substantia nigra, the atlas from this study is highly valuable. In particular, they found a large difference in the relative proportions of neurons and glial cells between the cortex and nigra, indicating the importance of examining gene expression at the level of specific cell types rather than bulk brain tissues. However, I am not qualified to evaluate the analyses on disease association of the gene expression patterns to know whether the main conclusions are sufficiently supported. I will leave that to the other reviewers.

[Authors] We thank the Reviewer for their appreciation of the importance of this study and we are pleased that they note many of our significant findings. We do understand that not everyone is equally versed in all technical areas but nonetheless, we are pleased that the need for this study and its key messages were welcomed.

Reviewer #3:
Comments to the Author

[Reviewer #3] In this work the Webber group generate a single cell atlas for Parkinson disease, by examining SN and cortical regions. The work is performed in a relatively modest number of samples. The results are mainly consistent with previous analysis of mouse single cell sequencing - suggesting that the majority of genetic risk loci have a functional context within DA neurons, interestingly the authors identified a distinct set within the oligodendrocyte population. That these are apparently distinct is one of the most interesting parts of this work. The manuscript is refreshingly simple, precise, and clear - and this leads me to have relatively little to suggest.

[Authors] We thank the Reviewer for their appreciation of the work and its presentation. We note a very recent study using mouse (not human) data that confirms our unexpected association between PD genetic risk and oligodendrocytes and have added a reference in the text to this study [1].

[Discussion p6]: "*The association we discover between PD genetic risk variants and human oligodendrocyte-specific gene expression has very recently been proposed in an independent study* [23] *using mouse transcriptomic data.*".

[Reviewer #3] Data availability - it would be wonderful to make these data available in a more useful way - GEO is a great step, but creating or leveraging an existing interactive portal would be useful for the PD community. I recognize this is not a small ask.

[Authors] We agree with the Reviewer #3 that is not a small task (!) BUT we have provided this data to the IPDGC efforts to annotate Parkinson's risk variants. Thus, much of our nigral single cell atlas is accessible via an interactive portal through the IPDGC GWAS locus browser application developed by Grenn *et al.* (https://www.biorxiv.org/content/10.1101/2020.04.01.020404v1). The PD community can examine the expression of protein coding genes using our single cell atlas of their favourite GWA loci by using this web-interface developed by Green and *al*.. The Figure below show an example with *LRRK2* GWA loci, how a user can examine the expression of *LRRK2* by using this SN single cell atlas.

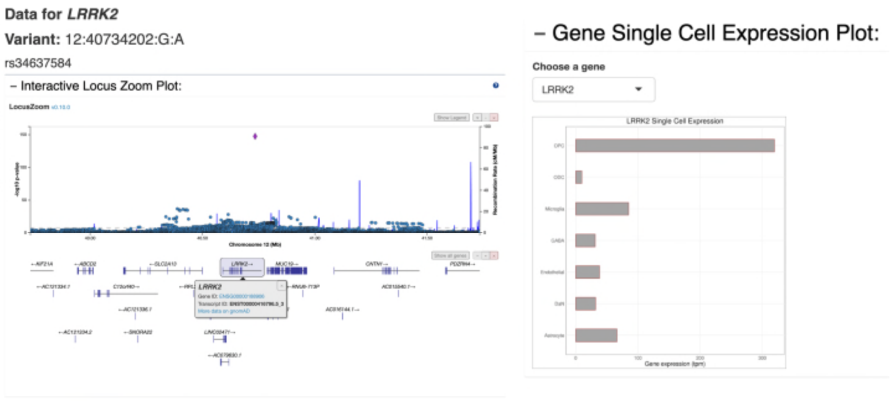

Figure: Example with LRRK2 Locus of the Parkinson's Disease GWAS Locus Browser.

[Reviewer #3] Gene selection and models used. While MAGMA and LDSC are quite commonly used methods within this particular discipline, I believe that that work is of interest to the broader community - some simple discussion of how genes from risk loci are selected and what potential biases exist, would be useful.

[Authors] We agree with the Reviewer #3, that the methods (MAGMA vs LDSC), gene cell-specificity expression measure and some parameters such locus definition, gene set definition used to perform cell association analysis with the risk loci for a specific trait is key and can affect the results. Unfortunately, there is currently no consensus in parameterising this kind of analysis. For example Skene et al. (2018) [2], Watanabe et al. (2019) [3], Nalls et al. [4] (2018) use distinct approaches and parameters.

We considered the results of both methods MAGMA and LDSC and due to the limited space, we justified in detail the selected parameters in the Supplemental material, section cell type association analysis (p 4-5). We used the same approach than Finucane et al. (2018) [5] which is to fit parameters maximising the well-known cell association between Excitatory Neurons and Schizophrenia. To help the reader, we also published in Github an RMarkdown document, detailing the different command steps to generate these gene sets and perform cell-type association We have referenced the availability of this code in the Supplementary materials p5 https://github.com/csandorfr/SN_Atlas.

[Reviewer #3] The authors mention that "We observed for the first time in human a significant association between PD genetic risk and genes with DaN-specific expression patterns" - this is true, but it may be worth contrasting this with the work previously done using available reference data (mouse) - the latest Nalls GWA and the work by Reynolds et al.

[Authors] As requested, we have edited the text to acknowledge recently published cell-association analyses including: Nalls *et al.* (2019) [4], Bryois *et al.* (2019) [1]:

[Results p3]: "*We observed for the first time in human a significant association between PD genetic risk and genes with DaN-specific expression patterns (q_{MAGMA}=4.6x10-3, Figure 2a), supported by recent observations made using mouse expression patterns of predicted PD GWA-risk genes* [12, 13] *and contrasting studies proposing that PD risk is not associated with neurons*[14]."

As mentioned above, we also cite that an oligodendrocyte association with PD risk has been recently reported with mouse transcriptomics (Bryois *et al.* (2020) [1]) (Discussion p6)

Reviewer #4:

Comments to the Author

[Reviewer #4] In this work, Agarwal et. al present a single-cell transcriptomic atlas of the human substantia nigra (SN). The authors perform in-depth analyses of this atlas and identify cell type-specific associations between SN cell populations and risk genes for Parkinson's disease (PD), as well as for a range of neuropsychiatric, neurologic, and other complex traits. The authors complement this atlas with a transcriptional characterization of matched cortical samples, similarly associating risk genes with specific cell types in this region. This work substantiates existing knowledge of PD etiology by demonstrating, for the first time in humans, an association between PD risk genes and transcriptomics of SN dopaminergic neurons. In addition, this work highlights novel associations between PD risk genes and oligodendrocyte-specific gene expression. These data allow for a critical evaluation of neuronal and glial mechanisms contributing to the progression of PD and other brain disorders.

Overall, the key conclusions are well-supported by the data presented, which provide a valuable resource for the study of the SN in both homeostasis and disease. That said, additional commentary or revisions regarding comments below would strengthen the paper prior to publication.

[Authors] We thank the Reviewer for acknowledging the utility of this data and we are delighted to release it (already released on GEO) and our analyses to the PD Community. We very much hope it will accelerate therapeutic research in PD and other disorders.

[Reviewer #4] •On Line 84-86, it seems that the authors rely on GO terms to conclude that the astrocyte-1 sub-cluster in SN samples is representative of A1 astrocytes, while the astrocyte-2 aligns with A2 astrocytes (supplementary Table 2). However, A1 and A2 astrocytes are defined by very specific gene expression programs, and therefore overlapping genes should be shown or described. Alternatively, revising the text to omit this classification can be done.

[Authors] Acknowledging the Reviewer's note regarding the specific marker gene-based classification of "A1" and "A2" astrocytes, we have modified the text to omit classifying these populations as those specific previously-defined astrocyte types and removed the reference:

[Results, line 84]: "*which included (i) astrocytes (GFAP) with two subtypes: astrocyte-1 population expressing neuro-inflammatory genes (OLR1) and an astrocyte-2 (GINS3) population expressing genes associated with growth and reparative functions (Supplementary Table 2 & Supplementary Figure 2)*[8]"

[Reviewer #4] •According to Figure S1c, astrocyte-2 cells almost entirely come from the N2B patient sample. Similarly, microglia bias towards N3, N4B samples, and ODC-3 bias towards the N2B sample. Such big variations within the same region (sometimes from the sample patient as shown in Figure S12d) make it difficult to interpret the differences in proportions of cells between regions (line 102). In addition to possible biological differences, technical variations or regional features reacting differently to experimental procedures may also contribute to the observed regional differences in neuronal and glial cell proportions. This caveat should be noted.

[Authors] Reviewer #4 is absolutely right that there are significant variation in the cell types obtained from each sample, even when sampled from the same individual. As all cell types from different

individuals cluster well together, we did not see confounding genotypic effects. Furthermore, recent bioinformatics predictions using mouse cell types to predict composition of GTeX tissues also predicts a striking difference in neuron/glia composition between the cortex and the nigra [6]. https://www.nature.com/articles/s41467-020-14561-0/figures/4

Nonetheless, the Reviewer's concern is well-taken and should be pointed out the Reader. Thus, we have both modified the text to highlight to readers that there is large variation between samples in cell type and now support our finding of this compositional difference with this recently published GTeX analyses.

[Results, Line 100]: "*The observed proportions of different cell populations in our SN atlas were consistent with histopathological studies, reporting oligodendrocytes as the most frequent glial cell population (45-75% across all brain regions, 62% for SN)* [9] *and recent bioinformatics predictions of tissue cell-type composition*[10]*. Despite significant variation in the numbers of cell types captured between inter- and intra-individual samples (Supplementary Table 3b), we observed consistent clustering by cell type between replicates, across samples and regions (Supplementary Figure 5), suggesting we have repeatedly captured the same resident cell populations.*"

[Reviewer #4] It is striking that the data show over 50% of post-mitotic cells are dividing (in G2M or S phases, Figure S1d), particularly in aged patient samples. This requires some explanation or validation.

[Authors] We are extremely grateful to the Reviewer #4 for pointing this out and are somewhat embarrassed. It unclear why this analysis was performed, let alone made its way into the manuscript. Nonetheless, it now falls to us to explain this odd result! We examined the Lake *et al.* 2018 [7] SC cortex data and found that was also called with a remarkable proportion of cells processing through cell cycle irrespective of their cell-type identify (**Figure below**). Thus, we believe this to be a methodological issue. As the identification the cell cycle was irrelevant here and this analysis do not affect the general conclusion of our study, we removed these panel of Figure S1 & S3. We apologise again for its initial inclusion.

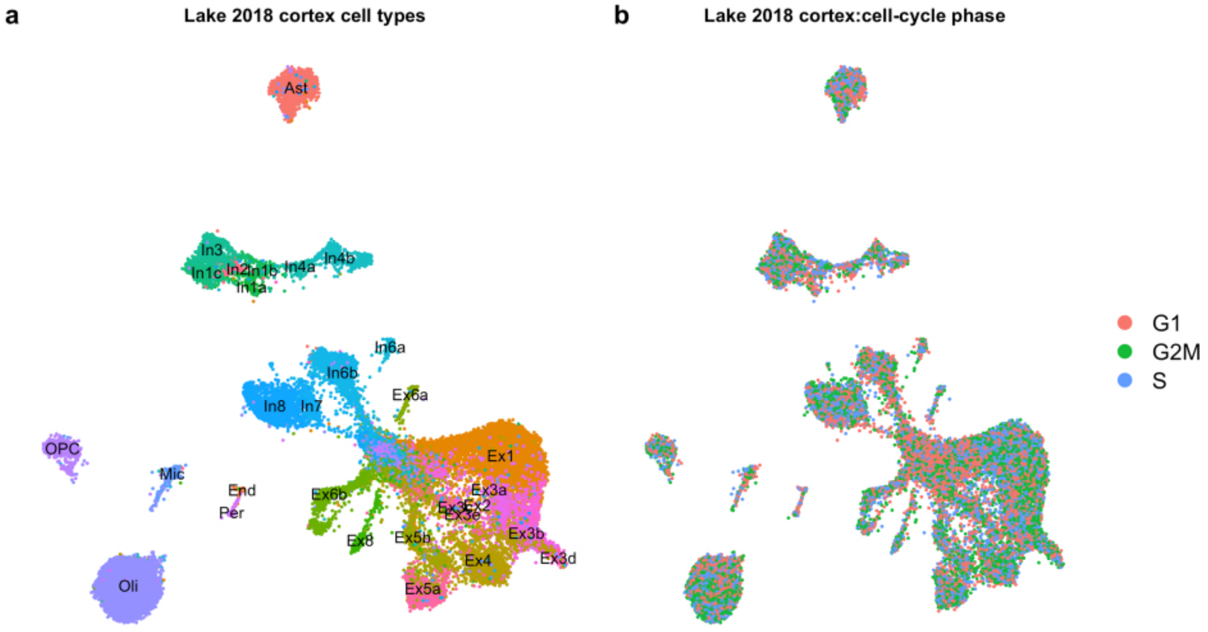

[Reviewer #4] •Key example networks showing DaN (Figure S10) and ODC modules (e.g. M5 or M1) associated with PD should be displayed as a panel in Figure 4 rather than in a supplementary figure.

[Authors] As requested by the Reviewer, we moved this Figure to main Figure of manuscript: it become Figure 5, As this network figure can be unreadable, we preferred make a separated Figure.

[Reviewer #4] Minor Comments:

1. **Line 109-110: A more concise and clearer subheading is needed.**

[Authors] We modified this header from:
"The single-cell profiles of the Substantia nigra inform on the cell types that the genetic risk of Parkinson's disease and other disorders likely manifests within"
to
"*Complex trait genetics highlights distinct nigral and cortical cell types*"

2. **Line 97: Should refer to Figure 1d & e instead of Supplemental Figures 1d & e.**

[Authors] Thanks for spotting this error. We have now corrected the text.

3. **Figure S5 is missing an explanation of what the sizes of the circles mean in this graph.**

[Authors] The circle size is proportional of the absolute value of the Pearson residual (left panel) or with % represented by chi^2 residual (right) panel. To clarify this Figure, we added the Pearson residual value and the % represented by chi^2. We also added an explanation in the text Legend of this Figure: The size of the circle is proportional to the absolute value of the Pearson residual or the % represented by chi^2 residual.

4. **The authors provide a clear description of their conditional analyses in the methods section, but a brief clarification in the main text would help readers more easily follow the conclusions drawn from these analyses (for ex: what does p < 0.05 mean in this context?).**

[Authors] As requested, we added a sentence to introduce the methodology of the conditional analysis in the result section

[Results p] "To evaluate whether the fraction of PD genetic risk associated with the DaN or OPC cell types was overlapping or distinct, we performed conditional analyses by running LDSC with either the ODC or the DaN gene set as the control: We found that the fraction of PD genetic risk contributing to the ODC association was distinct to that fraction associated with DaNs (Supplementary Table 6, p-value associated with an LDSC Coefficient < 0.05 (here $p=7x10^{-3}$)), proposing distinct PD-associated cell etiologies within the SN"

5. **The authors show that converging OPC/ODC genes are also associated with a range of psychiatric conditions. This should be mentioned in the abstract and/or introduction, as this is an important and novel result of this work.**

[Authors] Yes, we agree with the Reviewer that these findings are important for future research directions and have edited the Abstract to include this.

[Abstract]: We describe a human single-nuclei transcriptomic atlas for the Substantia nigra (SN), generated by sequencing ~ 17,000 nuclei from matched cortical and SN samples. We show that the common genetic risk for Parkinson's disease (PD) is associated with dopaminergic neuron (DaN)-specific gene expression, including mitochondrial functioning, protein folding and ubiquitination pathways. We identify a distinct cell type association between PD risk and oligodendrocyte-specific gene expression. Unlike Alzheimer's disease (AD), we find no association between PD risk and microglia or astrocytes, suggesting that neuroinflammation plays a less causal role in PD than AD. Beyond PD, we find associations between SN DaNs and GABAergic neuron gene expression and multiple neuropsychiatric disorders. Conditional analysis reveals that distinct neuropsychiatric disorders associate with distinct sets of neuron-specific genes **but converge onto shared loci within oligodendrocytes and oligodendrocyte precursor**s. This atlas guides our aetiological understanding by associating SN cell type expression profiles with specific disease risk**.**

References.

1. Bryois J, *et al.* Genetic identification of cell types underlying brain complex traits yields insights into the etiology of Parkinson's disease. *Nat Genet*, (2020).

2. Skene NG, *et al.* Genetic identification of brain cell types underlying schizophrenia. *Nat Genet* **50**, 825-833 (2018).

3. Watanabe K, Umicevic Mirkov M, de Leeuw CA, van den Heuvel MP, Posthuma D. Genetic mapping of cell type specificity for complex traits. *Nat Commun* **10**, 3222 (2019).

4. Nalls MA, *et al.* Identification of novel risk loci, causal insights, and heritable risk for Parkinson's disease: a meta-analysis of genome-wide association studies. *Lancet Neurol* **18**, 1091-1102 (2019).

5. Finucane HK, *et al.* Heritability enrichment of specifically expressed genes identifies disease-relevant tissues and cell types. *Nat Genet* **50**, 621-629 (2018).

6. Donovan MKR, D'Antonio-Chronowska A, D'Antonio M, Frazer KA. Cellular deconvolution of GTEx tissues powers discovery of disease and cell-type associated regulatory variants. *Nat Commun* **11**, 955 (2020).

7. Lake BB, *et al.* Integrative single-cell analysis of transcriptional and epigenetic states in the human adult brain. *Nat Biotechnol* **36**, 70-80 (2018).

REVIEWERS' COMMENTS:

Reviewer #3 (Remarks to the Author):

The authors have done a good job of responding to the referees' comments

Reviewer #4 (Remarks to the Author):

The authors have sufficiently addressed all my concerns, and I recommend the manuscript for publication in Nature Communications.
